# Syntheses, characterizations and reactions of acene-2,3-dicarbaldehydes†

**DOI:** 10.1039/d4ra04273e

**Published:** 2024-08-09

**Authors:** Qian Liu, Glen P. Miller

**Affiliations:** a Department of Chemistry, University of New Hampshire 23 Academic Way Durham New Hampshire 03824 USA glen.miller@unh.edu

## Abstract

Here, we report improved syntheses, detailed characterizations and reactions of a series of acene-2,3-dicarbaldehydes including tetracene-2,3-dicarbaldehyde. DFT calculations corroborate and complement the experimental results. Tetracene-2,3-dicarbaldehyde and the benchmark organic semiconductor pentacene have isoelectronic π-systems and similar HOMO–LUMO gaps. Tetracene-2,3-dicarbaldehyde is soluble in a host of organic solvents (*e.g.*, DMF, toluene, THF, chloroform, dichloromethane) and shows excellent photooxidative resistance in solution phases exposed to light and air. Further, it is readily sublimed from the solid-state without decomposition, and can be functionalized using different chemistries. We have demonstrated the utility of acene-2,3-dicarbaldehydes as reactants in the syntheses of novel α,α′-diaryl-2,3-acenedimethanols and acenotropones *via* Grignard reactions and double-aldol condensation reactions, respectively. The acenotropones were further reacted with concentrated H_2_SO_4_ to generate hydroxyacenotropylium ions that exhibit long wavelength absorption in the visible and near-IR regions. The optical gap measured for hydroxyanthracenotropylium ion is 1.3 eV. The results gained here implicate acene-2,3-dicarbaldehydes as potential high-value organic semiconductors and as precursors to a host of interesting molecules and materials.

## Abstract 2

Improved syntheses, detailed characterizations and reactions of a series of acene-2,3-dicarbaldehydes including tetracene-2,3-dicarbaldehyde are described.

## Graphical abstract

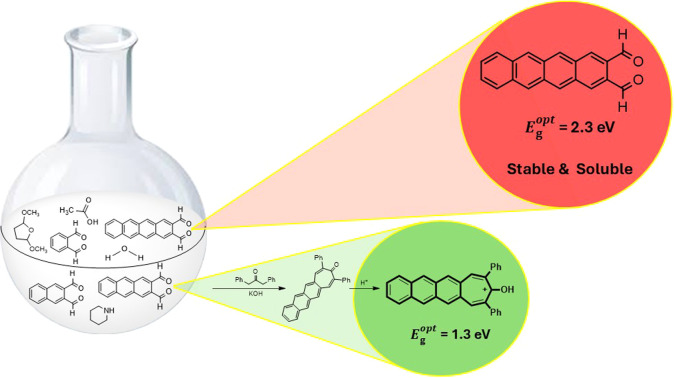

## 1 Introduction

Select polycyclic aromatic hydrocarbons (PAHs) and their derivatives have been utilized as organic semiconductors in various electronic devices including organic field-effect transistors (OFETs),^1–3^ organic light-emitting diodes (OLEDs),^4,5^ organic photovoltaics (OPVs),^6–8^ organic photodetectors (OPDs)^9–11^ and organic sensors.^12–15^ Acenes such as pentacene have been considered for all of these applications owing to their small HOMO–LUMO gaps and their high charge carrier mobilities.

Large acenes like pentacene are of interest because they exhibit relatively small HOMO–LUMO gaps and relatively high charge carrier mobilities. However, they suffer from several problems including (i) poor solubility in most solvents, and (ii) a propensity to photooxidize, especially when dissolved in solution phases exposed to light and air. With regards to (i), adequate solubility is important as it enables solution processing like spin coating, blade coating, spray coating, ink-jet printing, *etc.*, for the construction of thin-film electronic devices.^16,17^ With regards to (ii), pentacene undergoes rapid photooxidation with a half-life of only 7.5 minutes^18^ in solution phases exposed to light and air. Solution processing is complicated by an organic semiconductor like pentacene. It is most desirable to utilize acenes that are both soluble and resistant to photooxidation.

The solubilities and photooxidative resistances of acenes can be improved through the judicious choice of substituents.^18^ One strategy to slow the photooxidation of acenes is to add electron-withdrawing substituents to their backbones thereby lowering the energy of their HOMOs and making them altogether less reactive with singlet oxygen. Halogenated acenes,^16,17^ organothio substituted acenes^18–20^ and TIPS substituted acenes^17,21,22^ are examples of derivatives with electron-withdrawing substituents that demonstrate enhanced photooxidative resistance. Depending upon the number and type of electron-withdrawing substituents included, the acene may switch from p-type to n-type.^16,17^

**Fig. 1:**
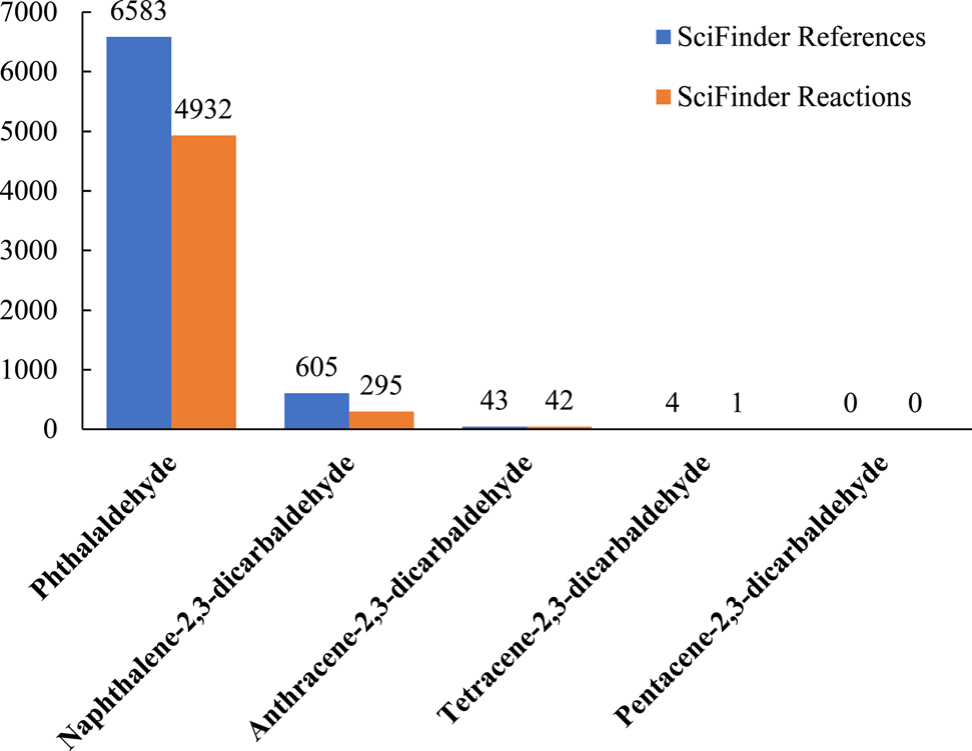
The number of references and reactions of acene-2,3-dicarbaldehydes reported on SciFinder up to and including May, 2024.

Aldehydes are well-known electron-withdrawing substituents and as such, they too should increase the photooxidative resistance of acenes. Aldehydes also react with a myriad of nucleophiles. Thus, it seems reasonable that aldehyde substituted acenes may show enhanced photooxidative resistance and also act as synthetic precursors for the synthesis of functional molecules and materials that include acene moieties. Indeed, *o*-phthalaldehyde maps directly onto larger acene-2,3-dicarbaldehydes like anthracene-2,3-dicarbaldehyde, tetracene-2,3-dicarbaldehyde and pentacene-2,3-dicarbaldehyde, and has proven useful for the synthesis of acene quinones,^18,22^ heteroarenes^22,23^ and isobenzoheteroles.^24,25^ The literature remains sparse, however, with regards to the synthesis, characterization and reactivity of naphthalene-2,3-dicarbaldehyde, anthracene-2,3-dicarbaldehyde, tetracene-2,3-dicarbaldehyde and pentacene-2,3-dicarbaldehyde (Fig. 1). Only one synthesis of tetracene-2,3-dicarbaldehyde with limited (*i.e.*, UV-vis) characterization has been reported^26^ and there are no reported syntheses of pentacene-2,3-dicarbaldehyde.

**Scheme 1:**
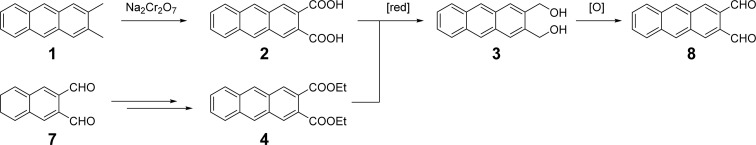
Conventional methods to synthesize anthracene-2,3-dicarbaldehyde, 8, and by analogy tetracene-2,3-dicarbaldehyde, 9.

A conventional synthetic plan for the formation of anthracene-2,3-dicarbaldehyde, 8, is illustrated in Scheme 1. Thus, Friedman demonstrated^27^ a sodium dichromate oxidation of 2,3-dimethylnapthalene to produce 2,3-naphthalenedicarboxylic acid in 87–93% yield. An analogous reaction starting with 2,3-dimethylanthracene, 1, should produce anthracene-2,3-dicarboxylic acid, 2. A borane-THF reduction of diacid 2 to anthracene-2,3-dimethanol 3 was demonstrated in near quantitative yield by Seo and co-workers.^28^ Alternatively, diethyl anthracene-2,3-dicarboxylate, 4, could be utilized. From naphthalene-2,3-dicarbaldehyde, 7, compound 4 was synthesized in two steps by Lin and co-workers^29^ and subsequently reduced to diol 3 in 77% yield using DIBAL. Wang and co-workers demonstrated separate mechanochemical IBX oxidations of naphthalene-2,3-dimethanol and 3 yielding naphthalene-2,3-dicarbaldehyde, 7, and anthracene-2,3-dicarbaldehyde, 8, respectively, in 81% yields.^30^

**Scheme 2:**
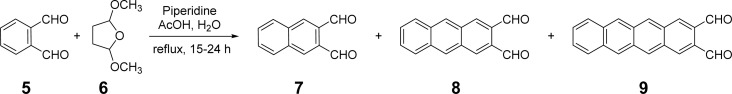
A one-pot synthesis of acene-2,3-dicarbaldehydes demonstrated by Mallouli and Lepage^26^ leading to mixtures of naphthalene-2,3-dicarbaldehyde, 7, anthracene-2,3-dicarbaldehyde, 8, and tetracene-2,3-dicarbaldehyde, 9.

While a synthetic plan like that outlined in Scheme 1 seemingly could be modified for the synthesis of tetracene-2,3-dicarbaldehyde, 9, or larger acene-2,3-dicarbaldehydes, no such syntheses have been reported. Scheme 1 includes high-yielding reactions but it is nonetheless a multi-step synthesis utilizing starting materials that must either be synthesized in multiple steps or purchased at high cost. Alternatively, a method reported by Mallouli and Lepage^26^ for the synthesis of multiple acene-2,3-dicarbaldehydes utilizes a one-pot procedure involving only low cost, readily available starting materials: 5, 2,5-dimethoxytetrahydrofuran, 6, and piperidine (Scheme 2).

Lin and co-workers properly noted a problem^29^ with the one-pot method of Mallouli and Lepage,^26^ namely a lack of selectivity. Thus, acene-2,3-dicarbaldehydes 7, 8 and 9 are all formed in the same pot with little selectivity. Of these, 9 is the most interesting as a potential organic semiconductor and likewise, a similar method to prepare it in good yield with good selectivity is desirable.

In this study, we modified the procedure of Mallouli and Lepage^26^ such that acene-2,3-dicarbaldehydes including anthracene-2,3-dicarbaldehyde, 8, and tetracene-2,3-dicarbaldehyde, 9, were prepared in higher combined yield. We also report conditions where 9 can be prepared with vastly improved selectivity. Our work shines a spotlight on tetracene-2,3-dicarbaldehyde, 9, which has avoided attention and a detailed characterization, until now. It has a π-system that is isoelectronic with pentacene and a similar HOMO–LUMO gap, but with far more agreeable properties. Dicarbaldehyde 9 is soluble in a host of organic solvents (*e.g.*, DMF, toluene, THF, chloroform, dichloromethane) and shows excellent photooxidative resistance in solution phases exposed to light and air. Further, the dicarbaldehydes described here can be sublimed from the solid-state without decomposition, and can be functionalized using different chemistries to produce novel structures with interesting properties.

## 2 Results and discussion

### 2.1 A plausible reaction sequence for the one-pot synthesis of acene-2,3-dicarbaldehydes

The one-pot synthesis of acene-2,3-dicarbaldehydes demonstrated by Mallouli and Lepage^26^ is illustrated in Scheme 2. Thus, 5 and 6 are heated in aqueous acetic acid in the presence of a few drops of piperidine. All three acene-2,3-dicarbaldehydes (7, 8 and 9) were formed but with little selectivity and without thorough characterization.

**Scheme 3:**
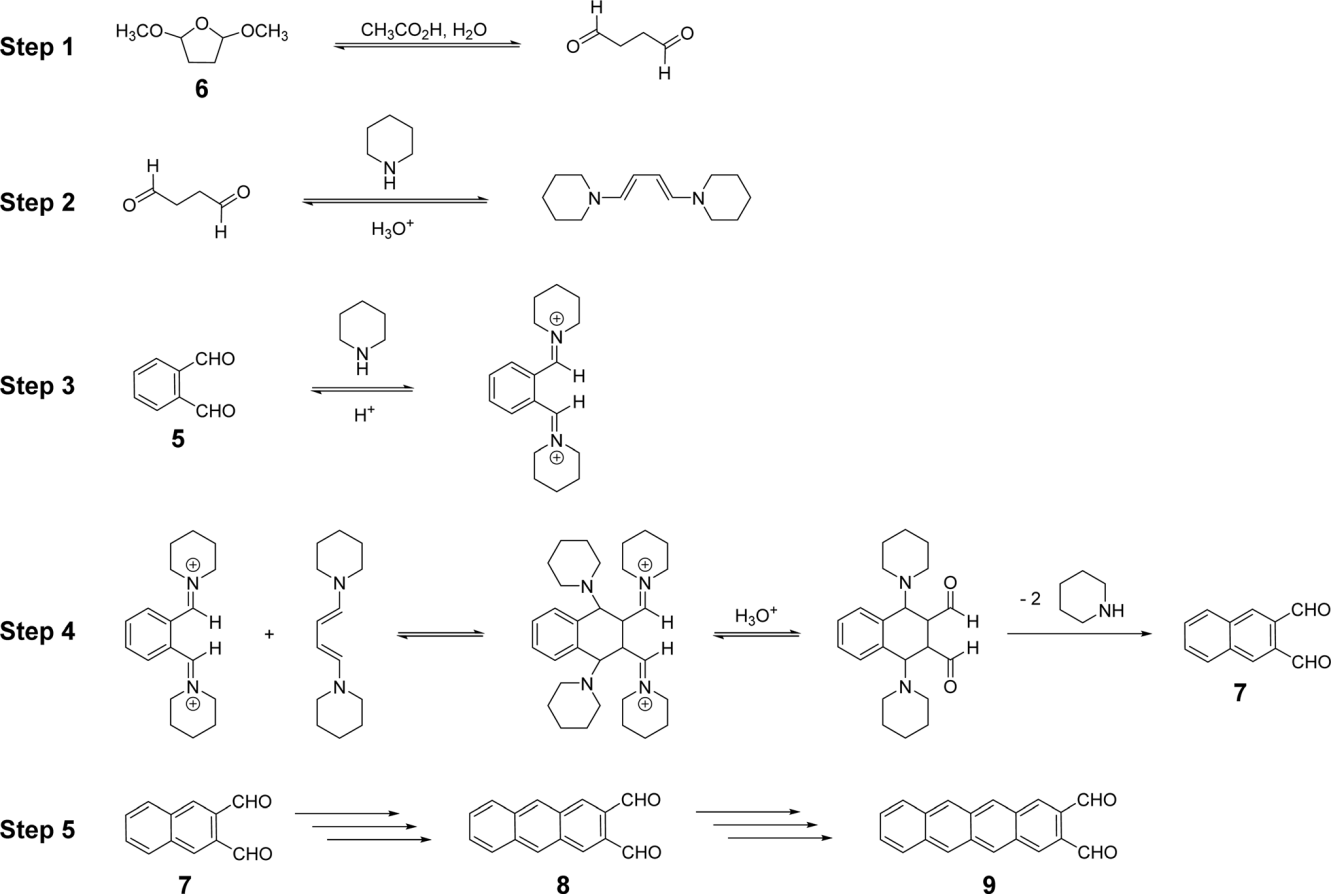
A plausible, iterative reaction sequence leading to naphthalene-2,3-dicarbaldehyde, 7, and by analogy, anthracene-2,3-dicarbaldehyde, 8, and tetracene-2,3-dicarbaldehyde, 9.

A plausible reaction sequence for this multi-stage, one-pot synthesis is proposed in Scheme 3. First, 6 can be converted reversibly to succinaldehyde under the acidic reaction conditions employed (step 1). Succinaldehyde can react reversibly with piperidine to form a conjugated 1,3-diene-1,4-diamine with nucleophilic α-carbons (step 2). Simultaneously, 5 can react reversibly with piperidine to produce an iminium ion intermediate with highly electrophilic carbons (step 3). The products of steps 2 and 3 can undergo reversible polar additions to form an intermediate which, upon *irreversible* elimination of multiple piperidine equivalents, yields 7 (step 4). In iterative fashion, 7, and eventually 8, can react with 6 and piperidine to form 8 and 9, respectively (step 5). The reaction essentially stops at the tetracene-2,3-dicarbaldehyde stage due to the poor solubility of 9 in the reaction medium (*vide infra*). Scheme 3 represents a plausible reaction sequence, not a detailed mechanism. For example, it is not necessary or even likely that both iminium ions generated from 5 form at the same time as in step 3. Likewise, it is not necessary that both enamines form in step 2 before reaction with an iminium ion, and so on.

### 2.2 An improved one-pot synthesis of acene-2,3-dicarbaldehydes

In experiments 1–3 of Table 1, we utilized essentially the same reaction conditions as employed by Mallouli and Lepage.^26^ Upon considering Scheme 3, we modified these conditions to increase the relative concentrations of 6 and piperidine, and by allowing for longer reaction times (experiments 4 and 5 in Table 1). In doing so, we observed the formation of 8 and 9 in greater combined yield (experiments 4 and 5) and found conditions leading to the highly selective formation of 9 (experiment 5). The conditions of experiment 5 in Table 1 lead to 9 in 48% yield, with only traces of other aceno-2,3-dicarbaldehydes present. Thus, according to the proposed reaction pathway of Scheme 3, increasing the concentrations of 6 and piperidine should lead to larger equilibrium concentrations of enamine and iminium ion reactants (steps 3 and 4 of Scheme 3). Longer reaction times (experiments 4 and 5 of Table 1) allow for iterative growth of acene-2,3-dicarbaldehydes up to but not beyond 9. Pentacene-2,3-dicarbaldehyde and larger acene-2,3-dicarbaldehydes were not observed to form, likely due to the poor solubility of 9 in the reaction medium. The irreversible elimination of piperidine (step 4 of Scheme 3) combined with the poor solubility of 9 in the reaction medium enables the highly selective formation and accumulation of 9, nearly free of other aceno-2,3-dicarbaldehydes.

**Table 1:** Synthesis of acene-2,3-dicarbaldehydes

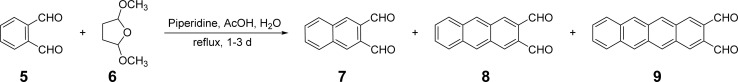								
Exp.	5 : 6^a^	Piperidine	AcOH	H_2_O	Time/h	7	8	9
1	1 : 1	2 drops	1 mL	1.5 mL	18	30%	9%	—
2	1 : 2	3 drops	1.5 mL	1.5 mL	24	17%	25%	—
3	1 : 4	4 drops	3 mL	1 mL	24	10%	15%	21%
4	1 : 6	5 drops	4 mL	1 mL	24	7%	20%	25%
5	1 : 8	5 drops	4 mL	1 mL	72	—	Trace	48%

a Two grams (15 mmol) of 5 were utilized in each experiment. The ratios of 5 : 6 are molar ratios. All product percent yields refer to isolated product, after purification.

In addition to modifying the reactions conditions to produce 8 and 9 in higher combined yield, we found overall improved yields of acene-2,3-dicarbaldehydes upon modifying the work-up procedure reported by Mallouli and Lepage.^26^ Specifically, we noted product losses during vacuum filtration when using methanol or ether as wash solvents. To address this, we collected the filtrate suspension and subjected it to a second vacuum filtration to obtain a second-batch of filtered solid product. First-batch and second-batch filtered solids were subsequently sublimed at reduced pressures. Additional details are provided in the ESI.†

Acene-2,3-dicarbaldehydes 7, 8, and 9 are all robust molecules, even when subjected to elevated temperatures for prolonged times. As such, they may be separated from one another *via* high-temperature vacuum sublimations. We observe 132 °C/0.1 Torr/4–6 hours to be optimal conditions for the sublimation of 7, 180 °C/0.1 Torr/4–6 hours to be optimal conditions for the sublimation of 8, and 220 °C/0.1 Torr/4–12 hours to be optimal conditions for the sublimation of 9. It should also be noted that acene-2,3-dicarbaldehydes 5, 7, 8 and 9 are soluble in a variety of organic solvents (*e.g.*, DMF, toluene, THF, chloroform and dichloromethane) and all show indefinite stability in solution phases exposed to light and air.

### 2.3 Optical properties of acene-2,3-dicarbaldehydes

**Fig. 2:**
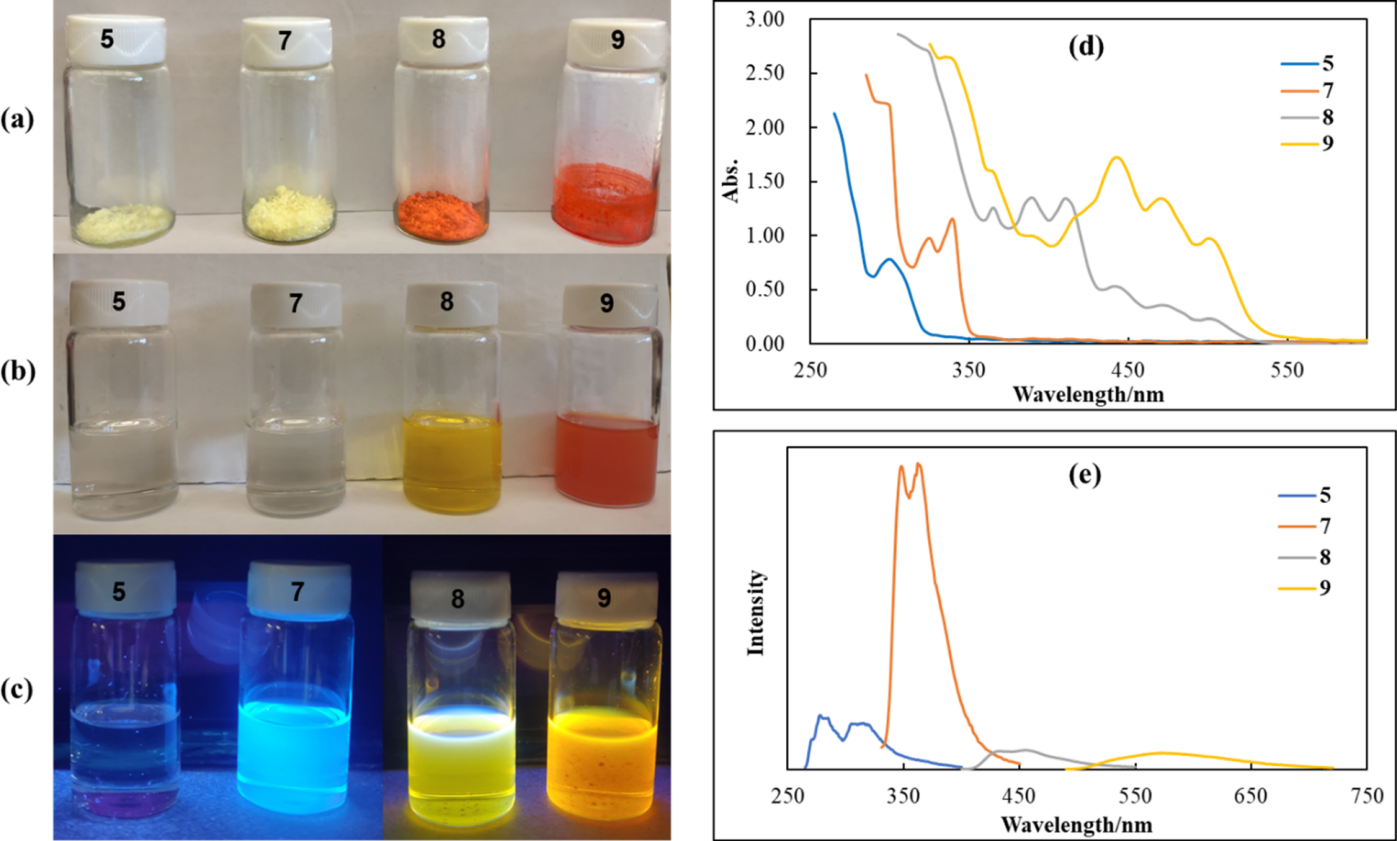
Acene-2,3-dicarbaldehydes: (a) in the solid state; (b) 1 × 10^−3^ M solutions in CH_2_Cl_2_ under room light; (c) 1 × 10^−3^ M solutions in CH_2_Cl_2_ irradiated with 365 nm UV light; (d) UV-vis spectra of 1 × 10^−4^ M solutions in CH_2_Cl_2_; (e) fluorescence spectra of 2 × 10^−6^ M solutions in CH_2_Cl_2_.

The optical properties of acene-2,3-dicarbaldehydes 5, 7, 8 and 9 are illustrated in Fig. 2. In the solid state, the color of 5, 7, 8 and 9 graduate from pale yellow to deep orange/red (Fig. 2a). Solutions of acene-2,3-dicarbaldehydes 5 and 7 are essentially colorless under room light while those of 8 and 9 are golden yellow and orange/red, respectively (Fig. 2b). Upon irradiation with 365 nm UV light, 5, 7, 8 and 9 fluoresce leaving solutions that are purple, sky blue, golden yellow and orange/red in color (Fig. 2c). UV-vis spectra indicate that 5, 7, 8 and 9 have broad absorptions in the range of 250–550 nm with *λ*_max_ values at 300, 340, 410 and 503 nm, respectively (Fig. 2d). The red-shifting *λ*_max_ values for these molecules provide strong evidence for progressively smaller HOMO–LUMO gaps upon moving from 5 to 7 to 8 to 9. Fluorescence spectra show emissions in the range of 250–700 nm with maximum *λ*_em_ values of 315, 363, 455 and 572 nm for 5, 7, 8 and 9, respectively (Fig. 2e). Notably, 7 exhibits the most intensive fluorescence emissions.

### 2.4 DFT calculations of acene-2,3-dicarbaldehydes

**Fig. 3:**
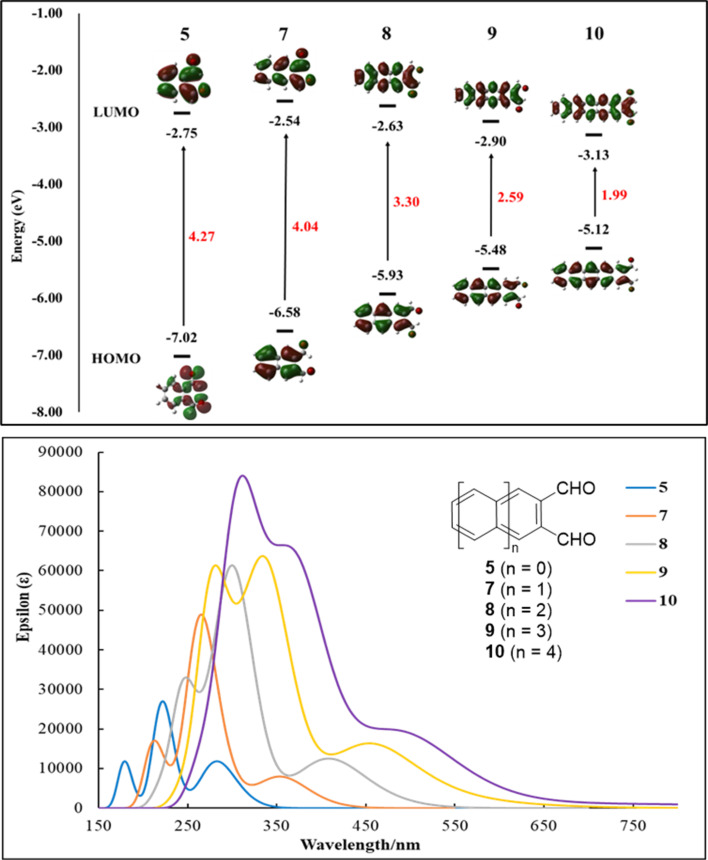
Calculated HOMO and LUMO orbitals (top) and UV-vis spectra (bottom) for acene-2,3-dicarbaldehydes 5, 7, 8, 9 and 10. Solvent model (dichloromethane) TD-DFT calculations were performed at the B3LYP-D3(BJ)/6-311+G(d,p)/SMD(DCM)//B3LYP-D3(BJ)/6-31G(d)/SMD(DCM) level of theory using the Gaussian 09 program.

To gain further insight into the structure and electronic properties of acene-2,3-dicarbaldehydes, time-dependence DFT (TD-DFT) calculations were performed at the B3LYP-D3(BJ)/6-311+G(d,p)/SMD(DCM)//B3LYP-D3(BJ)/6-31G(d)/SMD(DCM) level of theory using the Gaussian 09 program.^31^ Frontier orbital energy levels, HOMO–LUMO gaps, orbital distributions and UV-Vis spectra are shown in Fig. 3. The HOMO and LUMO orbital densities for acene-2,3-dicarbaldehydes 5, 7–9 and pentacene-2,3-dicarbaldehyde 10 are spread throughout each molecule including their terminal aldehyde groups (Fig. 3, top), consistent with highly conjugated, highly delocalized π-systems. Similar to unsubstituted acenes, as the number of atoms-in-conjugation grows, LUMO energies decrease slightly while HOMO energies show a modest rise. The result is a significant lowering of the HOMO–LUMO gaps from 4.27 eV to 1.99 eV (Fig. 3, top) and progressively longer wavelengths of absorptions (Fig. 3, bottom) as one progresses from 5 to 7 to 8 to 9 to 10. This trend is in good agreement with experimental results as shown in Table 2 where a detailed collection of optical and DFT calculated properties for acene-2,3-dicarbaldehydes is assembled.

**Table 2:** Optical and DFT calculating properties of acene-2,3-dicarbaldehydes

Compd	*λ* ^Abs(DFT)^_max_/nm	*λ* ^Abs^_max_/nm	*λ* ^Em^_max_/nm	*λ*_onset_/nm	***E*** ^**opt**^_**g**_ **/eV**	*E* ^DFT^_LUMO_/eV	*E* ^DFT^_HOMO_/eV	***E*** ^**DFT**^_**g**_ **/eV**
5	284	300	315	324	**3.8**	−2.75	−7.02	**4.27**
7	355	340	363	349	**3.6**	−2.54	−6.58	**4.04**
8	407	410	455	437	**2.8**	−2.63	−5.93	**3.30**
9	455	503	572	533	**2.3**	−2.90	−5.48	**2.59**
10	494	—	—	—	—	−3.13	−5.12	**1.99**

UV-vis spectra were recorded at 1 × 10^−4^ M in CH_2_Cl_2_ solution; fluorescence spectra were recorded at 2 × 10^−6^ M in CH_2_Cl_2_ solution; optical energy gaps were determined from the onset wavelengths (*λ*_onset_) associated with the lowest-energy absorption bands; the onset is defined as the intersection between the baseline and a tangent line that touches the point of inflection; solvent model (dichloromethane) TD-DFT calculations were performed at the B3LYP-D3(BJ)/6-311+G(d,p)/SMD(DCM)//B3LYP-D3(BJ)/6-31G(d)/SMD(DCM) level of theory using the Gaussian 09 program.

**Fig. 4:**
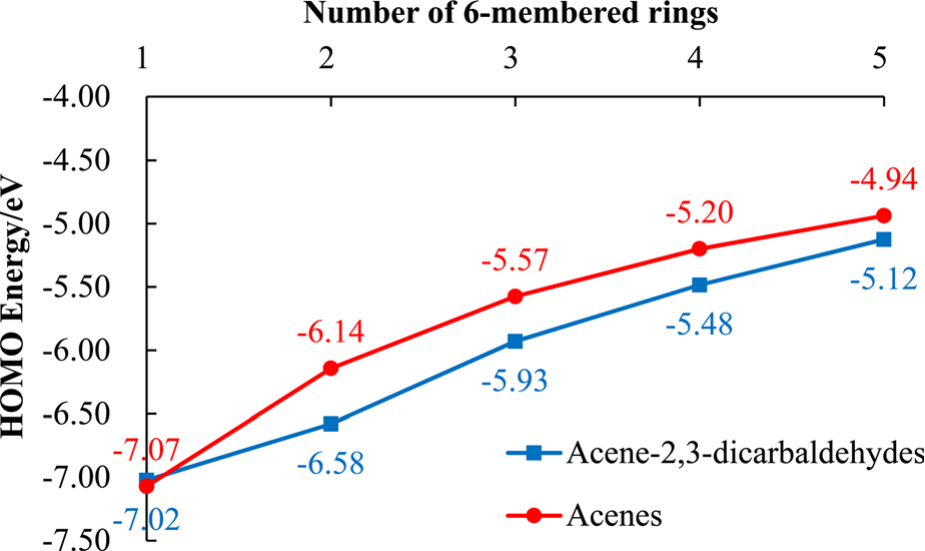
HOMO energies for both acene-2,3-dicarbaldehydes (blue data points, blue line) and the corresponding unsubstituted acenes (red data points, red line) plotted against the number of 6-membered rings in each molecule. Solvent model (dichloromethane) TD-DFT calculations were performed at the B3LYP-D3(BJ)/6-311+G(d,p)/SMD(DCM)//B3LYP-D3(BJ)/6-31G(d)/SMD(DCM) level of theory using the Gaussian 09 program.

As mentioned above, 5, 7, 8 and 9, each with a pair of electron-withdrawing aldehyde substituents, show excellent stability in the solid-state and in solution phases exposed to light and air. Large acenes (*i.e.*, tetracene and larger) are known to sensitize singlet oxygen, ^1^O_2_, formation. This highly reactive oxygen species can then undergo a [4 + 2] cycloaddition across an embedded diene of the large acene.^18^ Electron-withdrawing substituents that lower HOMO energies can create a larger HOMO_acene_–LUMO_oxygen_ energy difference, thereby slowing the rate of concerted cycloaddition. A comparison of calculated HOMO energies for acene-2,3-dicarbaldehydes and their corresponding unsubstituted acenes (Fig. 4) is informative. Thus, the HOMO energies associated with acene-2,3-dicarbaldehydes with two or more rings are lower by 0.2–0.4 eV than the corresponding unsubstituted acenes (Fig. 4) and this likely accounts for their reduced reactivity with singlet oxygen.

It is worth noting that even with a modest lowering of their HOMO energies and improved photooxidative resistances, 9 and 10 both possess HOMO energies (Table 2, Fig. 2 and 3) that are consistent with some of the best known p-type organic semiconductors.^32^ Therefore, while it benefits from enhanced solubility and photooxidative resistance, we do not expect a switch from p-type to n-type organic semiconductor behavior for 9.

### 2.5 Further reactions of acene-2,3-dicarbaldehydes

#### 2.5.1 Grignard reactions leading to novel α,α′-diaryl-2,3-acenedimethanols

**Scheme 4:**
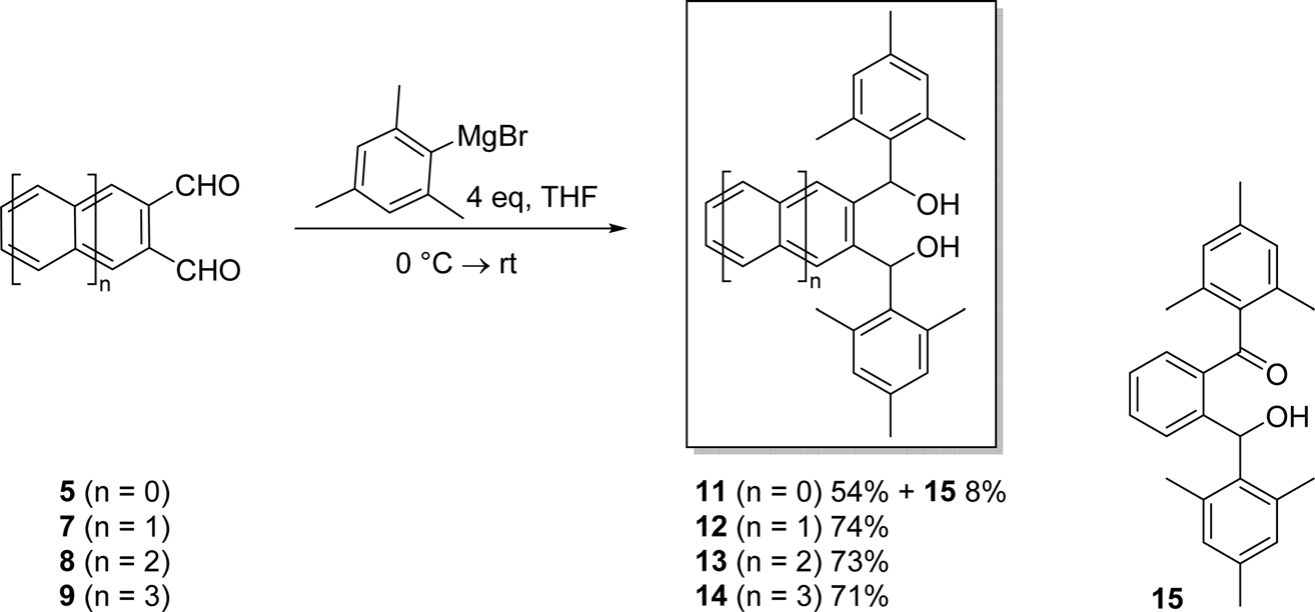
Synthesis of novel α,α′-dimesityl-2,3-acenedimethanols 11–14.

α,α′-Diaryl-2,3-acenedimethanols are intermediates in the syntheses of isoacenofurans^33,34^ and large acenes.^35,36^ In addition, they could be used to make alkali metal-chelate complexes.^37,38^ We reacted a Grignard reagent, mesityl magnesium bromide, with our acene-2,3-dicarbaldehydes (5, 7–9) to obtain a set of α,α′-dimesityl-2,3-acenedimethanols (11–14) as illustrated in Scheme 4.

Thus, diol 11 was formed in 54% yield by reacting 5 with mesityl magnesium bromide at 0 °C followed by stirring at room temperature for 5 hours. The ketone-alcohol by-product 15 was also formed in 8% yield, presumably *via* air oxidation of initially formed 11. Acene-2,3-dicarbaldehydes 7–9 were also reacted with mesityl magnesium bromide at 0 °C followed by stirring at 50 °C for 2 hours and then stirring at room temperature for 24 hours. In this way, novel α,α′-dimesityl-2,3-acenedimethanols 12–14 were obtained in good yields ranging from 71 to 74%. Ketone-alcohols akin to 15 were not observed in these reactions. Although acene-2,3-dicarbaldehydes are all soluble in a host of organic solvents, α,α′-dimesityl-2,3-acenedimethanols 11–14 are highly soluble in many of the same solvents. Their excellent solubilities are likely due to the conformational requirement that the terminal mesityl groups rotate out of the plane, thus reducing intermolecular π–π stacking that can otherwise promote agglomeration and precipitation.

#### 2.5.2 Double-aldol condensations of acene-2,3-dicarbaldehydes leading to novel acenotropones

**Scheme 5:**
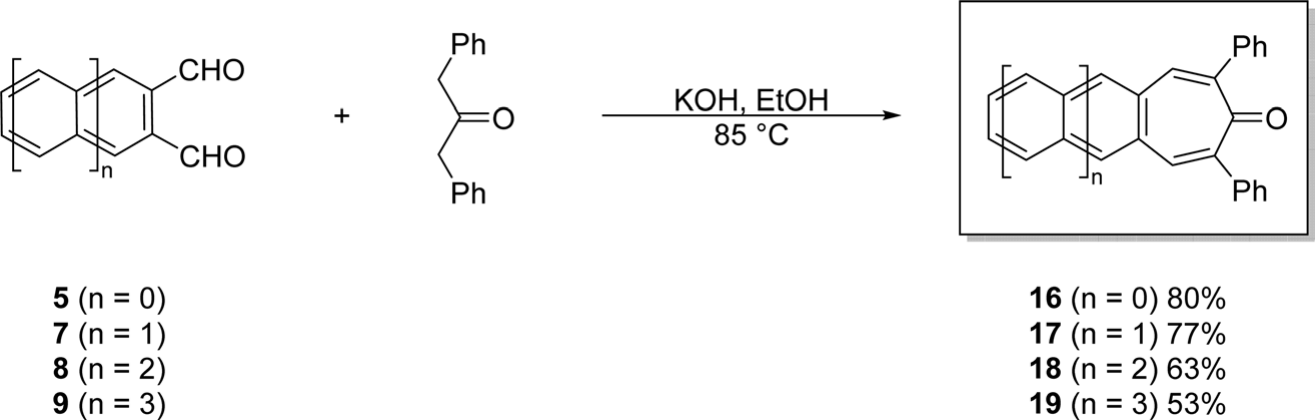
Efficient, one-pot syntheses of acenotropones 16–19*via* double-aldol condensations of acene-2,3-dicarbaldehydes 5 and 7–9.

Acenotropones are highly interesting but largely unexplored species that can be used to prepare acenotropylium salts,^39^ or utilized themselves as a novel class of n-type organic semiconductor.^36^ Benzotropones^40–42^ and naphthotropones^43,44^ have been studied, but anthracenotropones and tetracenotropones are unknown. Here, we demonstrate efficient syntheses of acenotropones 16–19 using a one-pot, double-aldol condensation reaction between acene-2,3-dicarbaldehydes and 1,3-diphenylacetone^45^ (Scheme 5).

These base-mediated reactions afford the corresponding 2,7-diphenyl-4,5-acenotropones 16–19 in yields ranging from 53 to 80%. Combined with a simple work up (vacuum filtration followed by solvent washes), the reactions are scalable. The color of the acenotropones deepen from yellow to red with increasing acene length. Each of the 2,7-diphenyl-4,5-acenotropones, 16–19, are stable in the solid-state. However, unlike its precursor 9, tetracenotropone 19 does photooxidize if left in solution phases exposed to light and air.

**Fig. 5:**
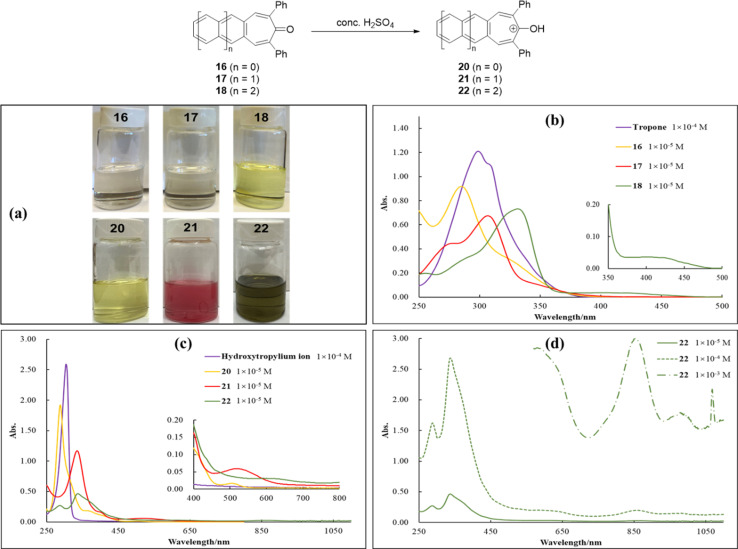
(a) 1 × 10^−4^ M solutions of acenotropones 16–18 in CH_2_Cl_2_ solution and 1 × 10^−4^ M solutions of hydroxyacenotropylium ions 20–22 in conc. H_2_SO_4_; (b) UV-vis spectra of tropone and acenotropones 16–18 in CH_2_Cl_2_ solution; (c) UV-vis spectra of hydroxytropylium ion and hydroxyacenotropylium ions 20–22 in conc. H_2_SO_4_; (d) UV-vis spectra of hydroxyanthracenotropylium ion 22 at several different concentrations in conc. H_2_SO_4_.

We were compelled to react acenotropones 16–18 with concentrated H_2_SO_4_ in order to generate the corresponding hydroxyacenotropylium ions 20–22 (Fig. 5). Acenotropylium ions are highly interesting, unexplored molecules with potential applications as n-type organic semiconductors in organic electronics. While 1 × 10^−4^ M solutions of acenotropones 16–18 in CH_2_Cl_2_ are either colorless (16 and 17) or yellow (18), the corresponding hydroxyacenotropylium ions generated in concentrated H_2_SO_4_, 20–22, are yellow (20), red (21) and green (22), respectively (Fig. 5a). The UV-vis spectra for acenotropones 16–18 in CH_2_Cl_2_ solution (Fig. 5b) show absorptions in the range of 250–500 nm (*λ*_max_ values at 284, 306 and 402 nm, respectively) while those for the corresponding hydroxyacenotropylium ions 20–22 (Fig. 5c) are significantly red-shifted (*λ*_max_ values of 506, 524 and 978 nm, respectively). It is noteworthy that hydroxyanthracenotropylium ion 22 shows weak but dramatically red-shifted absorptions in the near-IR region with *λ*_max_ values of 860 and 978 nm (Fig. 5d). The optical gap measured for 22 is a mere 1.3 eV, placing it in the rare category of organic semiconductors with optical *E*_g_ values below 1.5 eV. For comparison, we observe that unsubstituted tropone has a *λ*_max_ value of 298 nm in CH_2_Cl_2_ (Fig. 5b, purple line) while the hydroxytropylium ion prepared in concentrated H_2_SO_4_ (Fig. 5c, purple line) shows a slight red shift (*λ*_max_ = 304 nm), consistent with a literature report.^46^

**Fig. 6:**
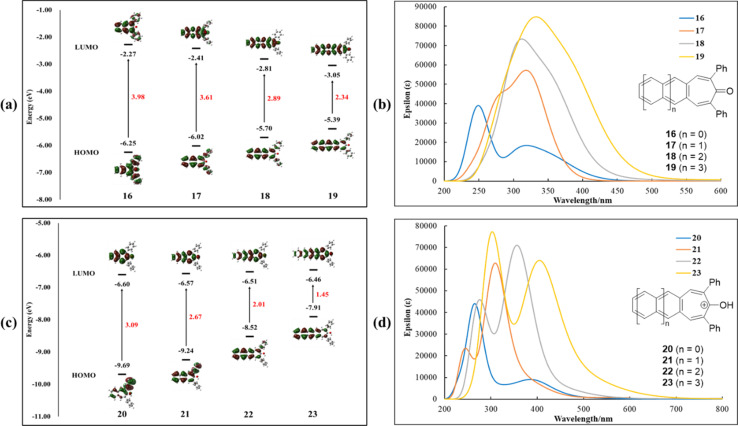
Calculated HOMO and LUMO orbitals and UV-Vis spectra for acenotropones 16–19 (a and b) and hydroxyacenotropylium ions 20–23 (c and d). Gas phase TD-DFT calculations were performed at the B3LYP/6-311+G(d,p)//B3LYP/6-31G(d) level of theory using the Gaussian 09 program.

To further investigate the structure and electronic properties of acenotropones 16–19 and hydroxyacenotropylium ions 20–23, gas phase time-dependence DFT (TD-DFT) calculations were performed at the B3LYP/6-311+G(d,p)//B3LYP/6-31G(d) level using the Gaussian 09 program.^31^ Frontier orbital energy levels, HOMO–LUMO gaps, orbital distributions and UV-Vis spectra were all calculated (Fig. 6). HOMO and LUMO orbital densities for acenotropones 16–19 and hydroxyacenotropylium ions 20–23 are spread throughout each molecule (Fig. 6a and c), consistent with highly conjugated, highly delocalized π-systems. Interestingly, the phenyl substituents show progressively less HOMO and LUMO orbital densities with increasing acene size (Fig. 6a and c) despite similar degrees of rotation out of plane. Similar to the acene-2,3-dicarbaldehydes of Fig. 3, a significant lowering of HOMO–LUMO gaps is observed for both acenotropones 16–19 (3.98 eV for 16; 2.34 eV for 19) and hydroxyacenotropylium ions 20–23 (3.09 eV for 20; 1.45 eV for 23) as the number of rings-in-conjugation grows (Fig. 6a and c). Likewise, the calculated wavelengths of absorption increase as the number of rings-in-conjugation grows (Fig. 6b and d). The shortest calculated HOMO–LUMO gaps and the longest calculated wavelengths of absorption are observed for the hydroxyacenotropylium ions (Fig. 6b and d).

A detailed collection of optical and DFT calculated properties for acenotropones 16–19 and hydroxyacenotropylium ions 20–23 is assembled in Table 3. DFT calculated HOMO–LUMO gaps for the neutral acenotropones 16–18 are in good agreement with experimental results. However, the DFT calculated HOMO–LUMO gaps for the charged hydroxyacenotropylium ions 20–22 are roughly 0.7 eV higher than those measured experimentally. The discrepancy may be due, in part, to the gas phase calculations employed for the charged hydroxyacenotropylium ions 20–22 (Fig. 6 and Table 3). In the future, we plan to compare and contrast calculated structures and electronics associated with (i) gas phase, (ii) polar solvent phase and (iii) solid-state structures containing multiple hydroxyacenotropylium salts.

**Table 3:** Optical and DFT calculating properties of acenotropones and hydroxyacenotropylium ions^a^

Compd	*λ* ^Abs(DFT)^_max_/nm	*λ* ^Abs^_max_/nm	*λ*_onset_/nm	***E*** ^**opt**^_**g**_ **/eV**	*E* ^DFT^_LUMO_/eV	*E* ^DFT^_HOMO_/eV	***E*** ^**DFT**^_**g**_ **/eV**
16	320, 248	284	322	**3.9**	−2.27	−6.25	**3.98**
17	319, 275	306	344	**3.6**	−2.41	−6.02	**3.61**
18	360, 310	402, 332	458	**2.7**	−2.81	−5.70	**2.89**
19	385, 334	—	—	—	−3.05	−5.39	**2.34**
20	386, 266	506, 288	546	**2.3**	−6.60	−9.69	**3.09**
21	311, 243	524, 336	650	**1.9**	−6.57	−9.24	**2.67**
22^b^	358, 276	978, 860, 584, 338	982	**1.3**	−6.51	−8.52	**2.01**
23	403, 300	—	—	—	−6.46	−7.91	**1.45**

a UV-Vis spectra of acenotropones were recorded at 1 × 10^−5^ M in CH_2_Cl_2_ while hydroxyacenotropylium ions 20 and 21 were recorded at 1 × 10^−5^ M in conc. H_2_SO_4_.

b The UV-vis spectrum of 22 was recorded at 1 × 10^−3^ M in conc. H_2_SO_4_. Optical energy gaps were determined from the onset of the lowest-energy absorption band (*λ*_onset_), the onset is defined as the intersection between the baseline and a tangent line that touches the point of inflection. Gas phase TD-DFT calculations were performed at the B3LYP/6-311+G(d,p)//B3LYP/6-31G(d) level of theory using the Gaussian 09 program.

## 3 Conclusions

A series of acene-2,3-dicarbaldehydes including anthracene-2,3-dicarbaldehyde, 8, and tetracene-2,3-dicarbaldehyde, 9, have been synthesized, purified and characterized using an improved one-pot synthetic method. The dicarbaldehydes are all fluorophores that show good solubility and excellent photooxidative resistance in solution phases exposed to light and air. Experimental and computational (DFT) results show that acene-2,3-dicarbaldehydes possess red-shifted UV-vis absorptions and progressively smaller HOMO–LUMO gaps as the number of rings-in-conjugation grows. Dicarbaldehydes 8 and 9 possess optical HOMO–LUMO gaps of 2.8 and 2.3 eV, respectively. Dicarbaldehyde 9 and the benchmark organic semiconductor pentacene have isoelectronic π-systems and similar HOMO–LUMO gaps. Dicarbaldehyde 9 is soluble in a host of organic solvents (*e.g.*, DMF, toluene, THF, chloroform, dichloromethane) and is readily sublimed from the solid-state at 220 °C without decomposition. We demonstrated the synthetic utility of acene-2,3-dicarbaldehydes by reacting them with mesityl magnesium bromide to form novel α,α′-dimesityl-2,3-acenedimethanols in 54–74% yield, and by reacting them with 1,3-diphenylacetone in one-pot, double-aldol condensation reactions to form acenotropones in 53–80% yield. The acenotropones were further reacted with concentrated H_2_SO_4_ to generate hydroxyacenotropylium ions that show long wavelength absorptions in the visible and near-IR regions. The optical gap measured for hydroxyanthracenotropylium ion, 22, is a mere 1.3 eV.

## Data availability

ESI† is available and includes experimental data, ^1^H NMR spectra, ^13^C NMR spectra and high-resolution mass spectra (HRMS) for key compounds.

## Conflicts of interest

There are no conflicts to declare.

## Supplementary Material

RA-014-D4RA04273E-s001

## References

[cit1] Quinn J. T. E., Zhu J., Li X., Wang J., Li Y. (2017). J. Mater. Chem. C.

[cit2] Wang C., Dong H., Hu W., Liu Y., Zhu D. (2012). Chem. Rev..

[cit3] Zheng C., Tong T., Hu Y., Gu Y., Wu H., Wu D., Meng H., Yi M., Ma J., Gao D., Huang W. (2018). Small.

[cit4] Yu T., Liu L., Xie Z., Ma Y. (2015). Sci. China: Chem..

[cit5] Zhu X.-H., Peng J., Cao Y., Roncali J. (2011). Chem. Soc. Rev..

[cit6] Kan B., Kan Y., Zuo L., Shi X., Gao K. (2021). InfoMat.

[cit7] Mishra A., Bäuerle P. (2012). Angew. Chem., Int. Ed..

[cit8] Inganäs O. (2018). Adv. Mater..

[cit9] Miao J., Zhang F. (2019). Laser Photonics Rev..

[cit10] Yang D., Ma D. (2019). Adv. Opt. Mater..

[cit11] Ren H., Chen J.-D., Li Y.-Q., Tang J.-X. (2021). Adv. Sci..

[cit12] Torsi L., Magliulo M., Manoli K., Palazzo G. (2013). Chem. Soc. Rev..

[cit13] Berggren M., Richter-Dahlfors A. (2007). Adv. Mater..

[cit14] Someya T., Bao Z., Malliaras G. G. (2016). Nature.

[cit15] Simon D. T., Gabrielsson E. O., Tybrandt K., Berggren M. (2016). Chem. Rev..

[cit16] Tang M. L., Bao Z. (2011). Chem. Mater..

[cit17] Lakshminarayana A. N., Ong A., Chi C. (2018). J. Mater. Chem. C.

[cit18] Kaur I., Jia W., Kopreski R. P., Selvarasah S., Dokmeci M. R., Pramanik C., McGruer N. E., Miller G. P. (2008). J. Am. Chem. Soc..

[cit19] Kaur I., Stein N. N., Kopreski R. P., Miller G. P. (2009). J. Am. Chem. Soc..

[cit20] Kaur I., Jazdzyk M., Stein N. N., Prusevich P., Miller G. P. (2010). J. Am. Chem. Soc..

[cit21] Lobanova Griffith O., Gruhn N. E., Anthony J. E., Purushothaman B., Lichtenberger D. L. (2008). J. Phys. Chem. C.

[cit22] Müller M., Ahrens L., Brosius V., Freudenberg J., Bunz U. H. F. (2019). J. Mater. Chem. C.

[cit23] Anthony J. E. (2006). Chem. Rev..

[cit24] Tozawa H., Kitamura K., Hamura T. (2017). Chem. Lett..

[cit25] Hamura T. , in Middle Molecular Strategy: Flow Synthesis to Functional Molecules, ed. K. Fukase and T. Doi, Springer, Singapore, 2021, pp. 203–223

[cit26] Mallouli A., Lepage Y. (1980). Synthesis.

[cit27] Friedman L. (1963). Org. Synth..

[cit28] Seo U. R., Chung Y. K., Lee C. (2016). ChemCatChem.

[cit29] Lin C.-H., Lin K.-H., Pal B., Tsou L.-D. (2009). Chem. Commun..

[cit30] Wang C., Yue C., Smith A., Mack J. (2022). J. Organomet. Chem..

[cit31] Frisch M. J. , Trucks G. W., Schlegel H. B., Scuseria G. E., Robb M. A., Cheeseman J. R., Scalmani G., Barone V., Mennucci B., Petersson G. A., Nakatsuji H., Caricato M., Li X., Hratchian H. P., Izmaylov A. F., Bloino J., Zheng G., Sonnenberg J. L., Hada M., Ehara M., Toyota K., Fukuda R., Hasegawa J., Ishida M., Nakajima T., Honda Y., Kitao O., Nakai H., Vreven T., Montgomery Jr J. A., Peralta J. E., Ogliaro F., Bearpark M., Heyd J. J., Brothers E., Kudin K. N., Staroverov V. N., Keith T., Kobayashi R., Normand J., Raghavachari K., Rendell A., Burant J. C., Iyengar S. S., Tomasi J., Cossi M., Rega N., Millam J. M., Klene M., Knox J. E., Cross J. B., Bakken V., Adamo C., Jaramillo J., Gomperts R., Stratmann R. E., Yazyev O., Austin A. J., Cammi R., Pomelli C., Ochterski J. W., Martin R. L., Morokuma K., Zakrzewski V. G., Voth G. A., Salvador P., Dannenberg J. J., Dapprich S., Daniels A. D., Farkas O., Foresman J. B., Ortiz J. V., Cioslowski J., and Fox D. J., Gaussian 09 (Revision E.01), Gaussian, Inc., Wallingford, CT, 2013

[cit32] Dong H., Wang C., Hu W. (2010). Chem. Commun..

[cit33] Matsuoka S., Jung S., Miyakawa K., Chuda Y., Sugimoto R., Hamura T. (2018). Chem.–Eur. J..

[cit34] Liu Q., Miller G. P. (2024). Beilstein J. Org. Chem..

[cit35] Kitamura K., Kudo R., Sugiyama H., Uekusa H., Hamura T. (2020). Chem. Commun..

[cit36] Sivasakthikumaran R., Rafiq S. M., Sankar E., Clement J. A., Mohanakrishnan A. K. (2015). Eur. J. Org. Chem..

[cit37] Herold B. J., Correia A. F. N., Veiga J. D. S. (1965). J. Am. Chem. Soc..

[cit38] Van Der Drift E., Dammers A. J., Smidt J., Plato M., Möbius K. (1980). J. Magn. Reson..

[cit39] Yang J. , MS thesis, University of New Hampshire, 2015

[cit40] Kodama T., Kawashima Y., Uchida K., Deng Z., Tobisu M. (2021). J. Org. Chem..

[cit41] Meuche D., Strauss H., Heibronner E. (1958). Helv. Chim. Acta.

[cit42] Thiele J., Schneider J. (1909). Adv. Cycloaddit..

[cit43] Kudoh M., Satoh T., Ikeda H., Nakazawa T., Miyashi T., Katagiri S., Sudoh S. (2009). Bull. Chem. Soc. Jpn..

[cit44] Ried W., Schwenecke H. J. (1958). Chem. Ber..

[cit45] Crossley D. L., Gabbutt C. D., Mark Heron B., Kay P., Mogstad M. (2012). Dyes Pigm..

[cit46] Gill S. H., Harmon K. M. (2000). J. Mol. Struct..

