# Supplementary material for: Syntheses, characterizations and reactions of acene-2,3-dicarbaldehydes
Source: RSC Adv. 2024 Aug 9;14(34):25008–18. doi: 10.1039/d4ra04273e (PMC11310926; doi:10.1039/d4ra04273e)
Supplement: RA-014-D4RA04273E-s001 [file RA-014-D4RA04273E-s001.pdf]

Supplementary Information (SI) for RSC Advances.
This journal is © The Royal Society of Chemistry 2024

Syntheses, Characterizations and Reactions of Acene-2,3-Dicarbaldehydes

Supporting Information

Qian Liu and Glen P. Miller*

Department of Chemistry, University of New Hampshire, 23 Academic Way, Durham, New Hampshire 03864-3598 USA

Table of Contents

1. Materials and Methods 1
2. Syntheses of acene-2,3-dicarbaldehydes 3
3. Grignard reactions for the syntheses of diaryl diols 6
4. One-pot, double aldol condensations for the syntheses of acenotropones 9
5. Hydroxytropylium ions formation for NMR characterization..10
6. ^{1}H and ^{13}C NMR Spectra 12
7. HRMS Spectra 45
8. References..50

1. Materials and Methods

Commercial reagents and solvents were purchased from Sigma Aldrich, Alfa Aesar, TCI America or Thermo Fisher Scientific, and used as received. Dry solvents were obtained using a solvent purification system (Innovative Technologies, Inc.) and handled under a nitrogen atmosphere, unless otherwise noted. Flash chromatography was performed using SiliaFlash® F60 40-63 µm (230-400 mesh) 60 Å silica from Silicycle Inc. and RediSep® Rf Silica Flash Columns (12 g, 24 g or 40 g) on a CombiFlash® Rf 200 instrument (Teledyne Isco, Inc.). Evaporation of solvents was accomplished using an IKA® RV 10 digital rotary evaporator. Baker-flex® silica gel IB2-F thin layer chromatography (TLC) plates were purchased from J.T. Baker. A 4-watt 254 nm lamp (Analtytik Jena Co.) and a modified cardboard box were utilized for detection of TLC spots. Melting points were determined in open capillary tubes using a Mel-Temp apparatus, and are uncorrected. Proton nuclear magnetic resonance (^{1}H NMR) spectra and carbon nuclear

magnetic resonance (^{13}C NMR) spectra were recorded on either a Bruker 500 MHz or Bruker 700 MHz Nuclear Magnetic Resonance Spectrometer using 5 mm NMR tubes with plastic caps. High-resolution mass spectra (HRMS) were obtained on a Thermo Scientific Vanquish UHPLC and Exploris 120 Mass Spectrometer at the University of New Hampshire's University Instrumentation Center using a peak-matching protocol to determine the mass and error range of the molecular ion, and employing electrospray as the ionization technique. UV-vis absorption spectra were measured with a Varian Cary 50 Scan UV-Visible Spectrophotometer or SHIMADZU UV-2450 UV-Visible Spectrophotometer and corrected for background signal with a solvent-filled cuvette. Fluorescence spectra were measured on an FS5 Spectrofluorometer (150 W CW Ozone-free xenon arc lamp) from Edinburgh Instruments.

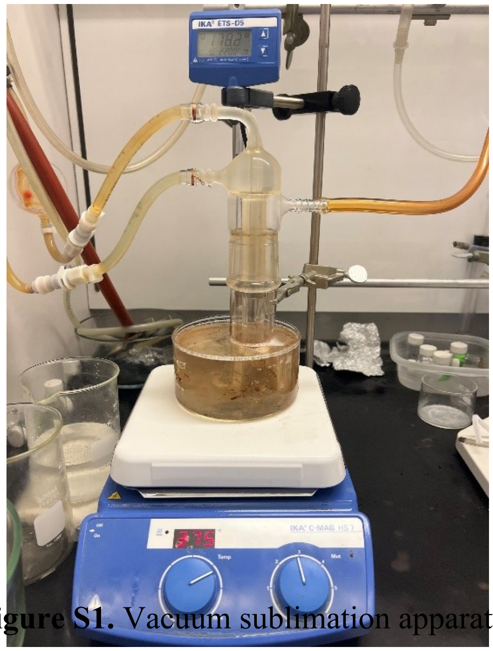

Figure S1. Vacuum sublimation apparatus

2. Syntheses of acene-2,3-dicarbaldehydes

Exp. 1: naphthalene-2,3-dicarbaldehyde (7) and anthracene-2,3-dicarbaldehyde (8):

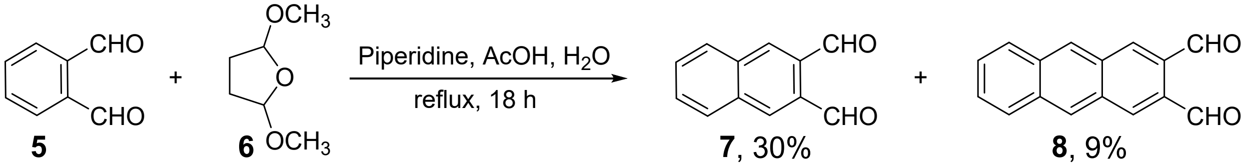

5 **6** **7**, 30% **8**, 9%

A modified literature procedure[1] was utilized. To a mixture of phthalaldehyde (2.0 g, 15 mmol) and 2,5-dimethoxytetrahydrofuran (2.0 g, 15 mmol) was added glacial acetic acid (AcOH) (1.0

mL), H_2O (1.5 mL) and piperidine (2 drops). The resulting mixture was boiled for 18 h during which time the yellow solution turned brown and eventually dark-red. The reaction mixture was allowed to cool to rt and the dark-red precipitate was vacuum filtered. The filtrate suspension was set saved while the filtered solids were washed successively with 3 M HCl, water, methanol and diethyl ether (~10 mL each). The residue was air dried to give a red solid. A ^{1}H NMR spectrum showed that the red solid was a mixture of naphthalene-2,3-dicarbaldehyde, **7**, and anthracene-2,3-dicarbaldehyde, **8** (**7**:**8** = 1.65:1). A vacuum sublimation (132 °C/0.1 Torr/4 hours) was performed to give **7** (0.48 g) as a bright yellow solid. The remaining, red residue was determined to be **8** (0.25 g). The filtrate suspension from above was then vacuum filtered a second time to obtain a dark brown, sticky solid. A ^{1}H NMR spectrum revealed a mixture of **7** and **8**. Vacuum sublimation was performed (132 °C/0.1 Torr/6 hours) again to afford additional **7** (0.34 g), then again at higher temperature (220 °C/0.1 Torr/4 hours) to obtain pure **8** (0.07 g). The combined isolated yield of **7** as a bright yellow solid was 0.82 g (30%, m.p. 119-120 °C, lit.[1] m.p. 132 °C). ^{1}H NMR (700 MHz, $CDCl_3$) δ 10.65 (s, 2H), 8.47 (s, 2H), 8.07 (m, J = 3.3, 6.1 Hz, 2H), 7.75 (m, J = 3.2, 6.2 Hz, 2H). ^{13}C NMR (176 MHz, $CDCl_3$) δ 192.5, 134.5, 134.4, 132.9, 130.0, 129.7. UV–vis λ_{max} (1×10^{-4} M in CH_2Cl_2): 340 nm. HRMS (ESI): 185.0593 [calc'd for M+H$^+$: 185.0603]. The combined isolated yield of **8** was 0.32 g (9%, m.p. 214-216 °C, lit.[1] m.p. 217 °C) as an orange-red solid. ^{1}H NMR (700 MHz, $CDCl_3$) δ 10.66 (s, 2H), 8.65 (s, 2H), 8.63 (s, 2H), 8.13 – 8.09 (m, 2H), 7.67 – 7.63 (m, 2H). ^{13}C NMR (176 MHz, $CDCl_3$) δ 192.5, 136.5, 133.8, 132.1, 130.9, 129.6, 128.7, 127.8. UV–vis λ_{max} (1×10^{-4} M in CH_2Cl_2): 410 nm. HRMS (ESI): 235.0751 [calc'd for M+H$^+$: 235.0759].

Exp. 2: naphthalene-2,3-dicarbaldehyde (7) and anthracene-2,3-dicarbaldehyde (8):

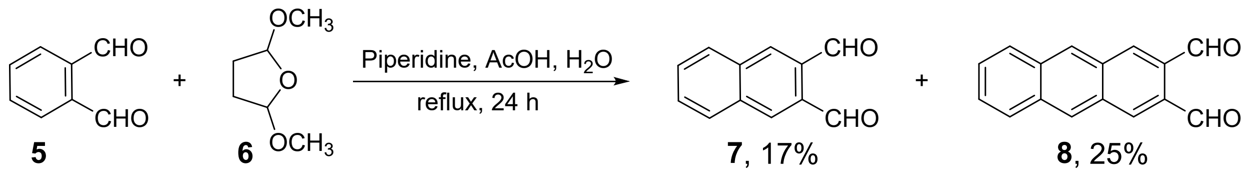

A modified literature procedure[1] was utilized. To a mixture of phthalaldehyde (2.0 g, 15 mmol) and 2,5-dimethoxytetrahydrofuran (4.0 g, 30 mmol) was added glacial acetic acid (AcOH) (1.5 mL), H_2O (1.5 mL) and piperidine (3 drops). The resulting mixture was boiled for ~24 h during which time the yellow solution turned brown and eventually dark-red. Following the same work-up described in Exp. 1 above, the solids collected from the first vacuum filtration were identified by ^{1}H NMR spectroscopy as mixture of naphthalene-2,3-dicarbaldehyde, **7**, and anthracene-2,3-dicarbaldehyde, **8** (**7**:**8** = 1:6.5). As in Exp. 1, the filtrate from the vacuum filtration was vacuum filtered a second time and a ^{1}H NMR spectrum revealed the collected solids to be a mixture of **7** and **8**. Vacuum sublimations were performed as described in Exp. 1. The combined yield of naphthalene-2,3-dicarbaldehyde, **7**, as a bright yellow solid was 0.47 g (17%) and of anthracene-2,3-dicarbaldehyde, **8**, as an orange-red solid was 0.88 g (25%).

Exp. 3: naphthalene-2,3-dicarbaldehyde (7), anthracene-2,3-dicarbaldehyde (8) and tetracene-2,3-dicarbaldehyde (9):

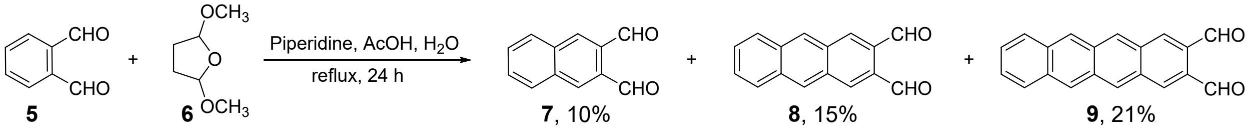

A modified literature procedure[1] was utilized. To a mixture of phthalaldehyde (2.0 g, 15 mmol) and 2,5-dimethoxytetrahydrofuran (7.9 g, 60 mmol) was added glacial acetic acid (AcOH) (3 mL), H_2O (1 mL) and piperidine (4 drops). The resulting mixture was boiled for ~24 h during which time the yellow solution turned brown and eventually dark-red. Following the same work-up described in Exp. 1 above, the solids collected from the first vacuum filtration were identified by ^{1}H NMR spectroscopy to be a mixture of naphthalene-2,3-dicarbaldehyde, **7**, anthracene-2,3-dicarbaldehyde, **8** and tetracene-2,3-dicarbaldehyde, **9** (**7**:**8**:**9** = 0.26:1:0.3). As before, the filtrate suspension was vacuum filtered a second time and identified as a mixture of **7**, **8** and **9**. Successive vacuum sublimations were performed for both the first and second batches of collected solids. At 132 °C/0.1 Torr, pure **7** was collected (0.27 g, 10%) as a bright yellow solid over 6 hours. At 180 °C and 0.1 Torr, pure **8** was collected over 4 hours (0.51 g, 15%) as an orange-red solid. At 220 °C and 0.1 Torr, a mixture of **8** and **9** was collected. Subsequently, this mixture was vacuum sublimed again at 180 °C and 0.1 Torr for an additional 4 hours to deplete the mixture of residual **8**. A final vacuum sublimation (220 °C/0.1 Torr/6 hours) afforded tetracene-2,3-dicarbaldehyde, **9** (0.89 g, 21%, m.p. 286 °C (subl.), lit.[1] m.p. 342 °C (subl.)) as a red solid. **9**: ^{1}H NMR (700 MHz, $CDCl_3$) δ 10.7 (s, 2H), 8.9 (s, 2H), 8.8 (s, 2H), 8.6 (s, 2H), 8.1 (m, *J* = 3.3, 6.5 Hz, 2H), 7.5 (m, *J* = 3.3, 6.7 Hz, 2H). ^{13}C NMR (176 MHz, $CDCl_3$) δ 192.4, 137.5, 132.8, 131.8, 131.4, 130.6, 130.1, 128.5, 127.6, 126.7. UV-Vis λ_{max} (1×10^{-4} M in CH_2Cl_2): 503 nm. HRMS (ESI): 285.0905; 307.0725 [calc'd for $M+H^+$: 285.0916; $M+Na^+$: 307.0735].

Exp. 4: naphthalene-2,3-dicarbaldehyde (7), anthracene-2,3-dicarbaldehyde (8) and tetracene-2,3-dicarbaldehyde (9):

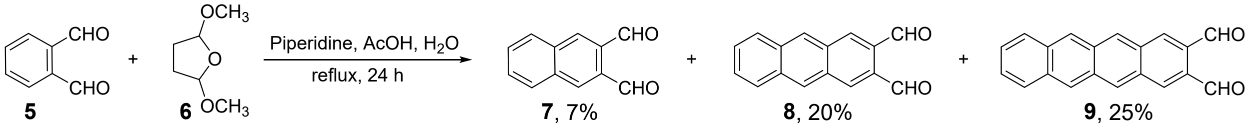

A modified literature procedure[1] was utilized. To a mixture of phthalaldehyde (2.0 g, 15 mmol) and 2,5-dimethoxytetrahydrofuran (11.9 g, 90 mmol) was added glacial acetic acid (AcOH) (4 mL), H_2O (1 mL) and piperidine (5 drops). The resulting mixture was boiled for ~24 h during which time the yellow solution turned brown and eventually dark-red. After work-up as described in Exp. 1 above, a ^{1}H NMR spectrum of the solids collected from the first vacuum filtration were shown to be a mixture of **7**, **8** and **9** (**7**:**8**:**9** = 0.2:1:1.2). Successive vacuum sublimations were performed on both the first and second batches of solids collected from successive vacuum

filtrations, as before in Exps. 1, 2 and 3. At 132 °C and 0.1 Torr, pure **7** was collected over 6 hours (0.20 g, 7%) as a bright yellow solid. At 180 °C and 0.1 Torr, pure **8** was collected over 4 hours (0.71 g, 20%) as an orange-red solid. Lastly, at 220 °C and 0.1 Torr, pure **9** was collected over 6 hours (1.06 g, 25%) as a red solid.

Exp. 5: tetracene-2,3-dicarbaldehyde (9):

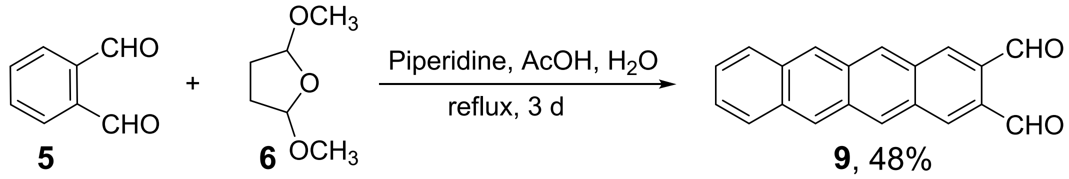

5 **6** **9**, 48%

A modified literature procedure[1] was utilized. To a mixture of phthalaldehyde (2.0 g, 15 mmol) and 2,5-dimethoxytetrahydrofuran (15.9 g, 120 mmol) was added glacial acetic acid (AcOH) (4 mL), H_2O (1 mL) and piperidine (5 drops). The resulting mixture was boiled for 72 hours during which time the yellow solution turned brown and eventually dark red. After work-up as described above, the first and second batches of solids collected by vacuum filtration were vacuum sublimed at 220 °C and 0.1 Torr for 12 hours to afford **9** (2.03 g, 48%) as a red solid.

3. Grignard reactions for the syntheses of diaryl diols

1,2-phenylenebis(mesitylmethanol) (11) and (2-(hydroxy(mesityl)methyl)phenyl)(mesityl)methanone (15):

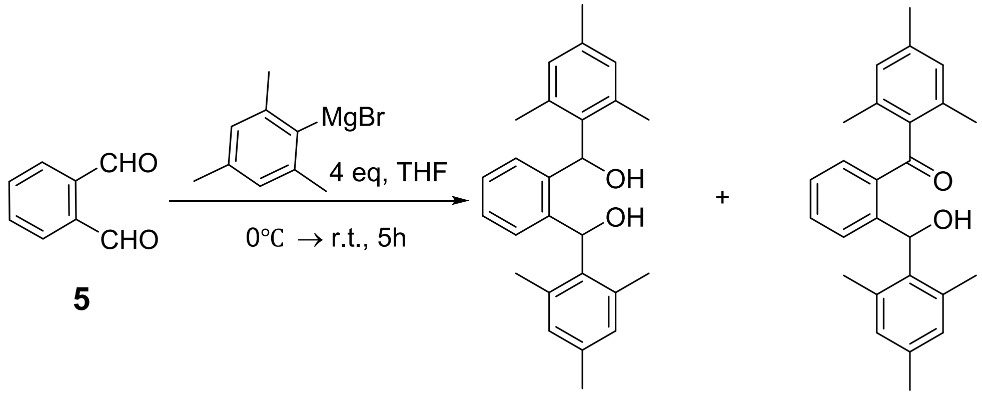

5 **11** **15**

To a solution of *o*-phthalaldehyde, **5** (0.5 g, 5 mmol) in THF (5 mL) was added mesityl magnesium bromide (prepared from 2-bromomesitylene (2.3 mL, 14.91 mmol) and magnesium turnings (0.36

g, 14.91 mmol) in 10 mL THF as a brown, yellow solution) in an ice-bath (0 °C). The reaction mixture was stirred for 5 h at room temperature. Then the reaction was quenched with 10 mL of a saturated aqueous solution of NH_4Cl and then extracted with 2×20 mL ethyl acetate. The extract was washed with 20 mL of brine solution and then dried over anhydrous Na_2SO_4. The solvent was rotary evaporated at reduced pressure to give an off-white crude product. The crude product was purified by silica gel CombiFlash chromatography (hexane/ethyl acetate = 85/15) to obtain two white solids: **11** (0.75 g, 54%). M.p. 180-181 °C. ^1H NMR (500 MHz, $CDCl_3$) δ 7.12 (m, 2H), 7.01 (m, 2H), 6.88 (s, 4H), 6.55 (s, 2H), 3.65 (s, 2H), 2.28 (s, 6H), 2.25 (s, 12H). ^{13}C NMR (126 MHz, $CDCl_3$) δ 140.1, 137.0, 136.9, 134.6, 130.2, 128.3, 127.9, 71.1, 21.2, 20.8. HRMS (ESI): 237.1271; 357.2208 [calc'd for $M+H^+$-Mes-H_2O: 237.1279; $M+H^+$-H_2O: 357.2218]. **15** (0.11 g, 8%). M.p. 208-209 °C, lit.[2] m.p. 220-221 °C. ^1H NMR (500 MHz, $CDCl_3$) δ 7.47 (m, 1H), 7.38 (m, 1H), 7.26 – 7.20 (m, 1H), 7.10 (m, 1H), 6.90 (s, 4H), 6.61 (d, 1H), 5.16 (d, 1H), 2.34 (s, 3H), 2.31 (s, 3H), 2.29 (s, 12H). ^{13}C NMR (126 MHz, $CDCl_3$) δ 205.2, 144.3, 139.1, 137.8, 137.2, 137.2, 136.7, 134.2, 133.6, 133.3, 130.0, 129.0, 129.0, 128.7, 127.7, 70.5, 21.5, 21.2, 20.9, 19.6. HRMS (ESI): 355.2049 [calc'd for $M+H^+$-H_2O: 355.2062].

naphthalene-2,3-diylbis(mesitylmethanol) (12):

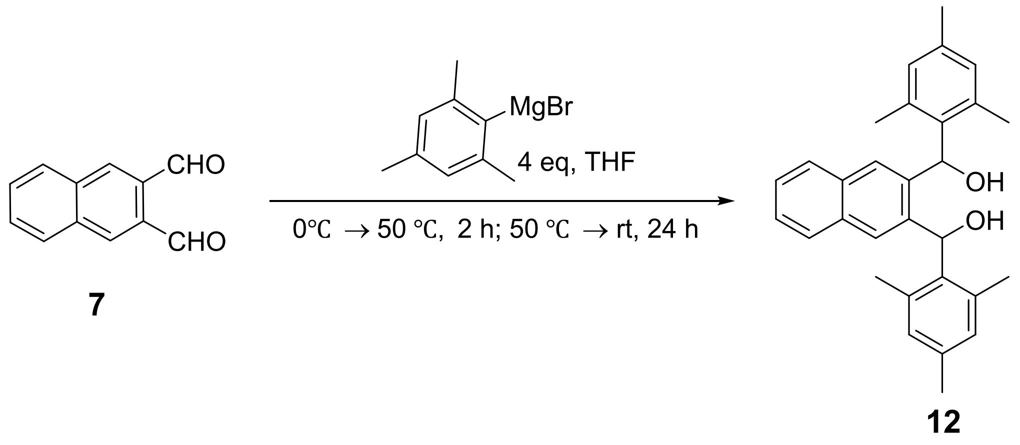

To a solution of naphthalene-2,3-dicarbaldehyde, **7** (0.10 g, 0.54 mmol), in THF (5 mL) was added mesityl magnesium bromide (2.2 mL, 1 M in THF) [prepared from 2-bromomesitylene (3.1 mL, 20 mmol) and magnesium turnings (0.49 g, 20 mmol) in 17 mL THF as a yellowish-brown solution] in an ice-bath (0 °C). The reaction mixture was stirred for 2 h at 50 °C, then cooled to rt and continued to stir for ~24 h. The reaction was quenched with 10 mL of a saturated aqueous solution of NH_4Cl and then extracted with 2×20 mL ethyl acetate. The extract was washed with 20 mL of brine solution and then dried over anhydrous Na_2SO_4. The solvent was rotary evaporated at reduced pressure to give a light-yellow oil. The oil was placed under vacuum for 12 h during

which time it solidified. The solid was recrystallized from hexane (5 mL), vacuum filtered and then washed with 2 mL cold hexane to give **12** as an off-white solid (0.17 g, 74%). M.p. 196-198 °C. ^{1}H NMR (700 MHz, $CDCl_3$) δ 7.64 (m, 2H), 7.50 (s, 2H), 7.37 (m, 2H), 6.92 (s, 4H), 6.67 (s, 2H), 3.62 (s, 2H), 2.32 (s, 6H), 2.27 (s, 12H). ^{13}C NMR (176 MHz, $CDCl_3$) δ 137.8, 137.1, 137.0, 134.7, 132.6, 130.3, 127.9, 126.1, 71.3, 21.2, 20.9. HRMS (ESI): 287.1428; 407.2366 [calc'd for $M+H^+$-Mes-H_2O: 287.1436; $M+H^+$-H_2O: 407.2375].

anthracene-2,3-diylbis(mesitylmethanol) (13):

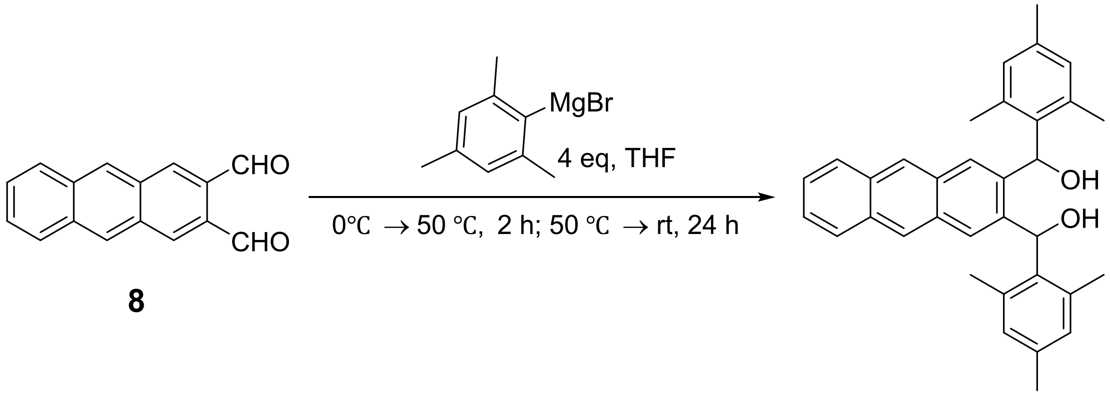

8 **13**

To a solution of anthracene-2,3-dicarbaldehyde, **8** (0.10 g, 0.43 mmol), in THF (5 mL) was added mesityl magnesium bromide (1.7 mL, 1 M in THF) [prepared from 2-bromomesitylene (3.1 mL, 20 mmol) and magnesium turnings (0.49 g, 20 mmol) in 17 mL THF as a yellowish-brown solution] in an ice-bath (0 °C). The reaction mixture was stirred for 2 h at 50 °C, then cooled to rt and stir for ~24 h. The reaction was quenched with 10 mL of a saturated aqueous solution of NH_4Cl and then extracted with 2×20 mL ethyl acetate. The extract was washed with 20 mL of brine solution and then dried over anhydrous Na_2SO_4. The solvent was rotary evaporated at reduced pressure to give a yellow oil. The oil was placed under vacuum for ~12h during which time it solidified. The solid was recrystallized from hexane (10 mL), vacuum filtered and washed with 5 mL cold hexane to give **13** as a yellow solid (0.15 g, 73%). M.p. 180-182 °C. ^{1}H NMR (700 MHz, $CDCl_3$) δ 8.23 (s, 2H), 7.88 (m, 2H), 7.66 (s, 2H), 7.39 (m, 2H), 6.93 (s, 4H), 6.68 (s, 2H), 3.72 (s, 2H), 2.33 (s, 6H), 2.29 (s, 12H). ^{13}C NMR (176 MHz, $CDCl_3$) δ 137.4, 137.1, 137.0, 134.7, 131.9, 130.8, 130.3, 128.1, 128.0, 126.3, 125.4, 71.3, 21.2, 20.9. HRMS (ESI): 337.1583; 457.2521 [calc'd for $M+H^+$-Mes-H_2O: 337.1592; $M+H^+$-H_2O: 457.2531].

tetracene-2,3-diylbis(mesitylmethanol) (14):

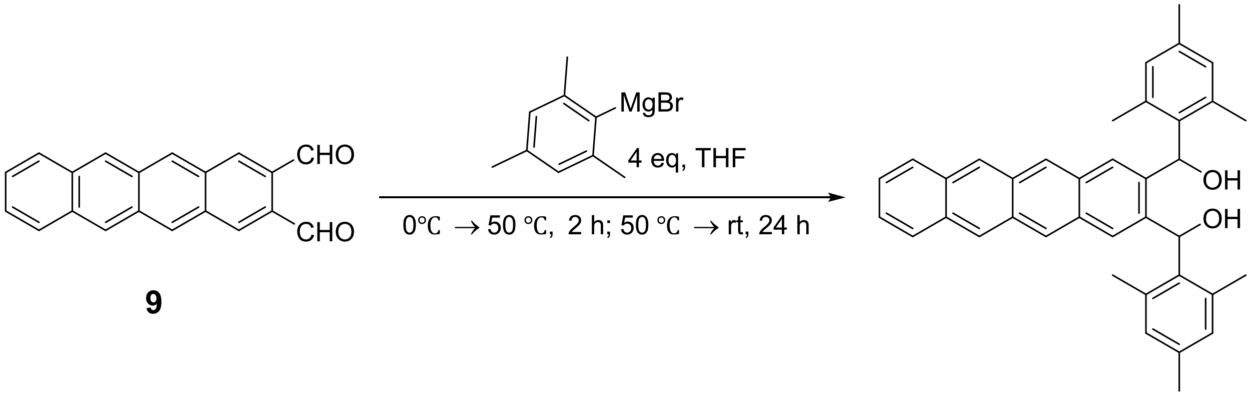

9

14

To a solution of tetracene-2,3-dicarbaldehyde, **9** (0.10 g, 0.35 mmol), in THF (5 mL) was added mesityl magnesium bromide (1.4 mL, 1 M in THF) [prepared from 2-bromomesitylene (3.1 mL, 20 mmol) and magnesium turnings (0.49 g, 20 mmol) in 17 mL THF as a yellowish-brown solution] in an ice-bath (0 °C). The reaction mixture was stirred for 2 h at 50 °C, then cooled to rt and continued to stir for ~24 h. The reaction was quenched with 10 mL of a saturated aqueous solution of NH_4Cl and then extracted with 2×20 mL ethyl acetate. The extract was washed with 20 mL of brine solution and then dried over anhydrous Na_2SO_4. The solvent was rotary evaporated at reduced pressure to give a brown solid. The solid was recrystallized from hexane (10 mL), vacuum filtered and washed with 5 mL of cold hexane to give a tan solid (0.13 g, 71%). M.p. 220-222 °C. ^{1}H NMR (700 MHz, $CDCl_3$) δ 8.53 (s, 2H), 8.46 (s, 2H), 7.94 (m, 2H), 7.67 (s, 2H), 7.36 (m, 2H), 6.95 (s, 4H), 6.70 (s, 2H), 3.62 (s, 2H), 2.35 (s, 6H), 2.33 (s, 12H). ^{13}C NMR (176 MHz, $CDCl_3$) δ 137.2, 137.1, 137.1, 134.7, 131.5, 130.6, 130.44, 130.3, 128.3, 128.2, 126.5, 126.3, 125.2, 71.4, 21.3, 20.9. HRMS (ESI): 387.1740; 507.2677; 524.2706 [calc'd for M+H$^+$-Mes-H_2O: 387.1749; M+H$^+$-H_2O: 507.2688; M$^+$: 524.2715].

4. One-pot, double aldol condensations for the syntheses of acenotropones

6,8-diphenyl-7*H*-benzo[7]annulen-7-one (16):

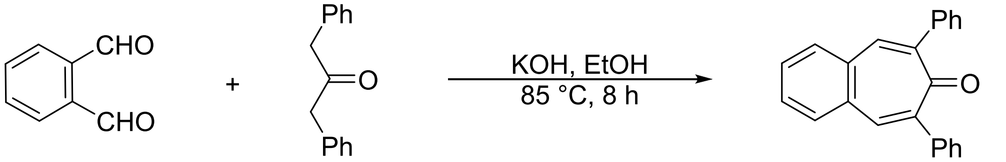

5 **16**

To a solution of *o*-phthalaldehyde, **5** (0.50 g, 3.72 mmol), and 1,3-diphenylacetone (0.78 g, 3.72 mmol) in ethanol (20 mL) was added powdery KOH (0.42 g, 7.46 mmol). With the addition of KOH, the reaction mixture color changed from light yellow to orange, and then to bright yellow. The reaction mixture was then heated to 85 °C for 8 h. The resulting light yellowish precipitate

was cooled in an ice bath, vacuum filtered, washed with cold ethanol and air dried to obtain a pale yellowish solid, **16** (0.92 g, 80%). M.p. 117-118 °C, lit.[3] m.p. 117-119 °C. ^{1}H NMR (700 MHz, $CDCl_3$) δ 7.75 – 7.70 (m, 4H), 7.60 – 7.54 (m, 6H), 7.44 – 7.35 (m, 6H). ^{13}C NMR (176 MHz, $CDCl_3$) δ 187.3, 145.8, 140.8, 139.5, 134.7, 133.5, 129.7, 129.3, 128.1, 127.8. UV–vis λ_{max} (1×10^{-5} M in CH_2Cl_2): 284 nm.

7,9-diphenyl-8*H*-cyclohepta[*b*]naphthalen-8-one (17):

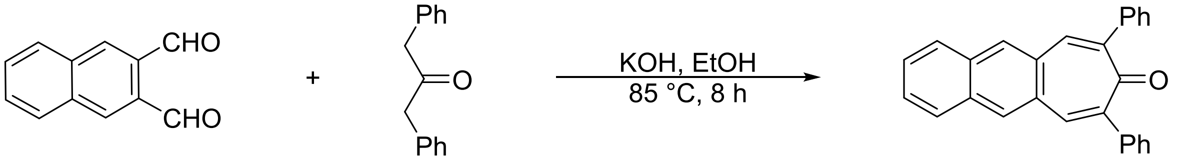

7 **17**

To a solution of naphthalene-2,3-dicarbaldehyde, **7** (0.10 g, 0.54 mmol), and 1,3-diphenylacetone (0.11 g, 0.54 mmol) in ethanol (10 mL) was added powdery KOH (0.06 g, 1.08 mmol). With the addition of KOH, the reaction mixture color changed from yellow to brown, then to yellow. The reaction mixture was then heated to 85 °C for 8 h. The resulting yellow precipitate was cooled in an ice bath, vacuum filtered, washed with cold ethanol and air dried to obtain a yellow solid, **17** (0.15 g, 77%). M.p. 189-190 °C, lit.[4] m.p. 196-197 °C. ^{1}H NMR (700 MHz, $CDCl_3$) δ 8.20 (s, 2H), 7.96 (m, 2H), 7.84 (s, 2H), 7.61 (m, 2H), 7.58 (d, 4H), 7.43 (t, 4H), 7.38 (t, 2H). ^{13}C NMR (176 MHz, $CDCl_3$) δ 188.0, 144.2, 141.0, 140.0, 133.7, 133.2, 132.1, 129.3, 128.0, 128.0, 127.9, 127.7. UV–vis λ_{max} (1×10^{-5} M in CH_2Cl_2): 306 nm. HRMS (ESI): 359.1429 [calc'd for $M+H^+$: 359.1436].

8,10-diphenyl-9*H*-cyclohepta[*b*]anthracen-9-one (18):

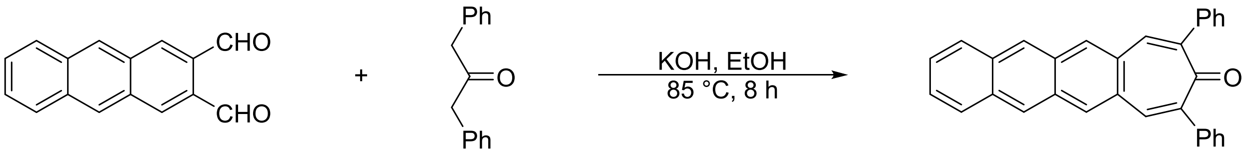

8 **18**

To a solution of anthracene-2,3-dicarbaldehyde, **8** (100 mg, 0.43 mmol), and 1,3-diphenylacetone (90 mg, 0.43 mmol) in ethanol (10 mL) was added powdery KOH (48 mg, 0.85 mmol). With the addition of KOH, the reaction mixture color changed from orange red to red. The reaction mixture was then heated to 85 °C for 8 h. The resulting dark red precipitate was cooled in an ice bath, vacuum filtered, washed with cold ethanol and air dried to obtain an orange solid, **18** (110 mg, 63%). m.p. 196 °C (subl.). ^{1}H NMR (700 MHz, $CDCl_3$) δ 8.54 (s, 2H), 8.36 (s, 2H), 8.05 (m, 2H), 7.83 (s, 2H), 7.57 (m, 4H), 7.54 (m, 2H), 7.43 (t, 4H), 7.39 – 7.35 (m, 2H). ^{13}C NMR (176 MHz, CDCl3) δ 188.4, 143.4, 141.1, 140.1, 134.4, 132.8, 131.5, 130.7, 129.3, 128.3, 128.0, 127.6, 126.9,

126.5. UV–vis λ_{max} (1×10^{-5} M in CH_2Cl_2): 332 nm. HRMS (ESI): 409.1583 [calc'd for $M+H^+$: 409.1592].

9,11-diphenyl-10*H*-cyclohepta[*b*]tetracen-10-one (19):

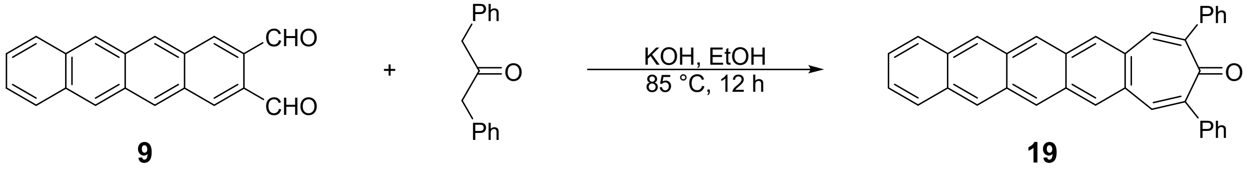

To a solution of tetracene-2,3-dicarbaldehyde, **9** (50 mg, 0.18 mmol), and 1,3-diphenylacetone (37 mg, 0.18 mmol) in ethanol (10 mL) was added powdery KOH (20 mg, 0.35 mmol). The reaction mixture was then heated to 85 °C for 12 h during which time the reaction mixture color changed from red to dark-red. The resulting dark-red precipitate was cooled in an ice bath, vacuum filtered, washed with cold ethanol and air dried to obtain a red solid, **19** (44 mg, 53%). Due to the instability of **19** in solution phase, we did not obtain a ^{13}C NMR spectrum. ^1H NMR (700 MHz, $CDCl_3$) δ 8.78 (s, 2H), 8.71 (s, 2H), 8.34 (s, 2H), 8.03 – 7.99 (m, 2H), 7.79 (s, 2H), 7.58 – 7.54 (m, 4H), 7.47 – 7.41 (m, 6H), 7.40 – 7.35 (m, 2H). HRMS (ESI): 459.1740 [calc'd for $M+H^+$: 459.1749].

5. Hydroxytropylium ions formation for NMR characterization

tropone to hydroxytropylium ion:

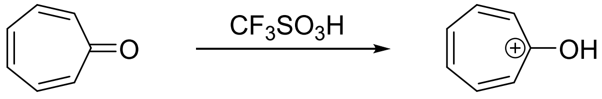

To a solution of commercially available tropone (13 mg, 0.12 mmol) in $CDCl_3$ (2 mL) was added 10 drops of triflic acid (CF_3SO_3H) with shaking to form the hydroxytropylium ion as a yellow solution. **Tropone** NMR: ^1H NMR (700 MHz, $CDCl_3$) δ 7.09 (m, 2H), 7.03 – 6.99 (m, 2H), 6.97 – 6.93 (m, 2H). ^{13}C NMR (176 MHz, $CDCl_3$) δ 188.2, 142.2, 136.1, 134.7. **Hydroxytropylium ion** NMR: ^1H NMR (700 MHz, $CDCl_3$) δ 8.19 – 8.13 (m, 2H), 8.01 (m, 2H), 7.78 (d, 2H). ^{13}C NMR (176 MHz, $CDCl_3$) δ 181.0, 151.9, 147.8, 140.0.

anthracenotropone (18) to hydroxyanthracenotropylium ion (22):

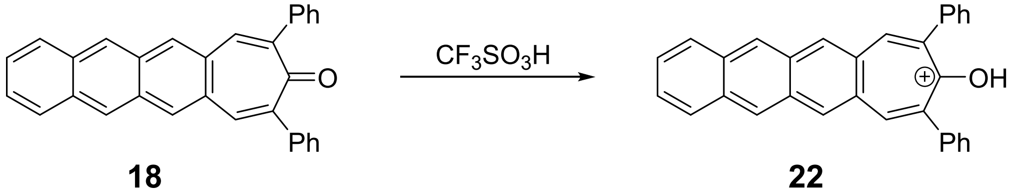

To a solution of anthracenotropone (7 mg, 0.017 mmol) in $CDCl_3$ (2 mL) was added 5 drops of triflic acid (CF_3SO_3H) with shaking to form the hydroxyanthracenotropylium ion, **22**, as a green solution. ^1H NMR (700 MHz, $CDCl_3$) δ 9.33 (s, 2H), 9.26 (s, 2H), 9.11 (s, 2H), 8.32 (s, 2H), 7.84

(s, 2H), 7.73 – 7.65
141.9, 136.0, 135.4

6. ^{1}H and ^{13}C N

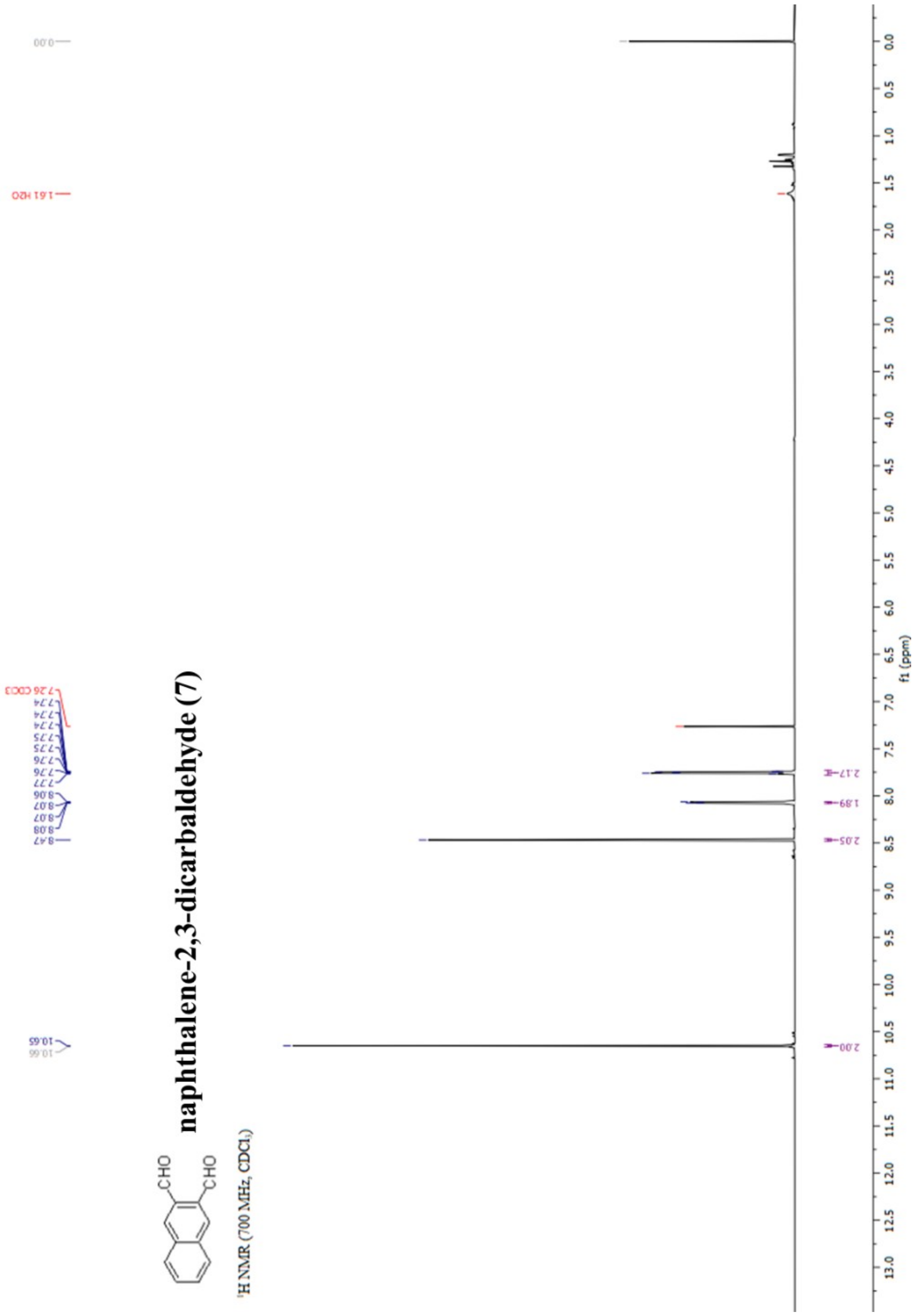

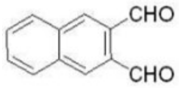

naphthalene-2,3-dicarbaldehyde (7)

^{13}C NMR (176 MHz, $CDCl_3$) δ 192.52, 134.47, 134.45, 132.86, 130.04, 129.67.

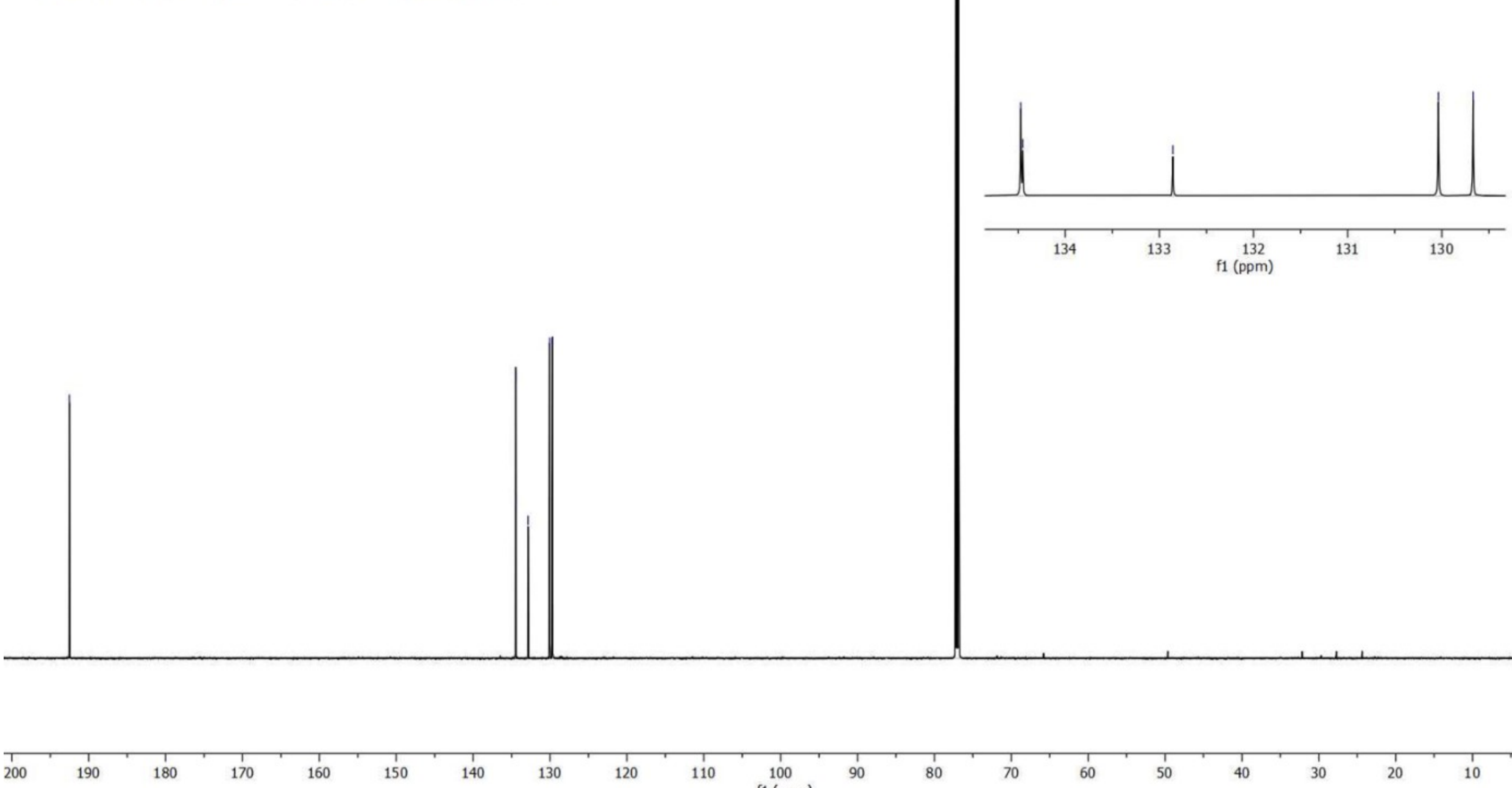

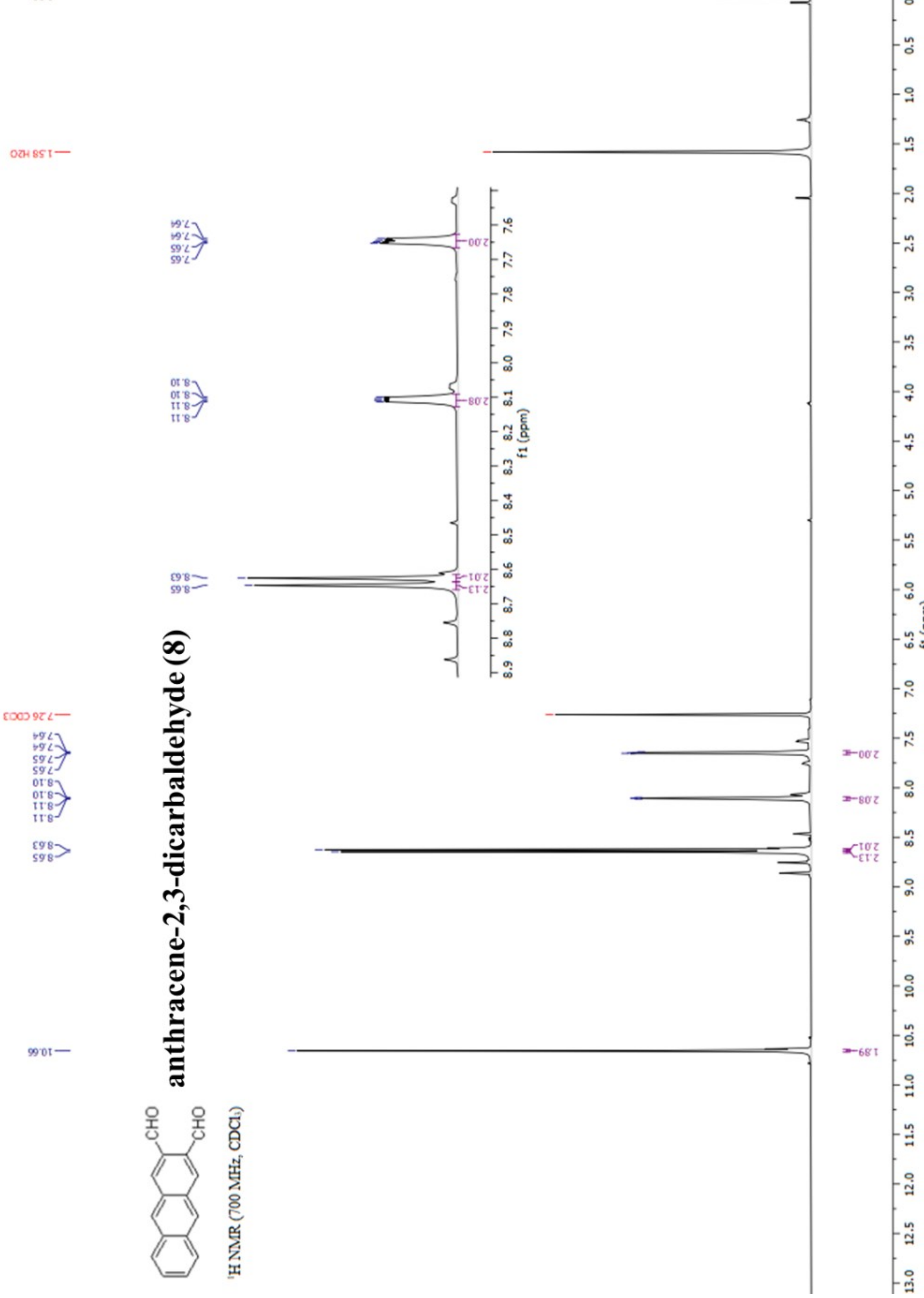

anthracene-2,3-dicarbaldehyde (8)
1H NMR (700 MHz, CDCl3)

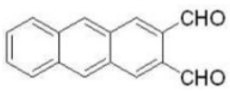

anthracene-2,3-dicarbaldehyde (8)

^{13}C NMR (176 MHz, $CDCl_3$) δ 192.46, 136.45, 133.82, 132.06, 130.93, 129.57, 128.67, 127.79.

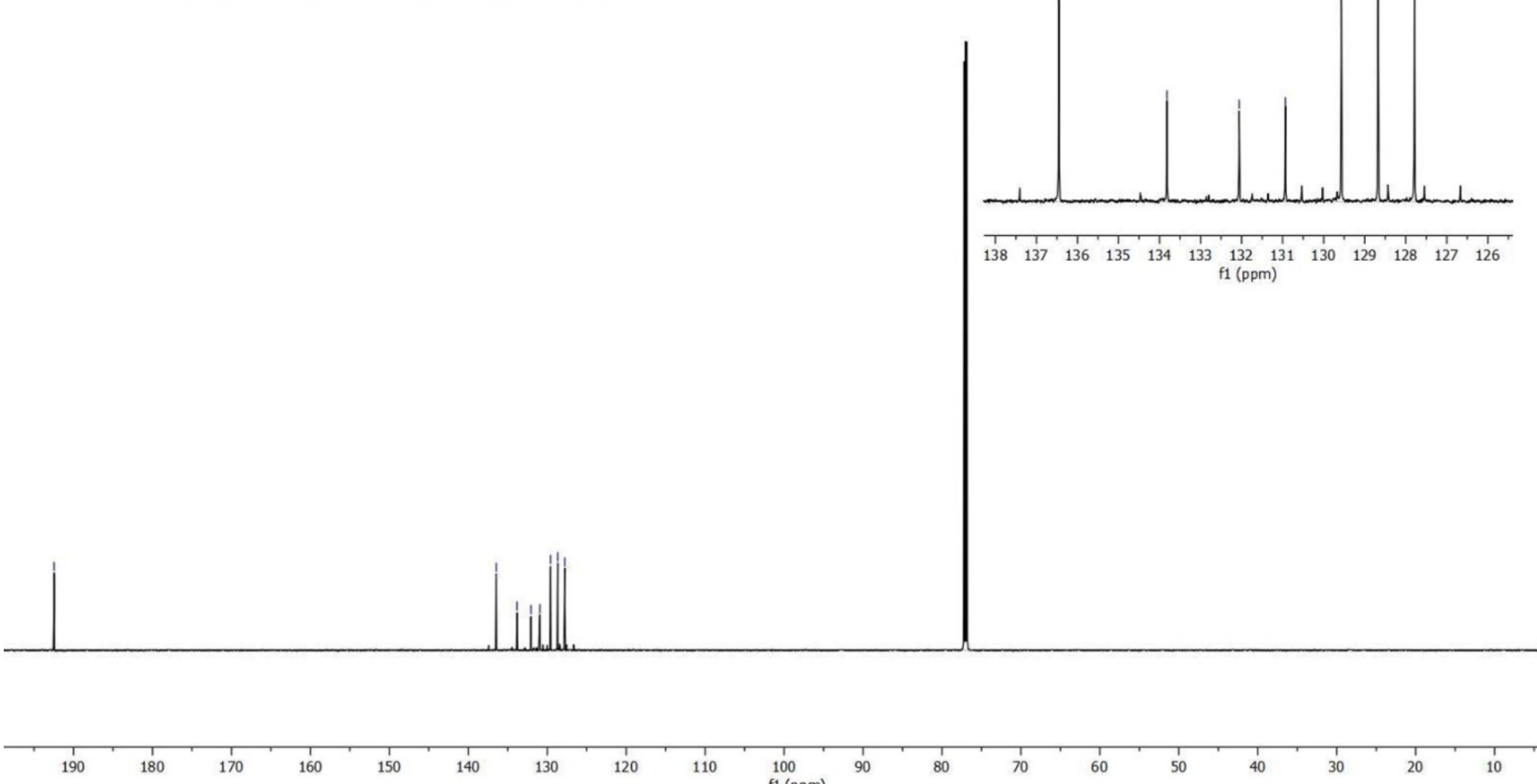

tetracene-2,3-dicarbaldehyde (9)

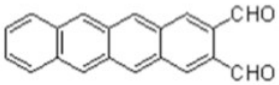

^{1}H NMR (700 MHz, CDCl$_3$)

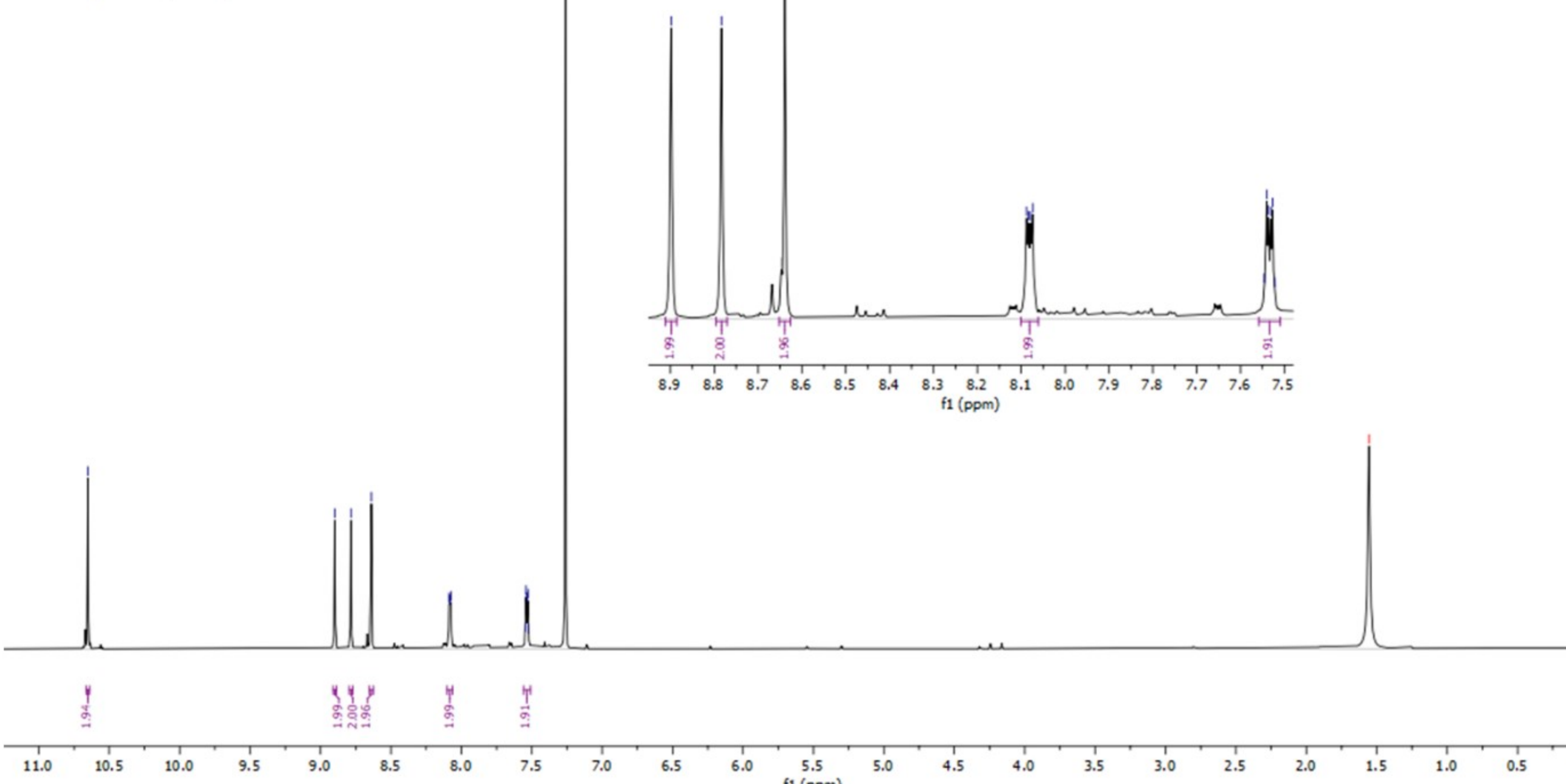

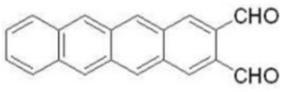

tetracene-2,3-dicarbaldehyde (9)

^{13}C NMR (176 MHz, $CDCl_3$) δ 192.36, 137.45, 132.83, 131.78, 131.40, 130.58, 130.08, 128.45, 127.58, 126.67.

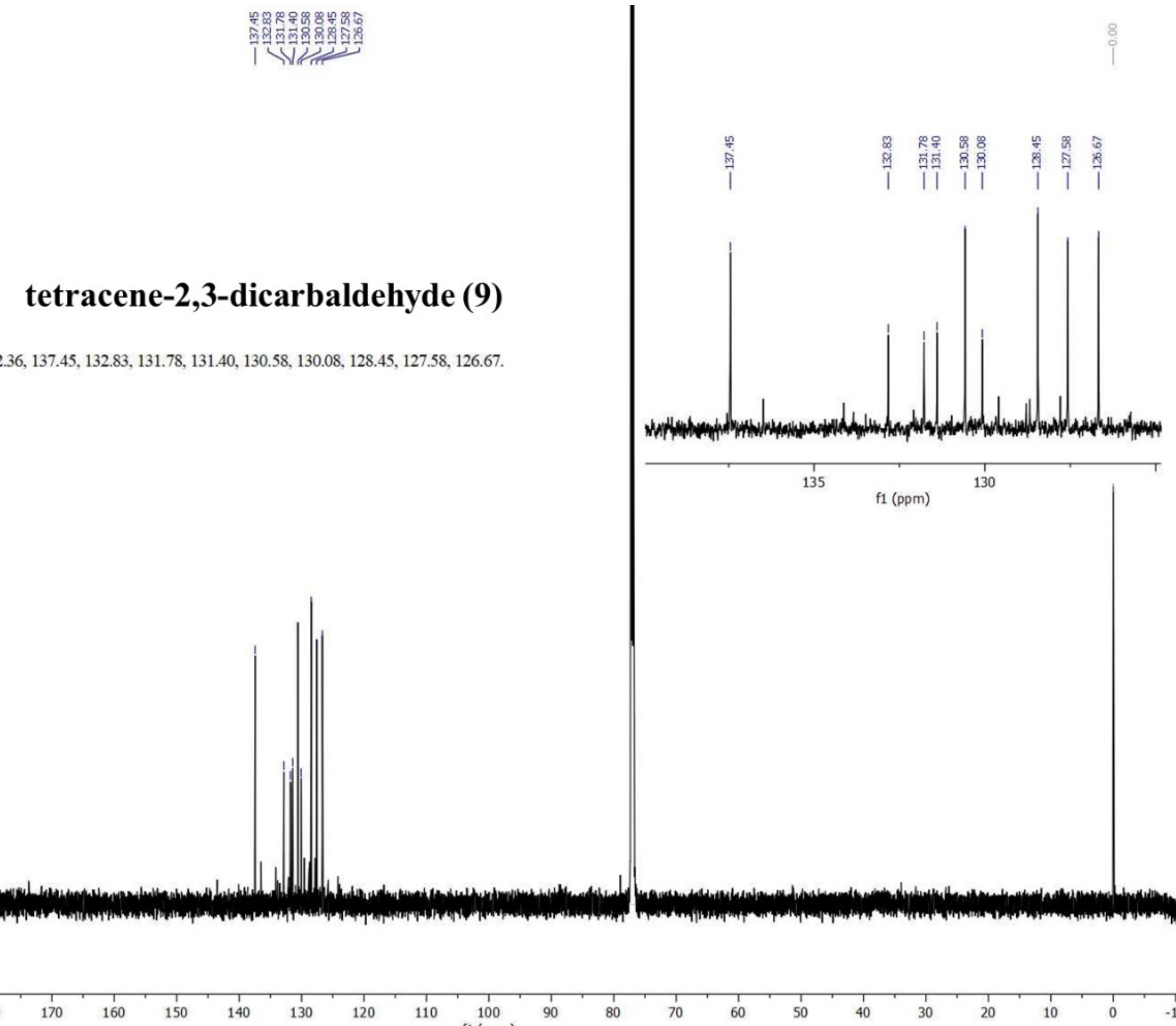

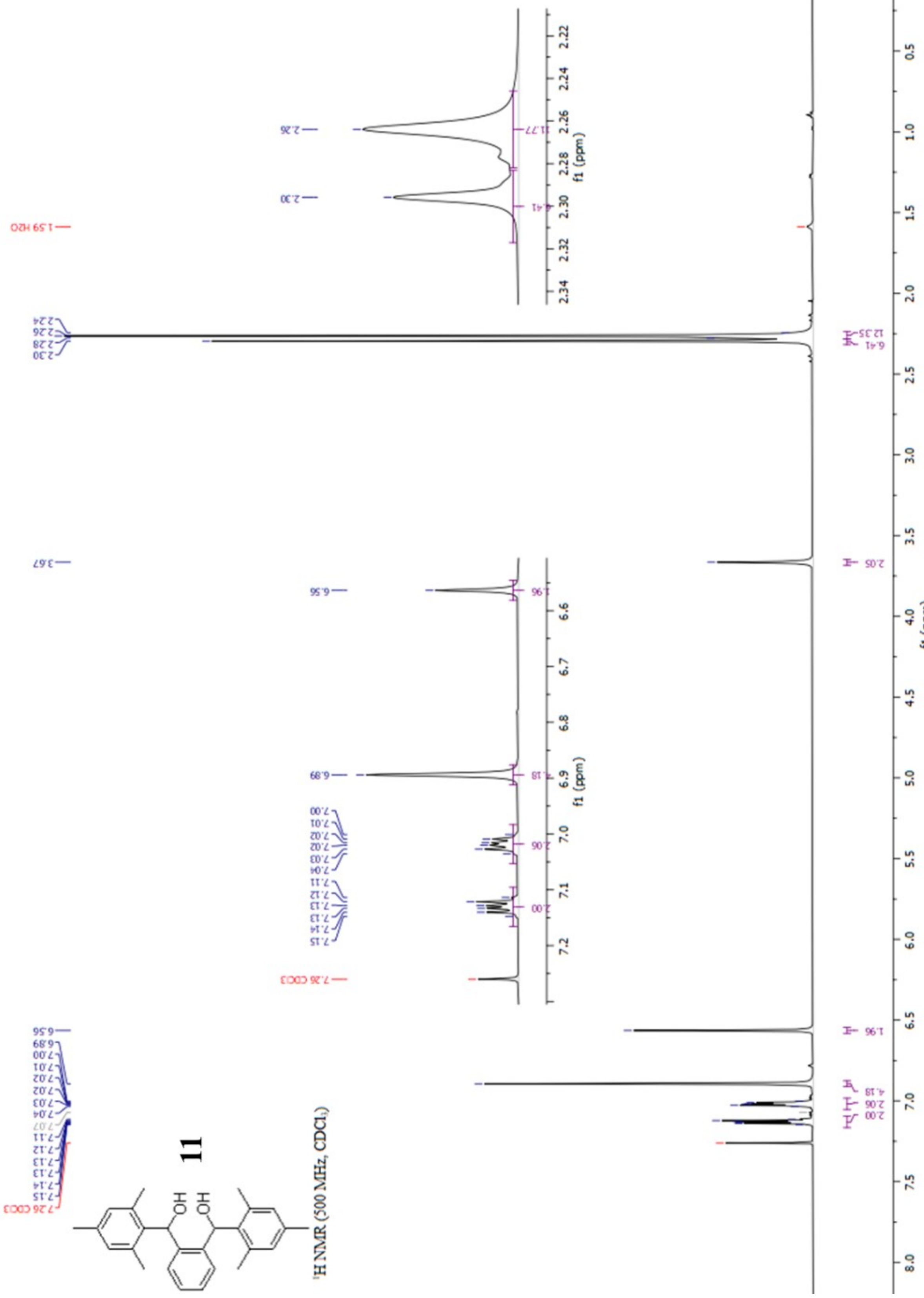

11

^{1}H NMR (500 MHz, $CDCl_3$)

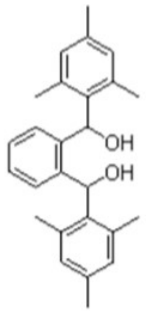

11

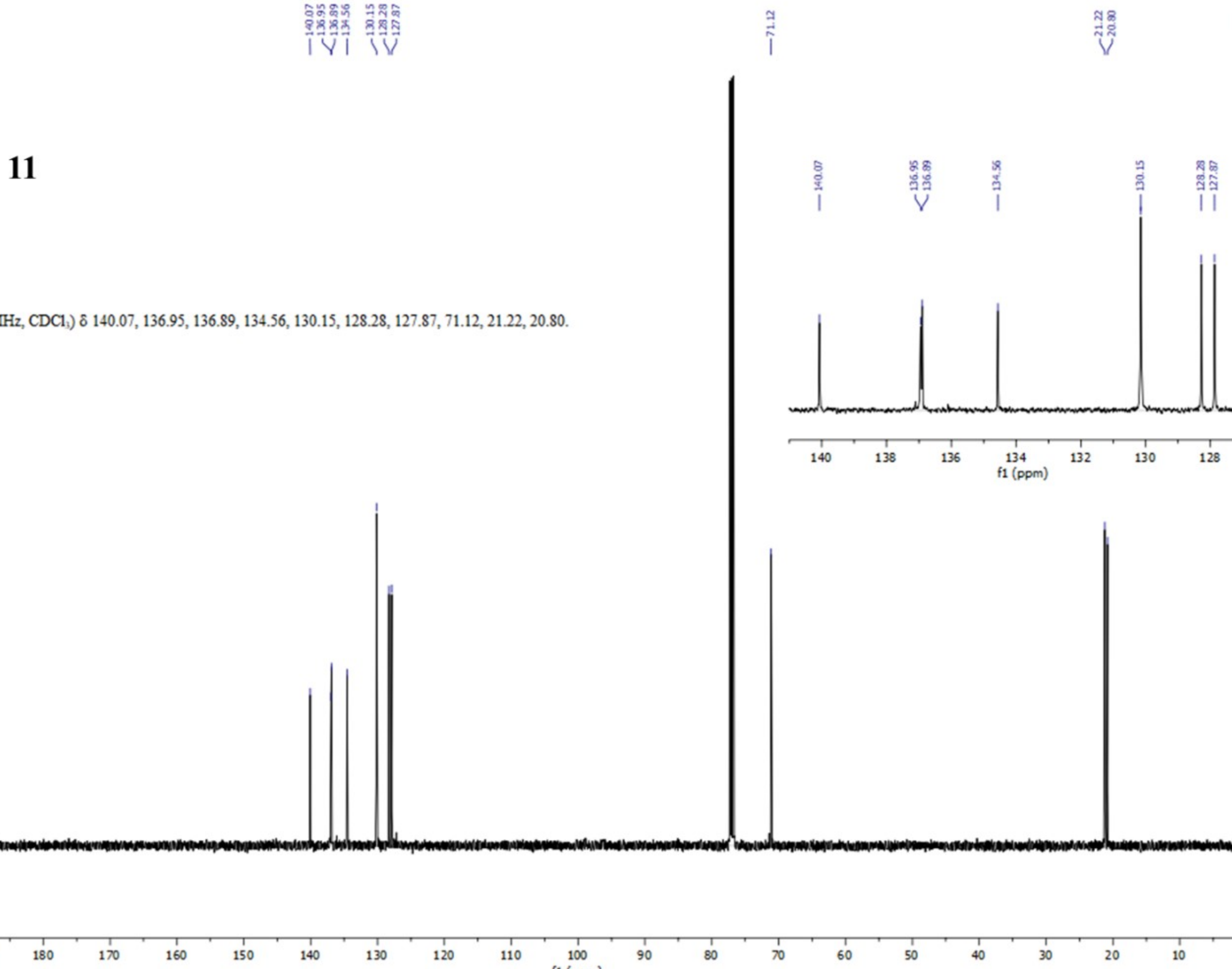

^{13}C NMR (126 MHz, $CDCl_3$) δ 140.07, 136.95, 136.89, 134.56, 130.15, 128.28, 127.87, 71.12, 21.22, 20.80.

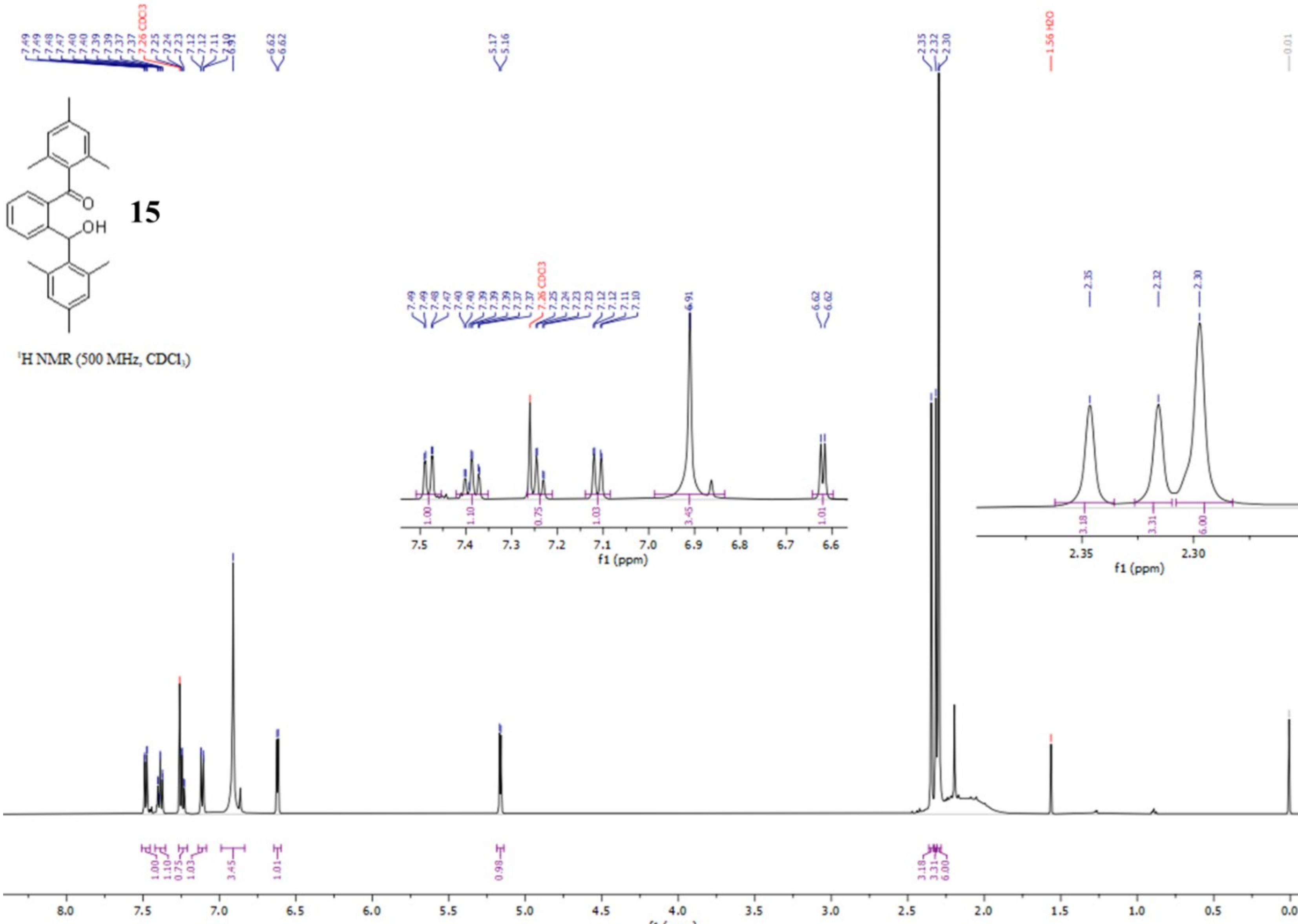

15

^{1}H NMR (500 MHz, $CDCl_3$)

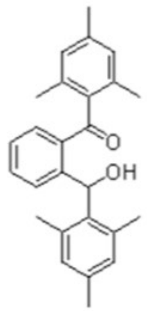

15

^{13}C NMR (126 MHz, $CDCl_3$) δ 205.17, 144.30, 139.12, 137.83, 137.23, 137.16, 136.72, 134.16, 133.61, 133.31, 129.97, 129.02, 128.99, 128.65, 127.67, 70.45, 21.50, 21.18, 20.86, 19.62.

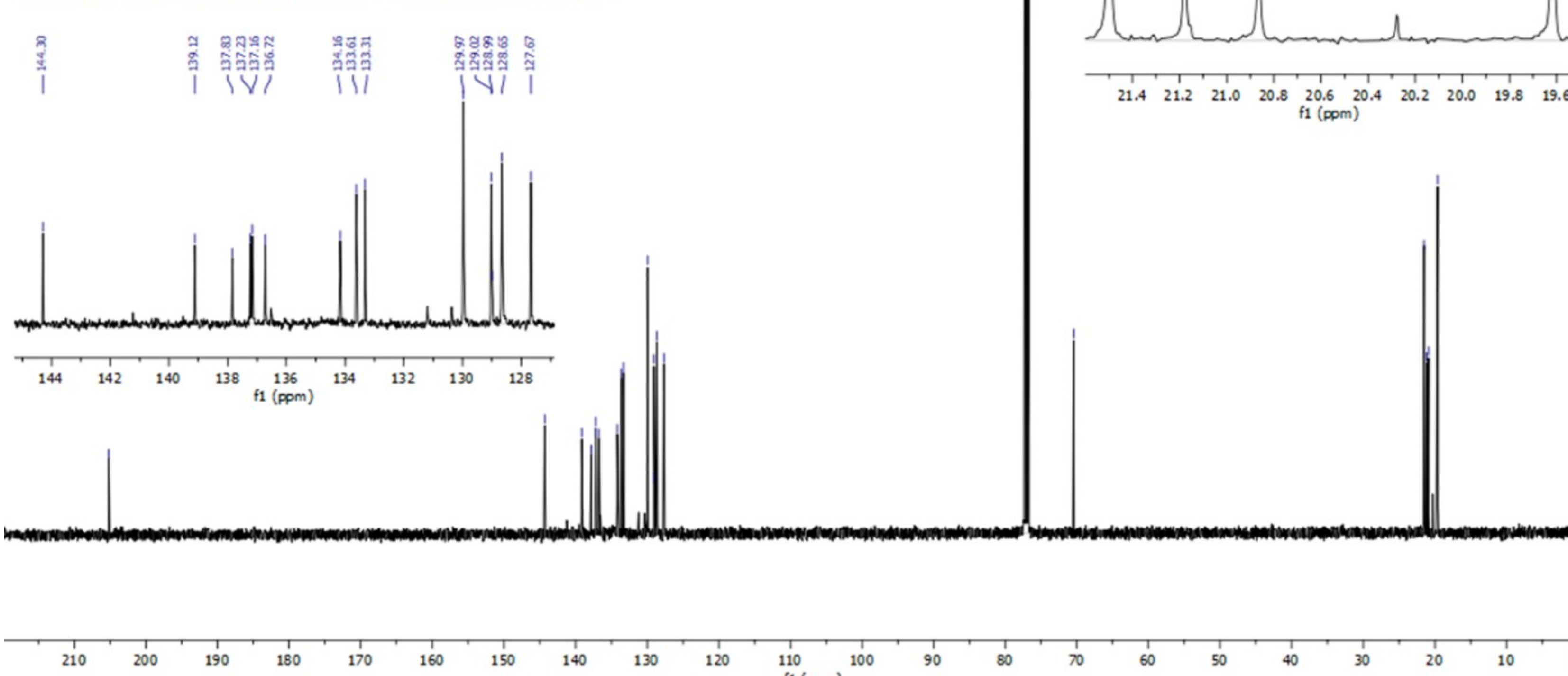

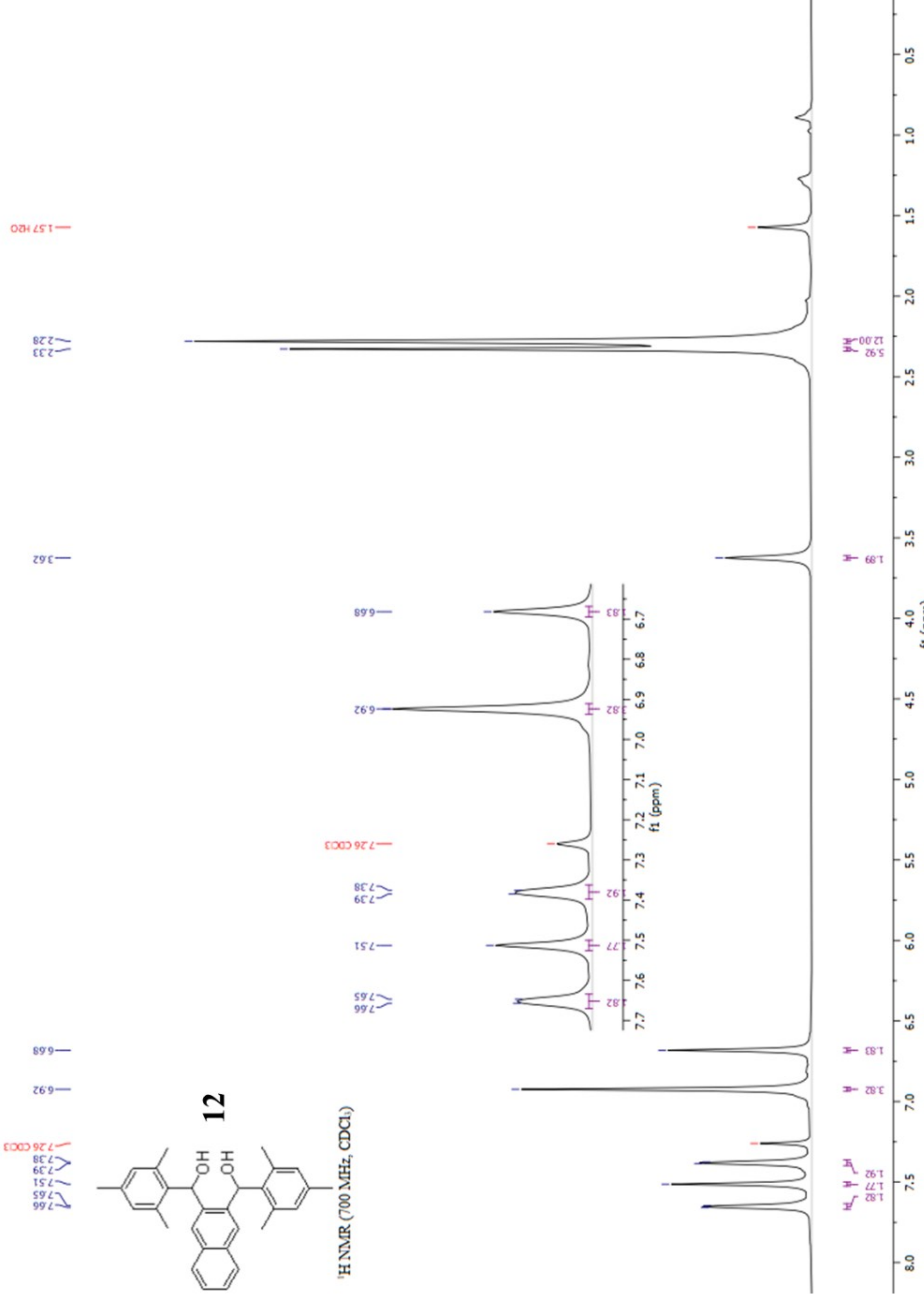
12
^{1}H NMR (700 MHz, CDCl$_3$)
1.57 H2O
2.28
2.33
3.62
6.68
6.92
7.26 CDCl3
7.38
7.39
7.51
7.65
7.66
f1 (ppm)

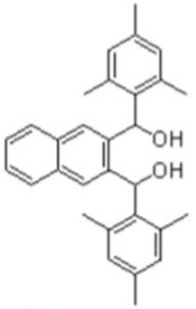

12

^{13}C NMR (176 MHz, $CDCl_3$) δ 137.80, 137.04, 136.97, 134.66, 132.60, 130.25, 127.87, 126.13, 71.27, 21.22, 20.85.

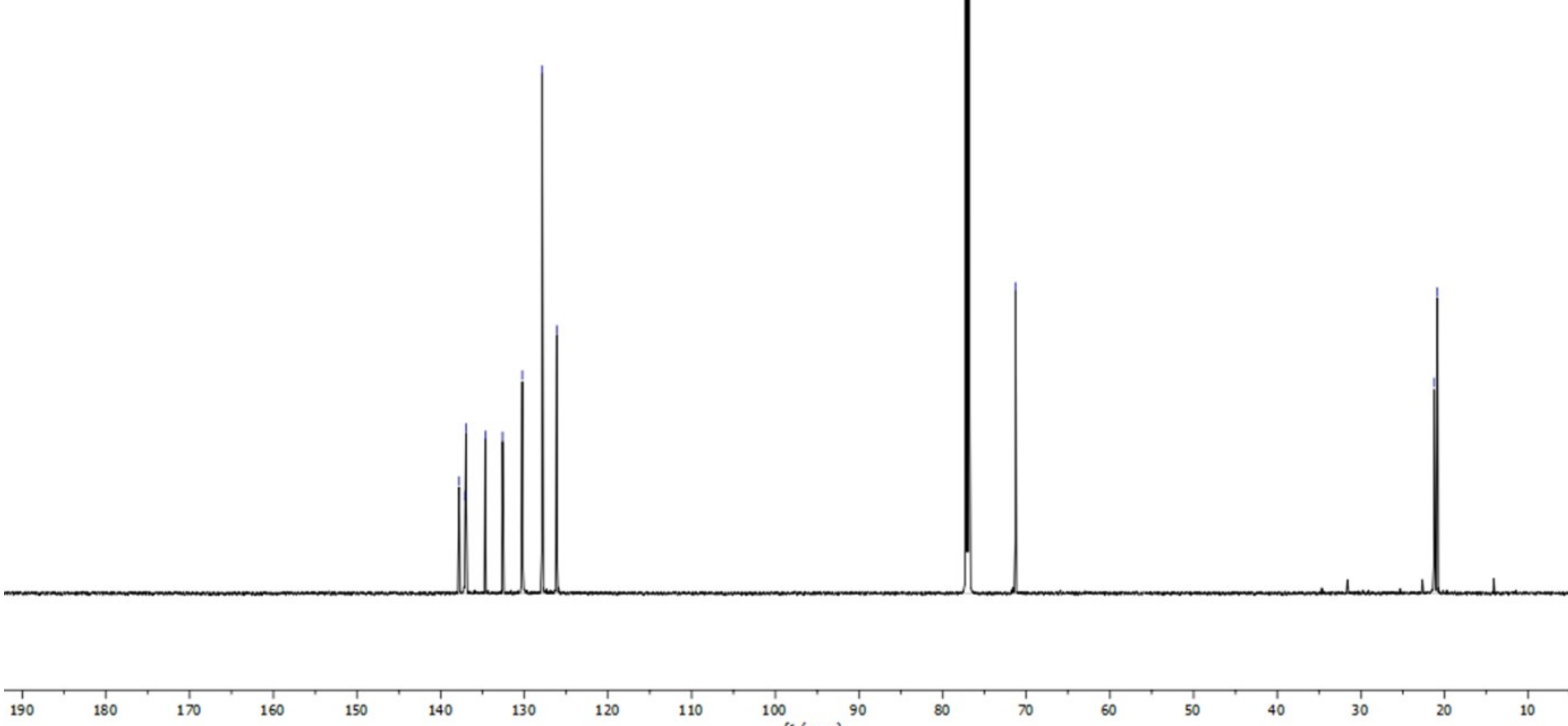

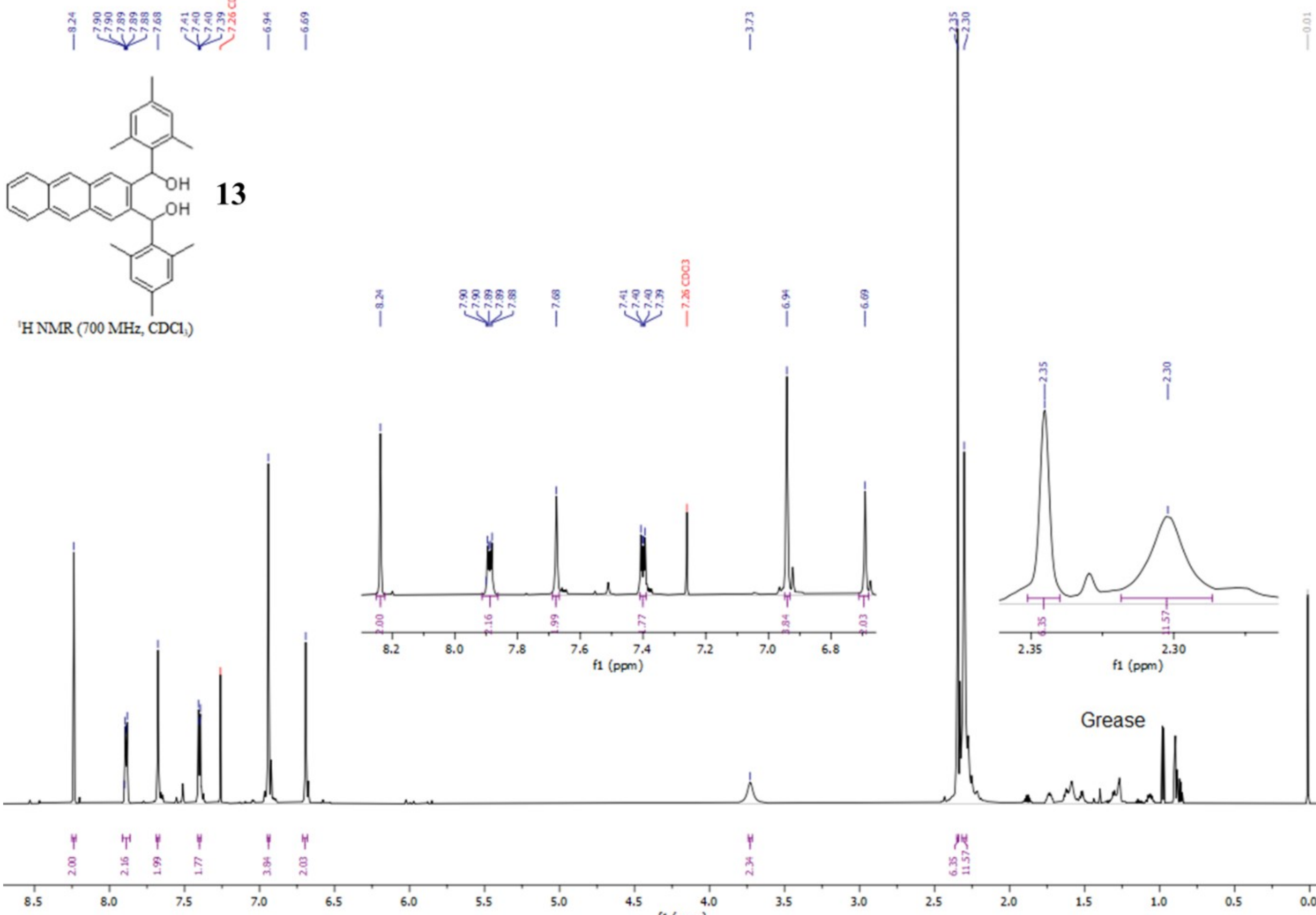

13

^{1}H NMR (700 MHz, $CDCl_3$)

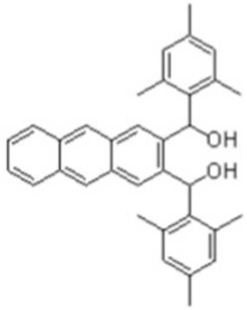

13

^{13}C NMR (176 MHz, $CDCl_3$) δ 137.36, 137.08, 136.99, 134.66, 131.92, 130.78, 130.29, 128.09, 128.05, 126.30, 125.39, 71.33, 21.22, 20.87.

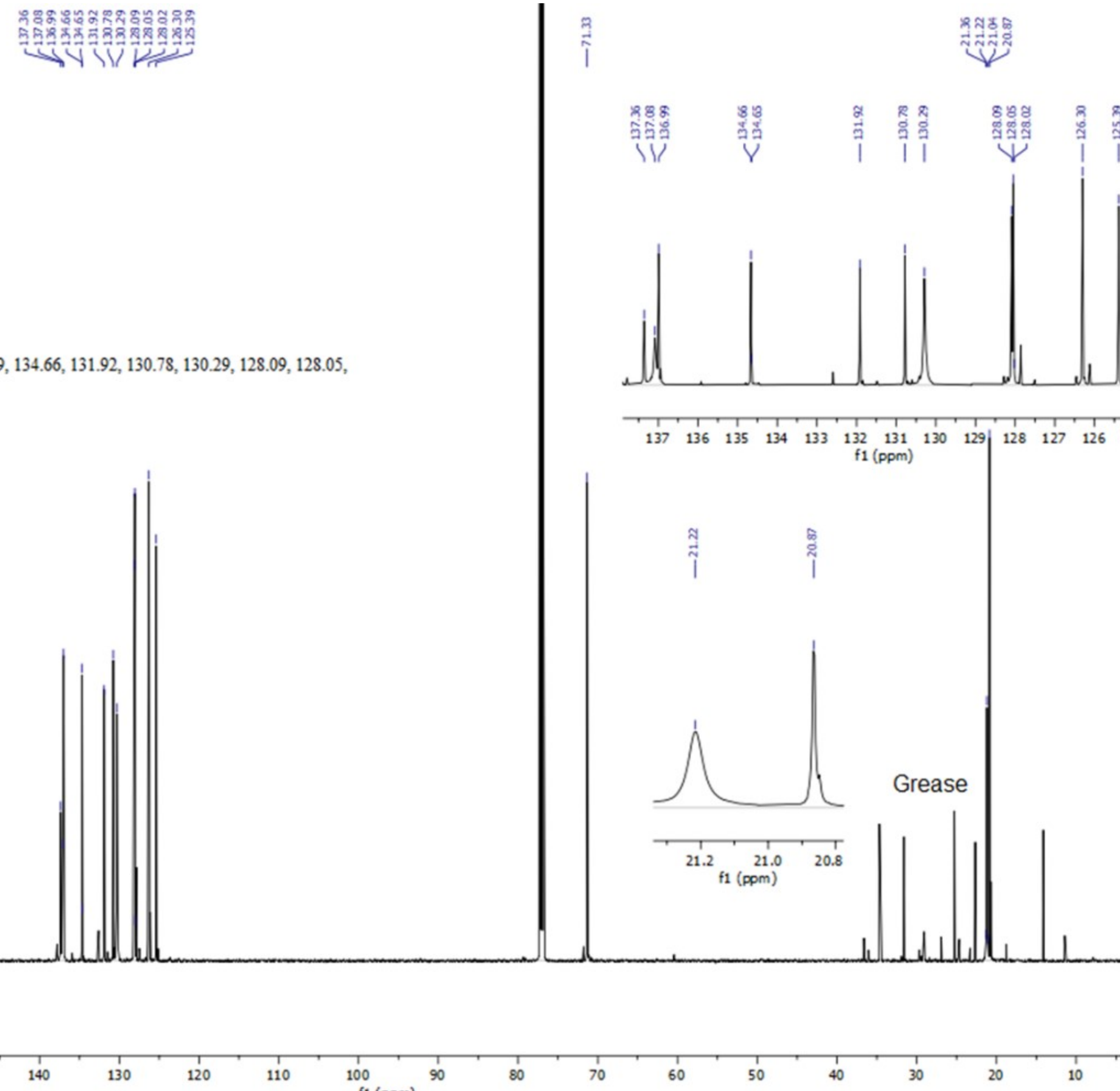

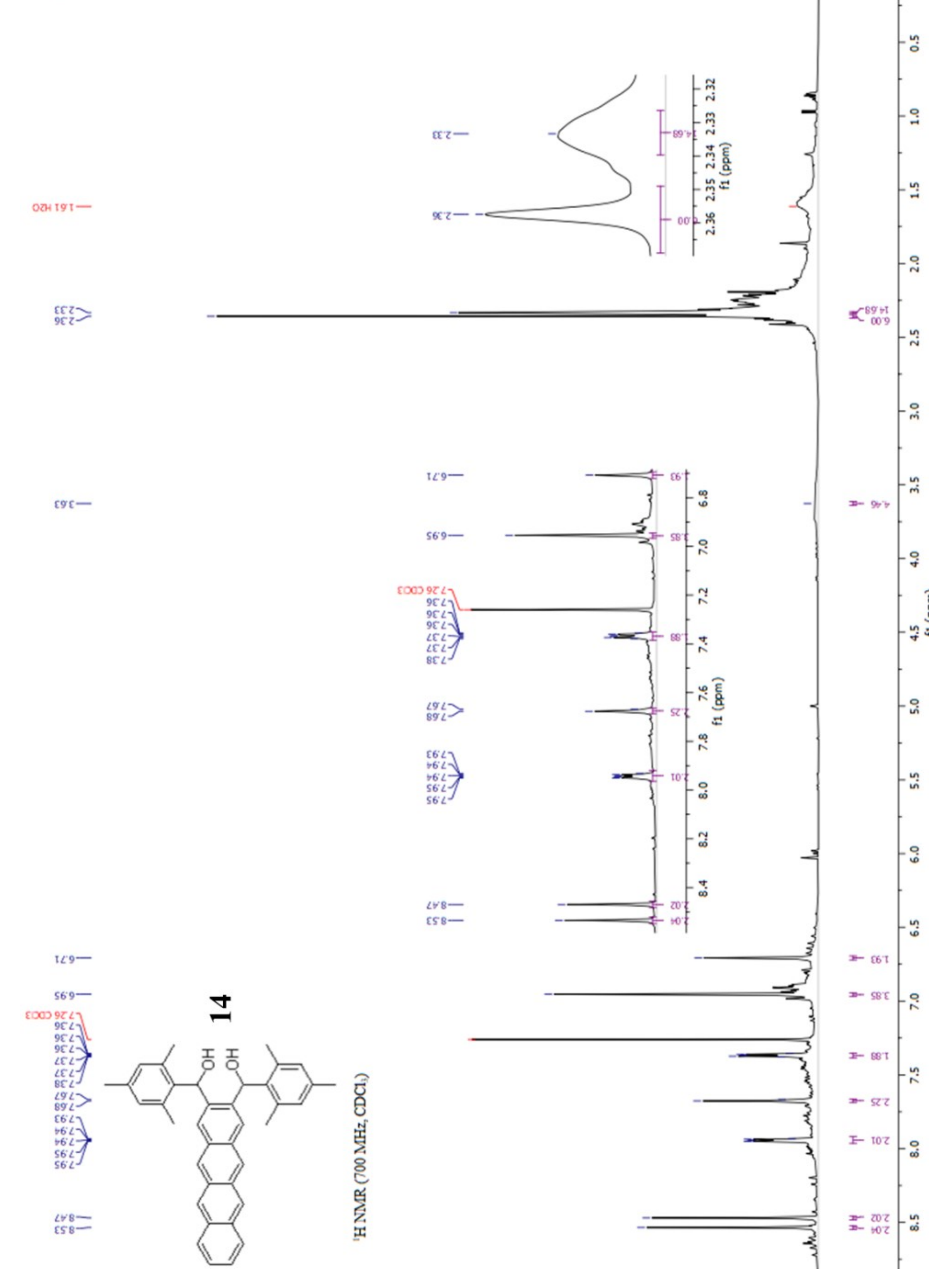
14
¹H NMR (700 MHz, CDCl₃)
f1 (ppm)

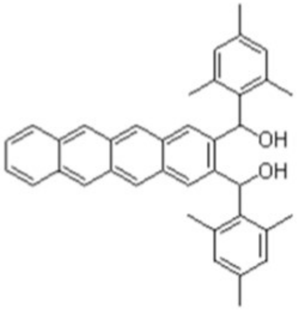

14

^{13}C NMR (176 MHz, $CDCl_3$) δ 137.17, 137.10, 137.07, 134.65, 131.50, 130.62, 130.44, 130.34, 128.28, 128.25, 126.48, 126.26, 125.16, 71.39, 21.25, 20.90.

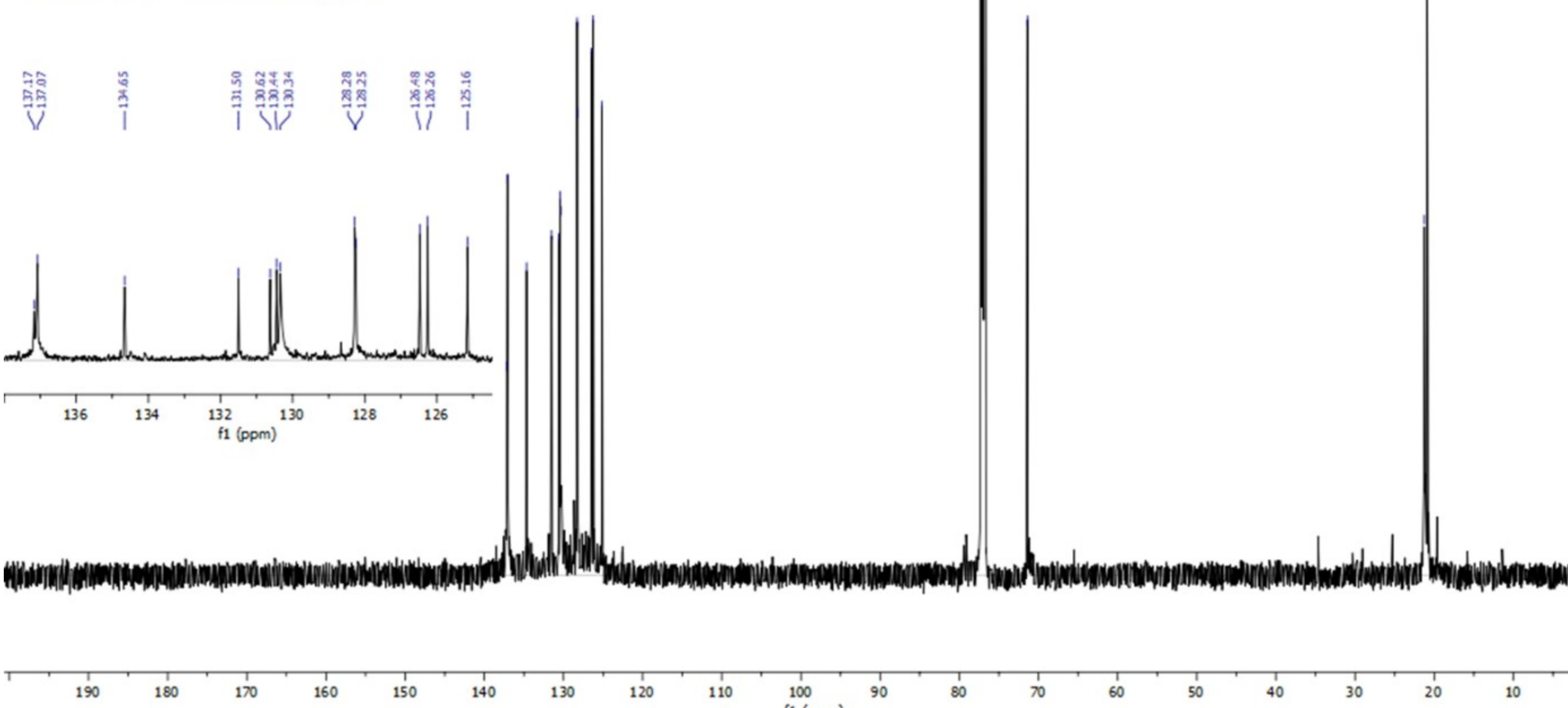

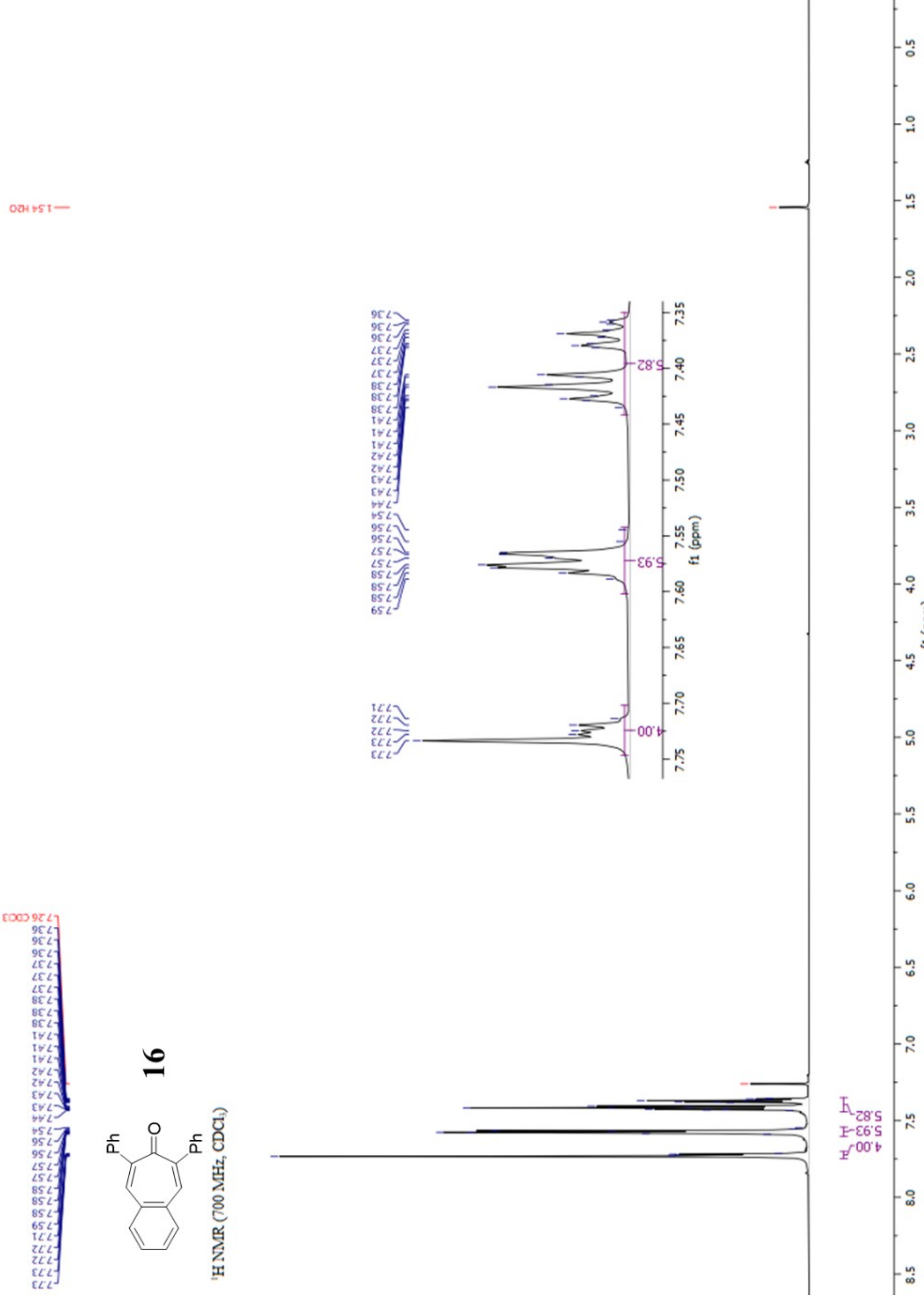
16
1H NMR (700 MHz, CDCl3)
f1 (ppm)
1.54 H2O
7.26 CDCl3

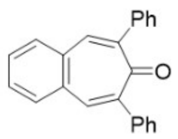

16

^{13}C NMR (176 MHz, $CDCl_3$) δ 187.27, 145.75, 140.77, 139.50, 134.68, 133.49, 129.73, 129.29, 128.06, 127.80.

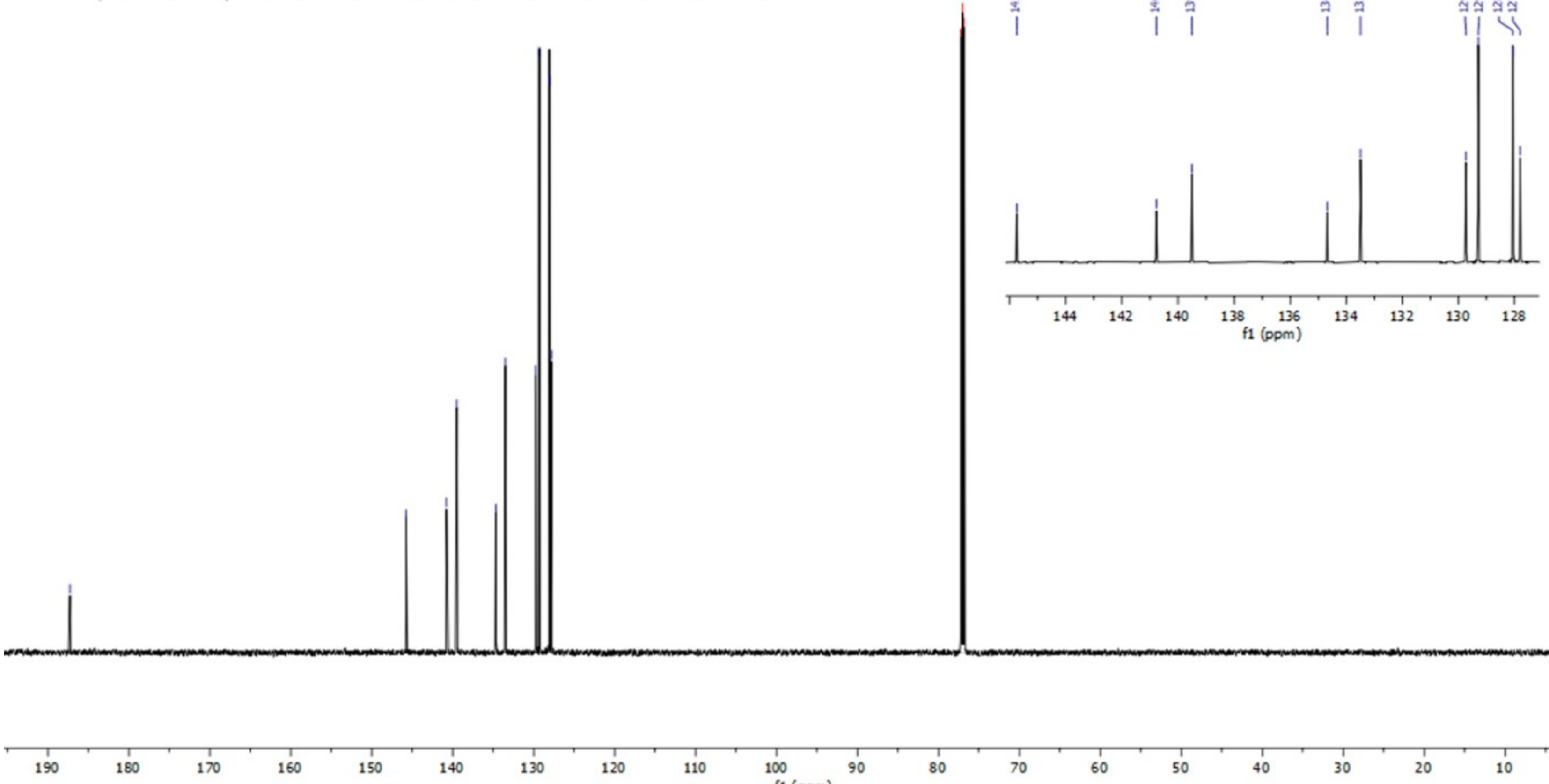

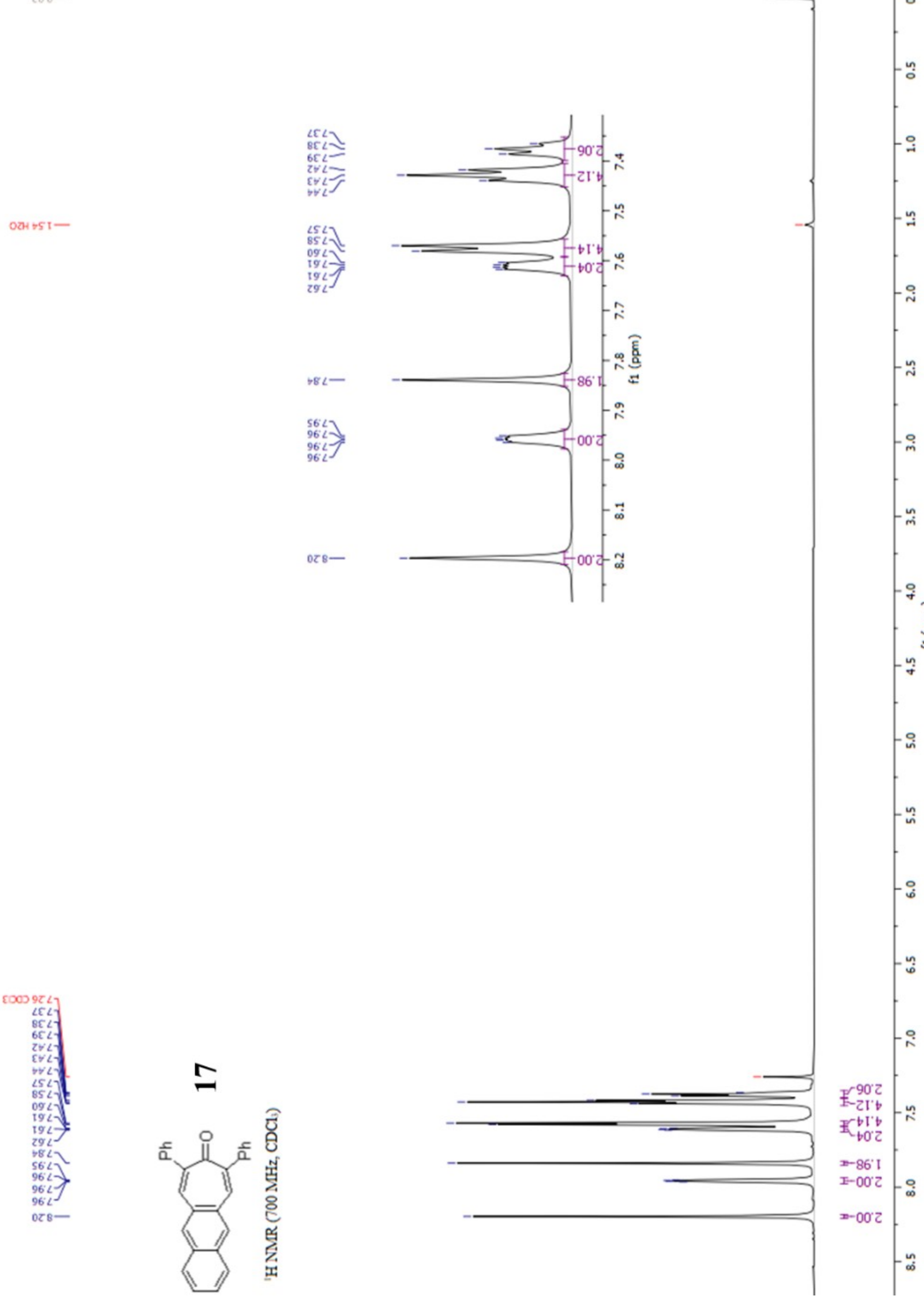

17

^1H NMR (700 MHz, $CDCl_3$)

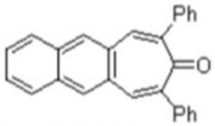

17

^{13}C NMR (176 MHz, $CDCl_3$) δ 188.04, 144.24, 141.04, 139.95, 133.69, 133.21, 132.07, 129.33, 128.04, 128.02, 127.87, 127.68.

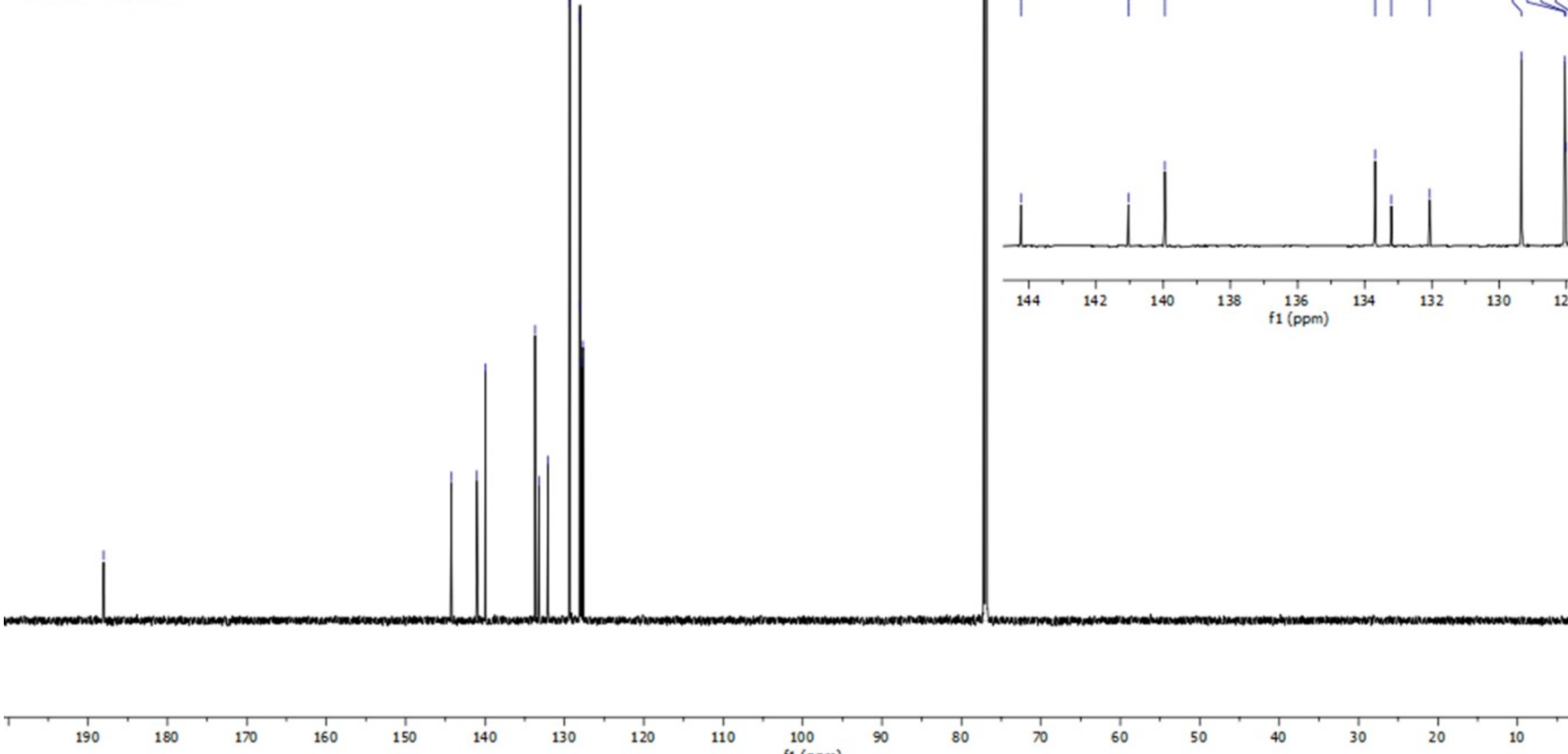

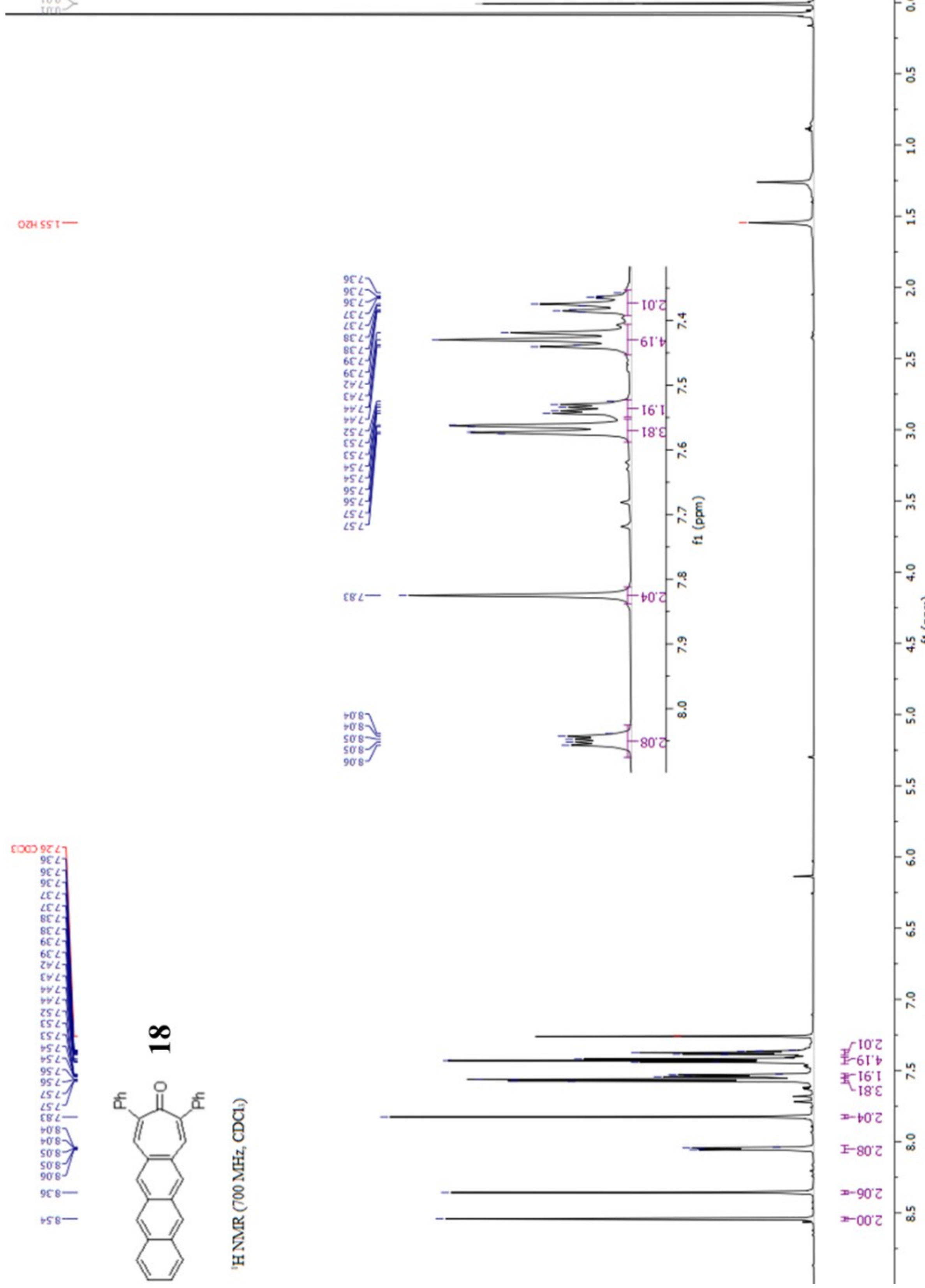

18

^{1}H NMR (700 MHz, $CDCl_3$)

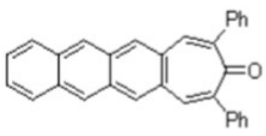

18

^{13}C NMR (176 MHz, $CDCl_3$) δ 188.39, 143.36, 141.12, 140.12, 134.39, 132.75, 131.53, 130.67, 129.34, 128.33, 128.02, 127.62, 126.86, 126.46.

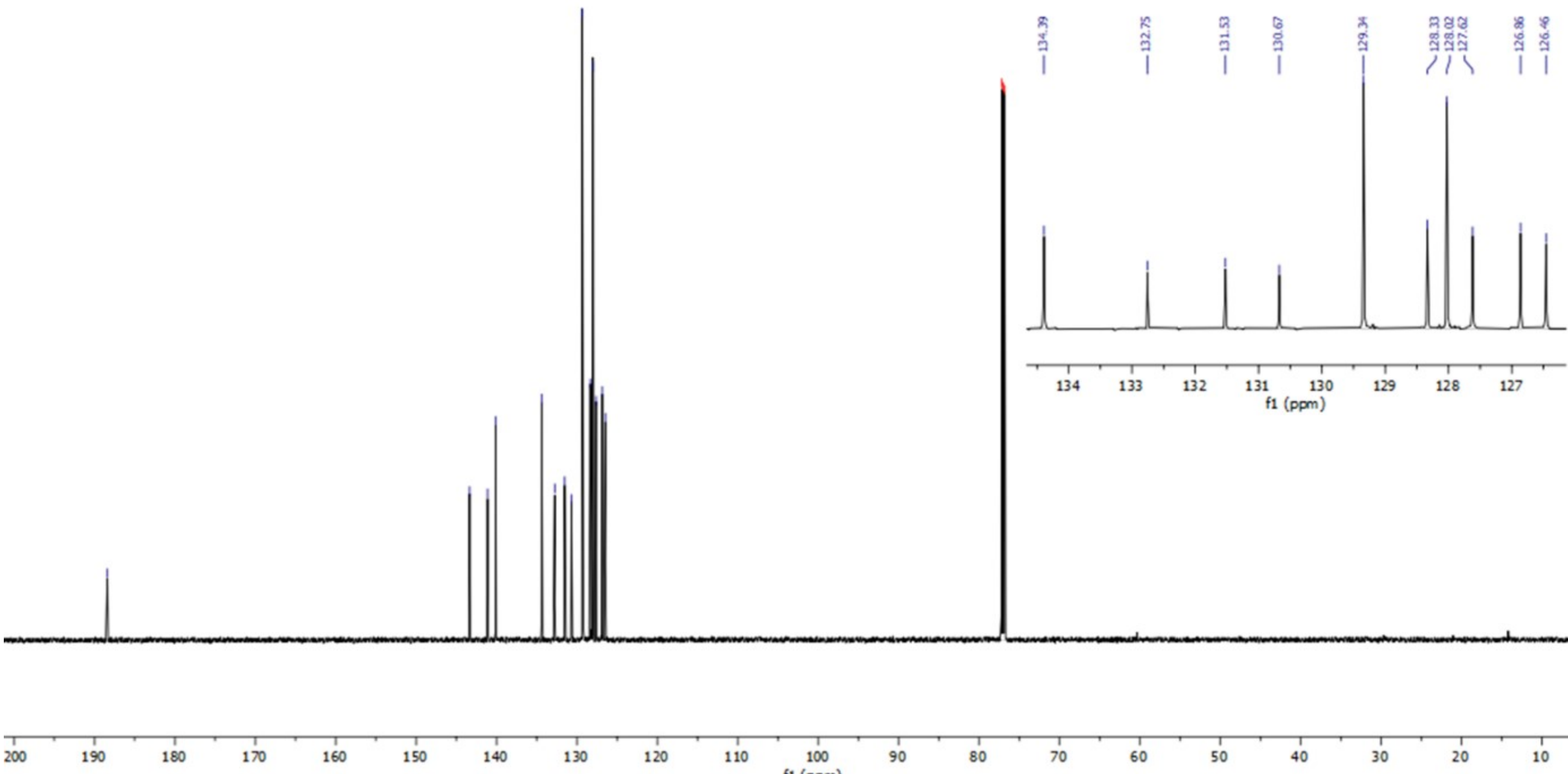

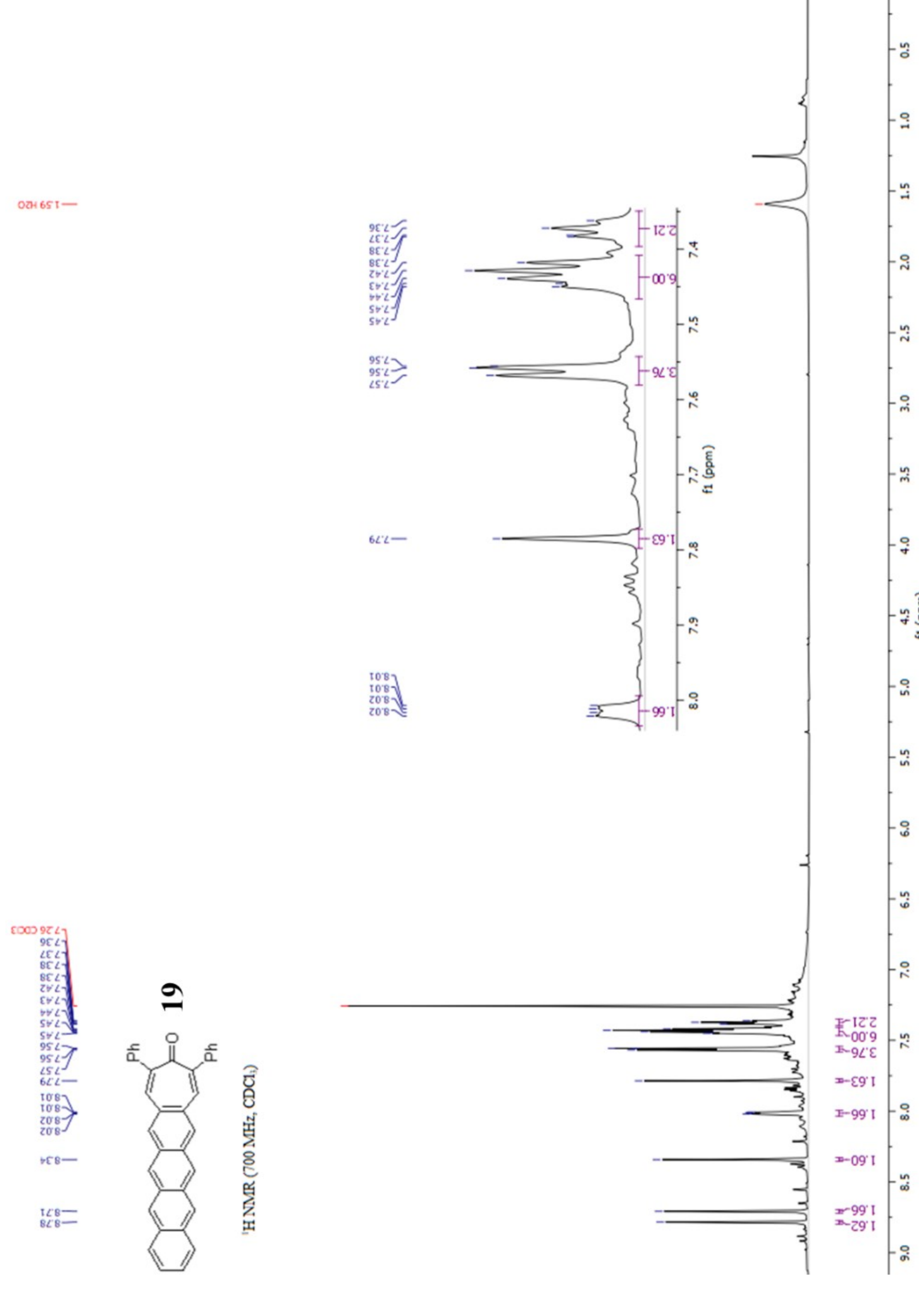
19
Ph
Ph
O
^{1}H NMR (700 MHz, CDCl$_3$)
1.59 H2O
7.26 CDCl3
8.78
8.71
8.34
8.02
8.02
8.01
8.01
7.79
7.57
7.56
7.56
7.45
7.45
7.44
7.43
7.42
7.38
7.38
7.37
7.36
f1 (ppm)

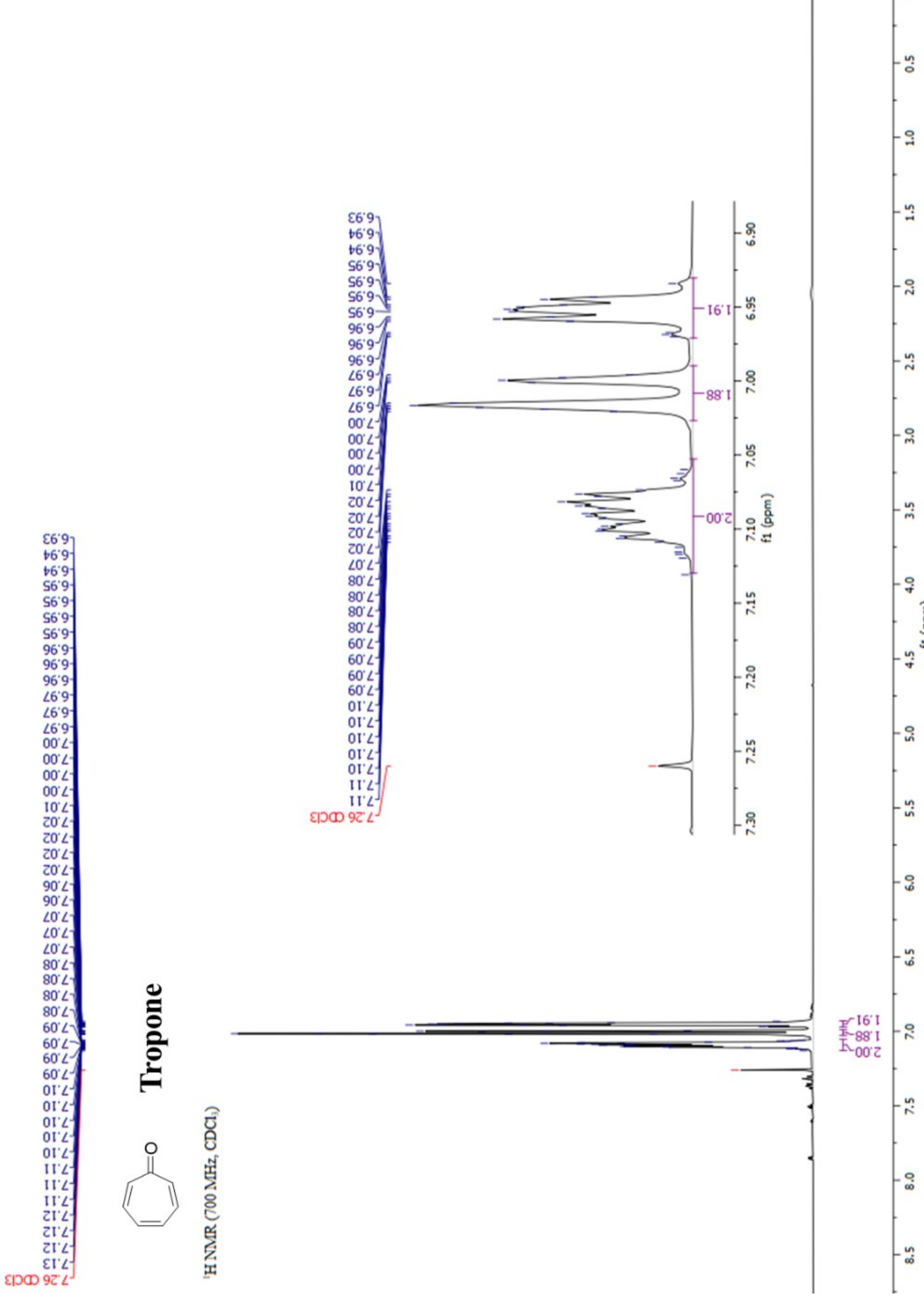
Tropone
¹H NMR (700 MHz, CDCl₃)

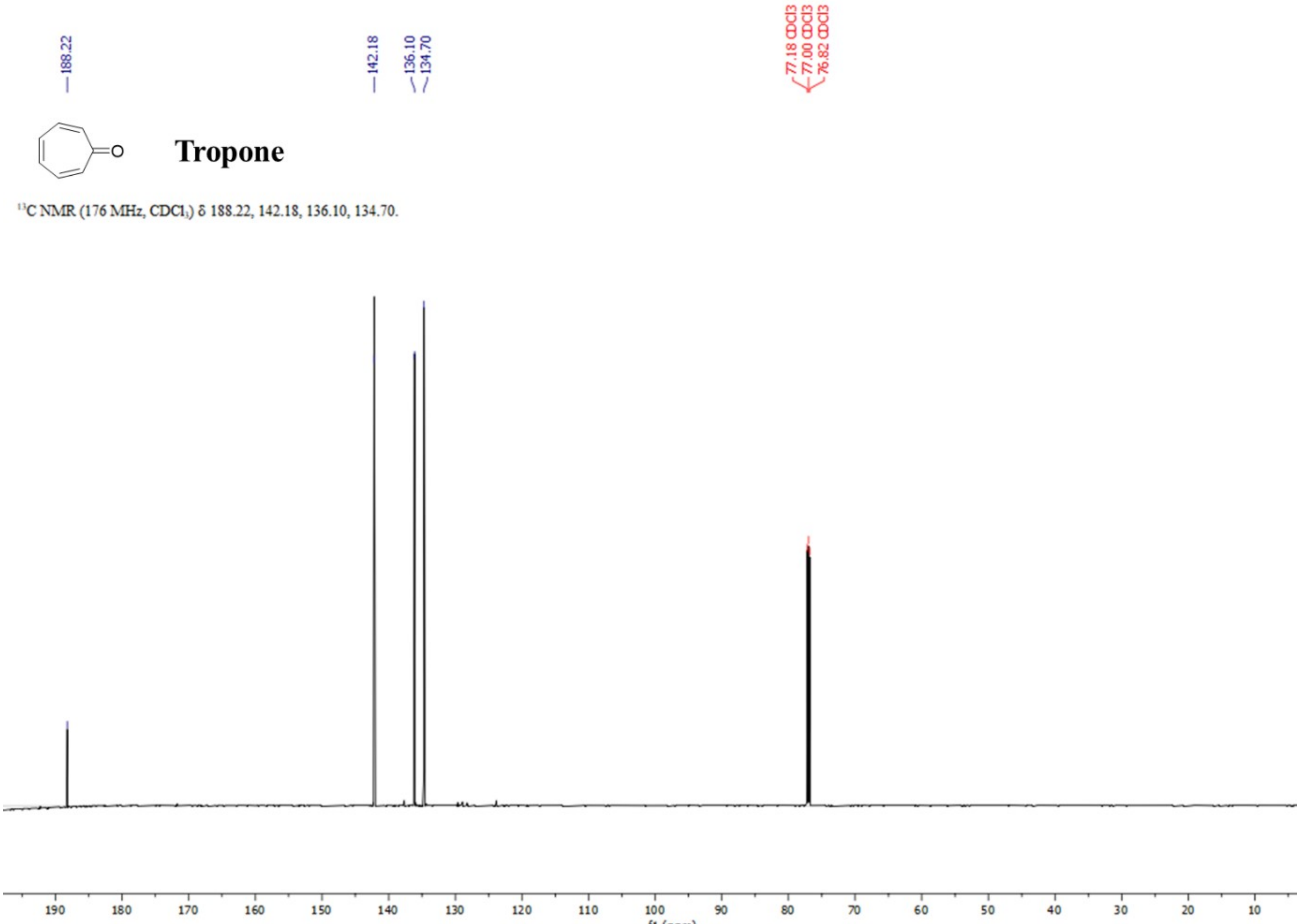

Tropone

^{13}C NMR (176 MHz, $CDCl_3$) δ 188.22, 142.18, 136.10, 134.70.

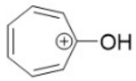

Hydroxytropylium ion

^1H NMR (700 MHz, $CDCl_3$)

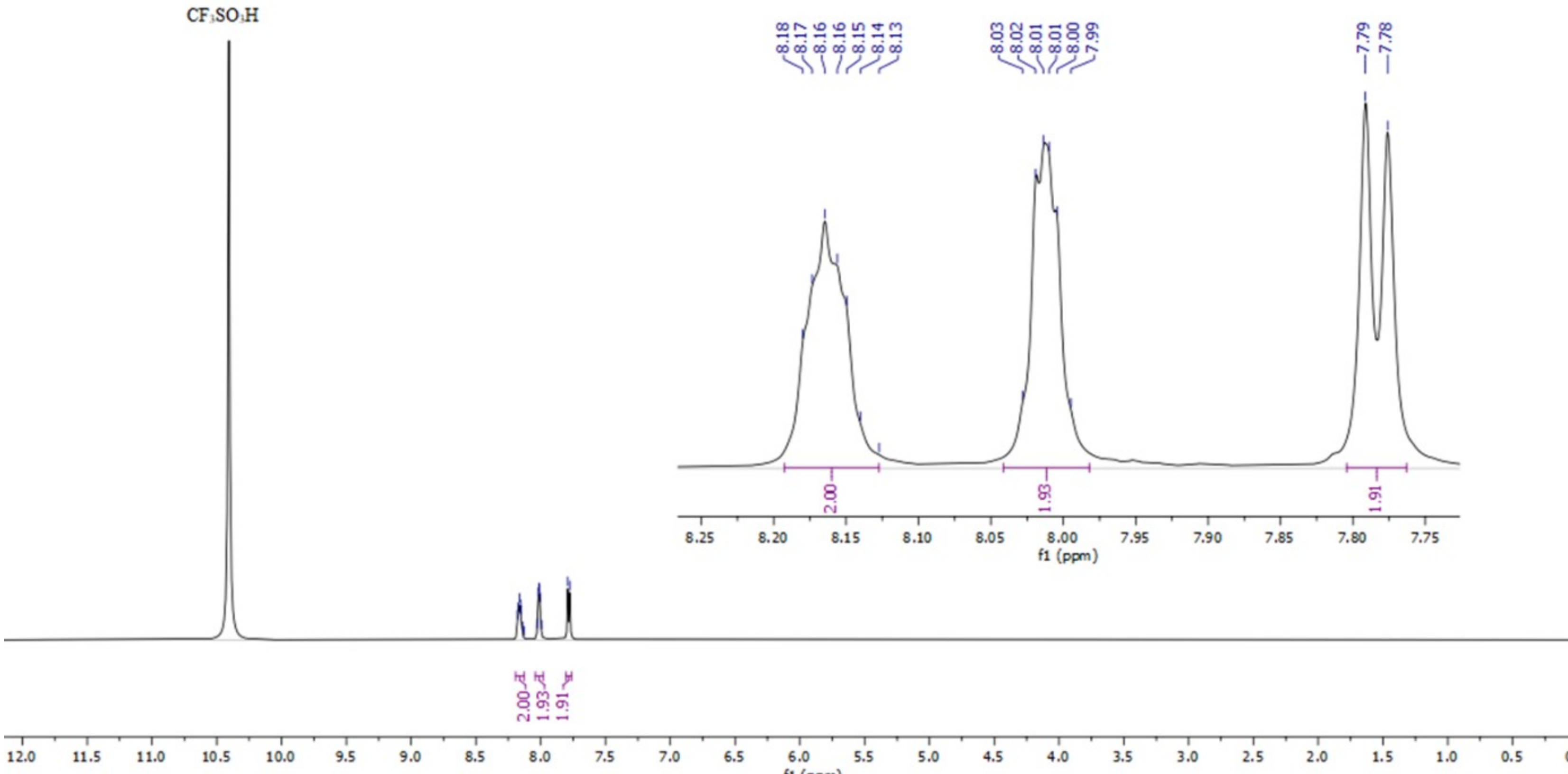

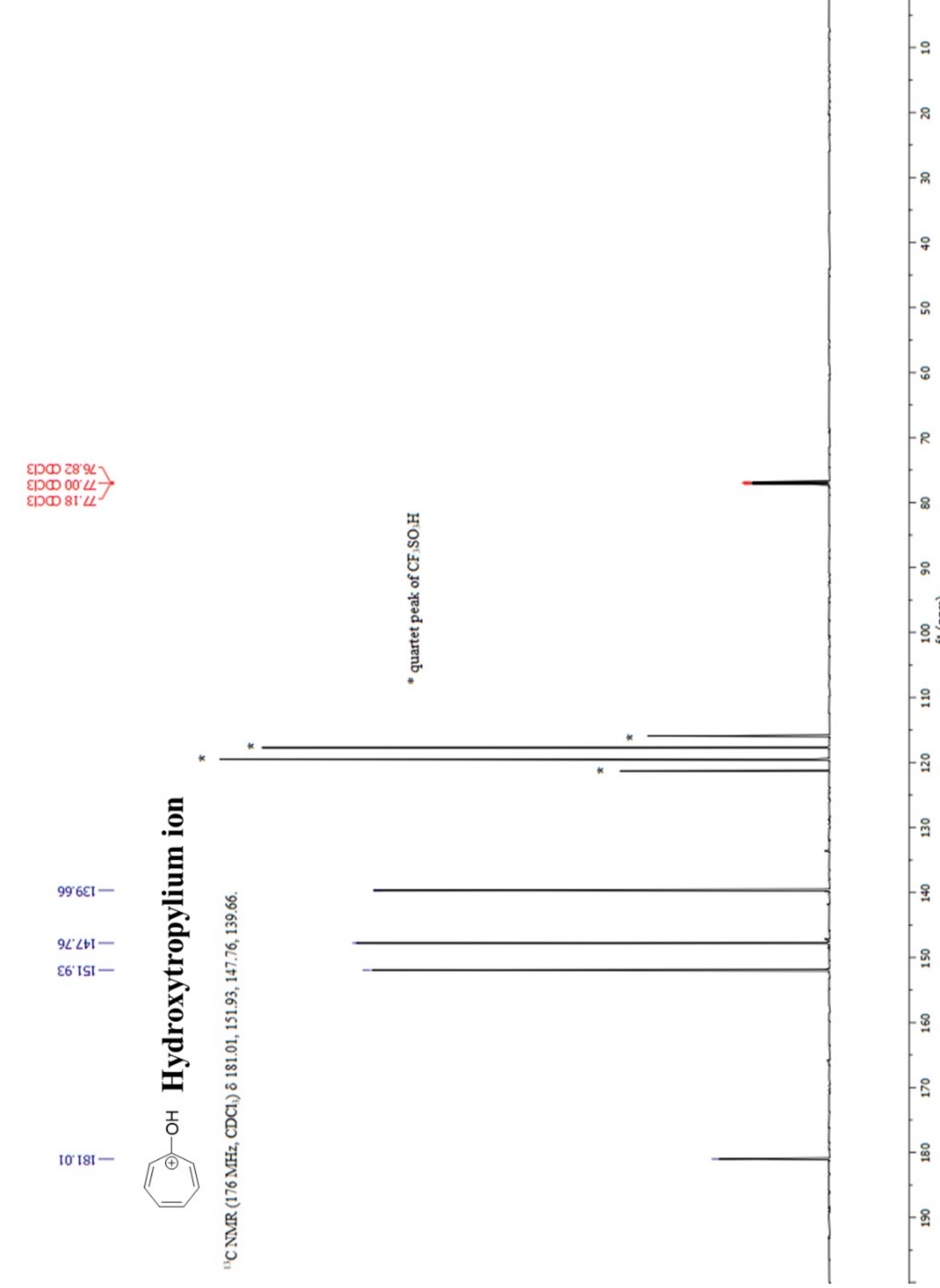

Hydroxytropylium ion
^{13}C NMR (176 MHz, $CDCl_3$) δ 181.01, 151.93, 147.76, 139.66.
* quartet peak of CF_3SO_3H
181.01
151.93
147.76
139.66
77.18 CDCl3
77.00 CDCl3
76.82 CDCl3
f1 (ppm)

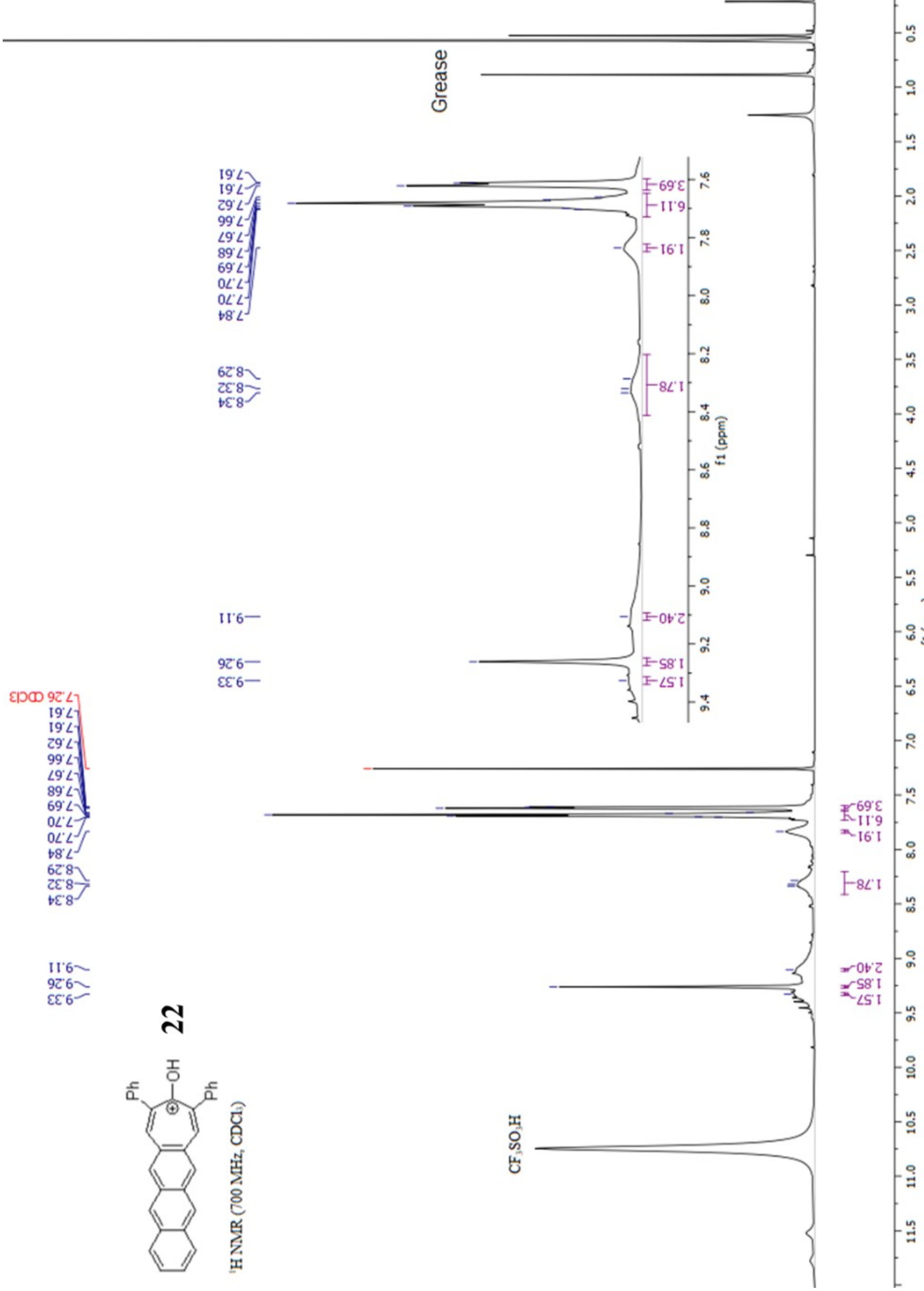

22
Ph
OH
Ph
1H NMR (700 MHz, CDCl3)
Grease
CF3SO3H
7.26 CDCl3
9.33
9.26
9.11
8.34
8.32
8.29
7.84
7.70
7.70
7.69
7.68
7.67
7.66
7.62
7.61
7.61
3.69
6.11
1.91
1.78
2.40
1.85
1.57
f1 (ppm)

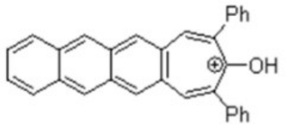

22

^{13}C NMR (176 MHz, $CDCl_3$) δ 179.75, 161.03, 141.94, 136.03, 135.35, 135.03, 131.97, 131.46, 130.90, 130.32, 129.62, 129.35, 129.15, 128.94.

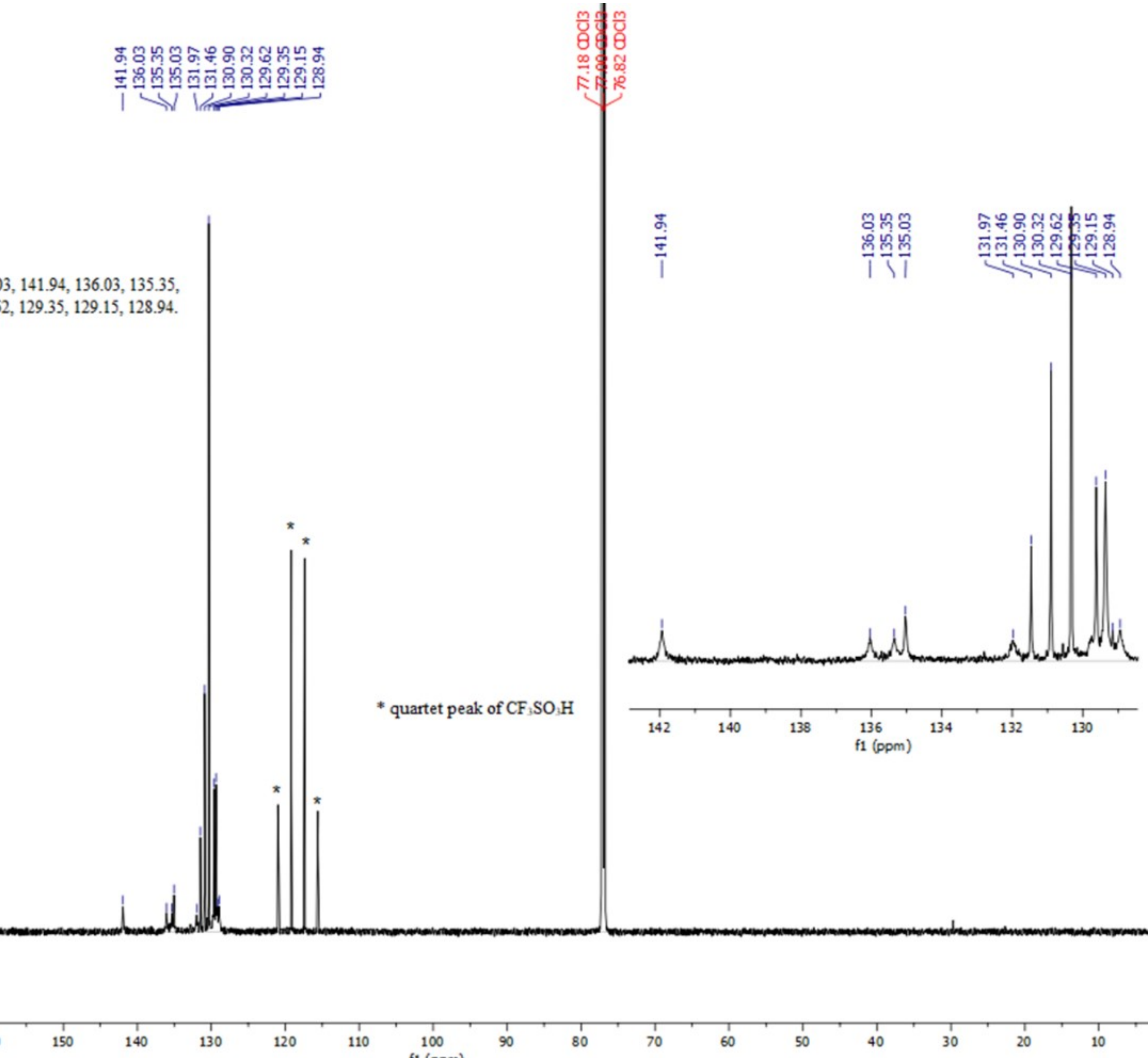

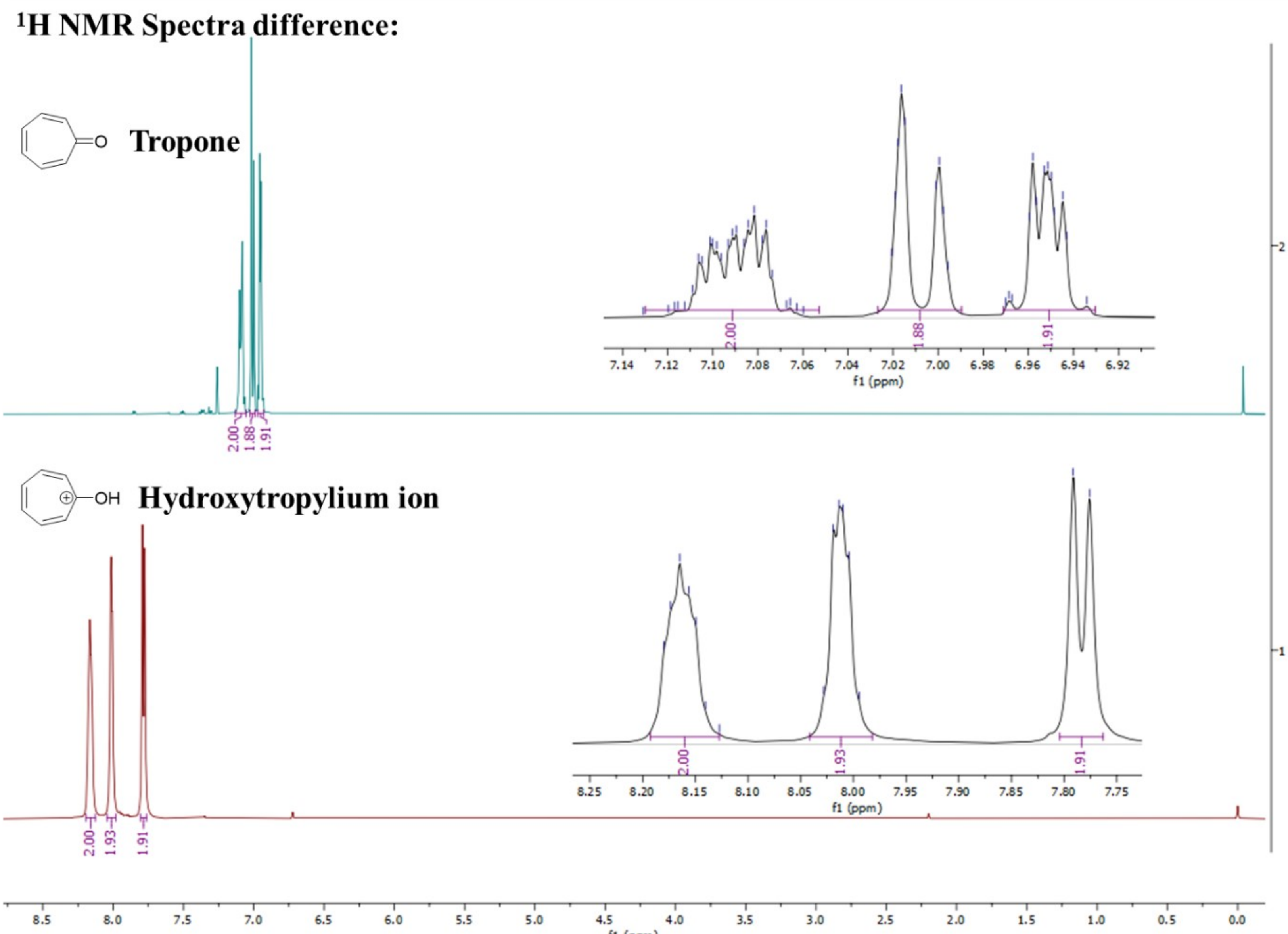

¹H NMR Spectra difference:
Tropone
Hydroxytropylium ion
OH
O
2.00
1.88
1.91
1.93
f1 (ppm)

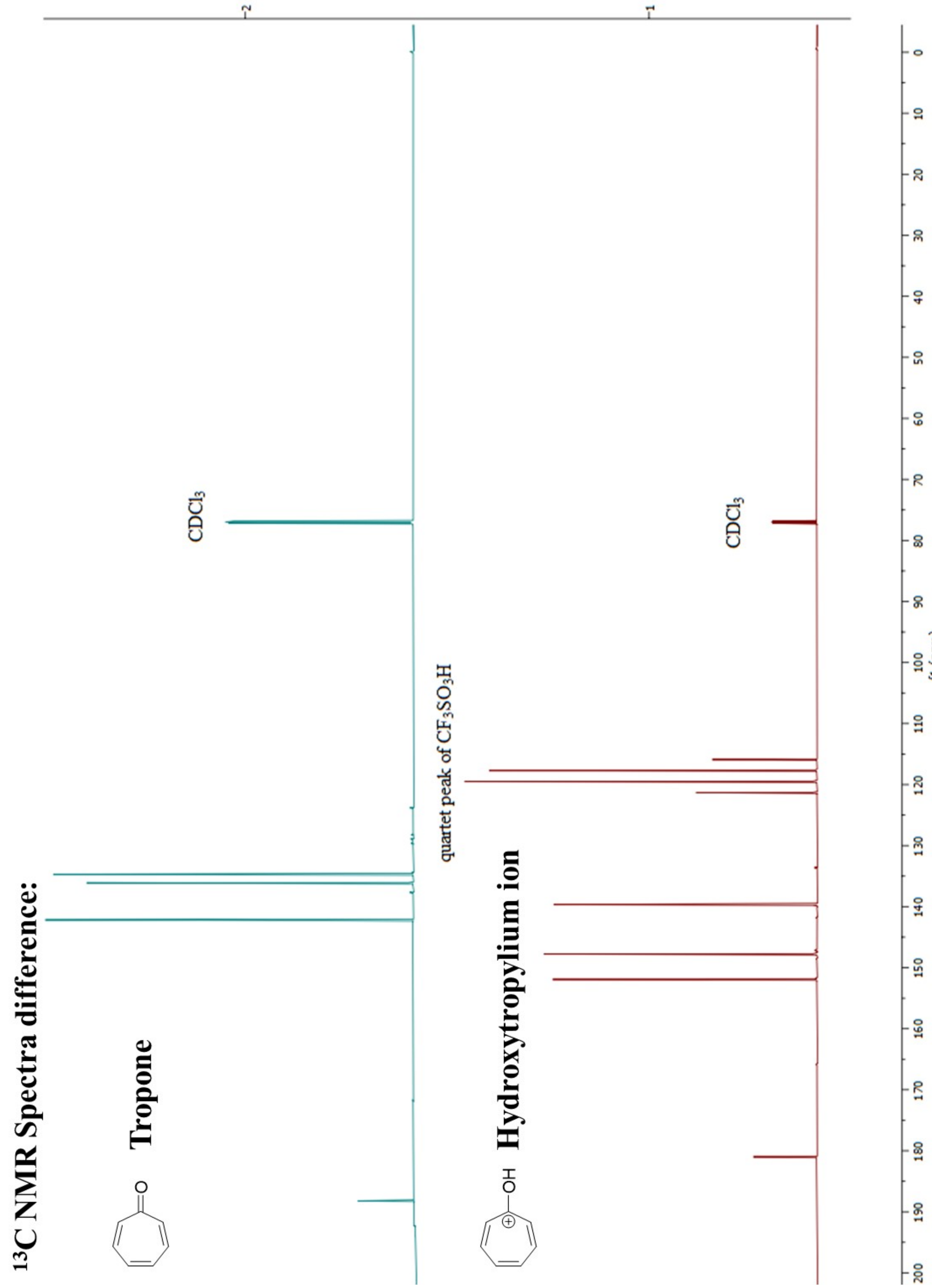

^{13}C NMR Spectra difference:
Tropone
Hydroxytropylium ion
CDCl$_3$
CDCl$_3$
quartet peak of CF$_3$SO$_3$H
OH

^{1}H NMR Spectra difference:

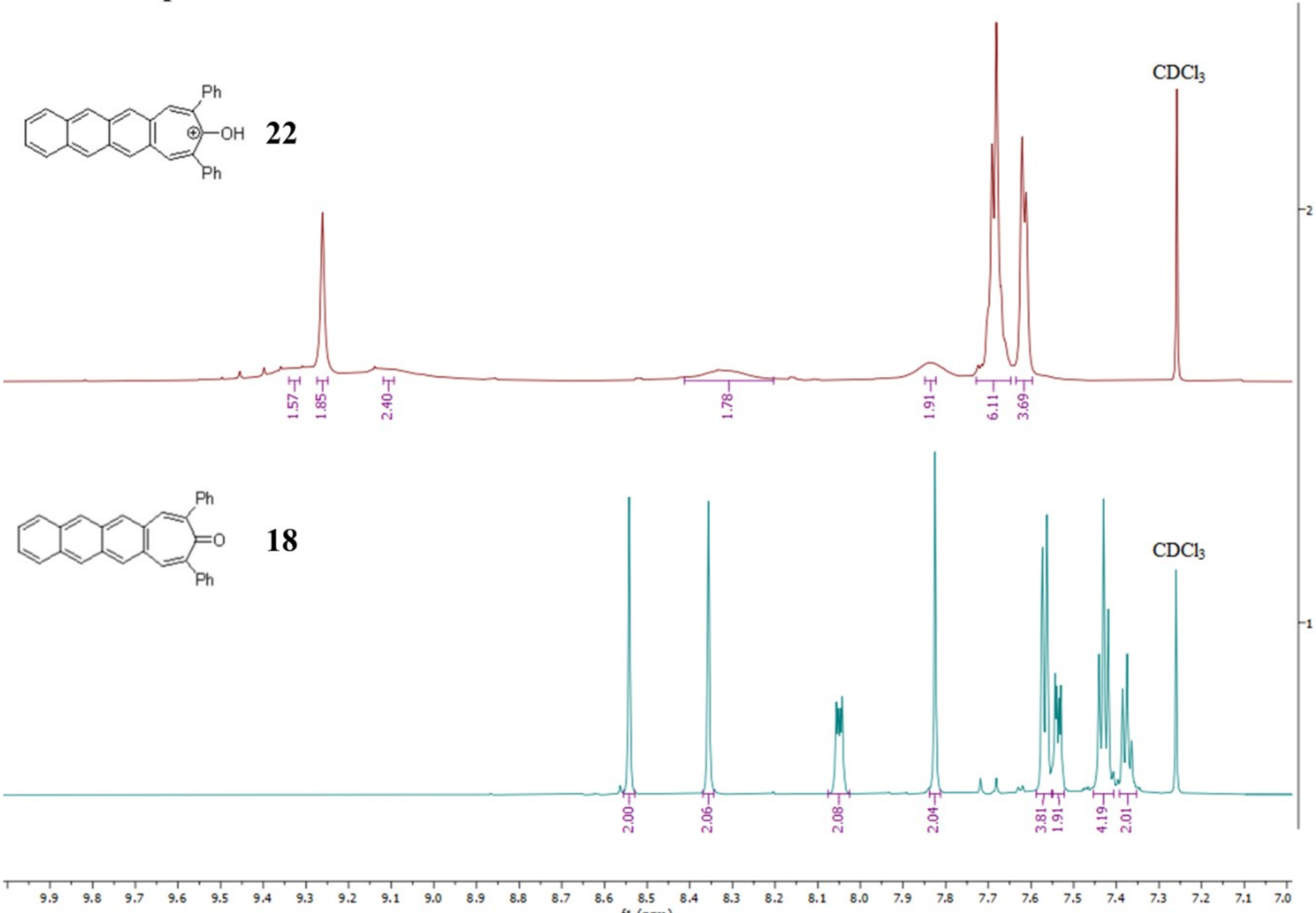

^{13}C NMR Spectra difference:

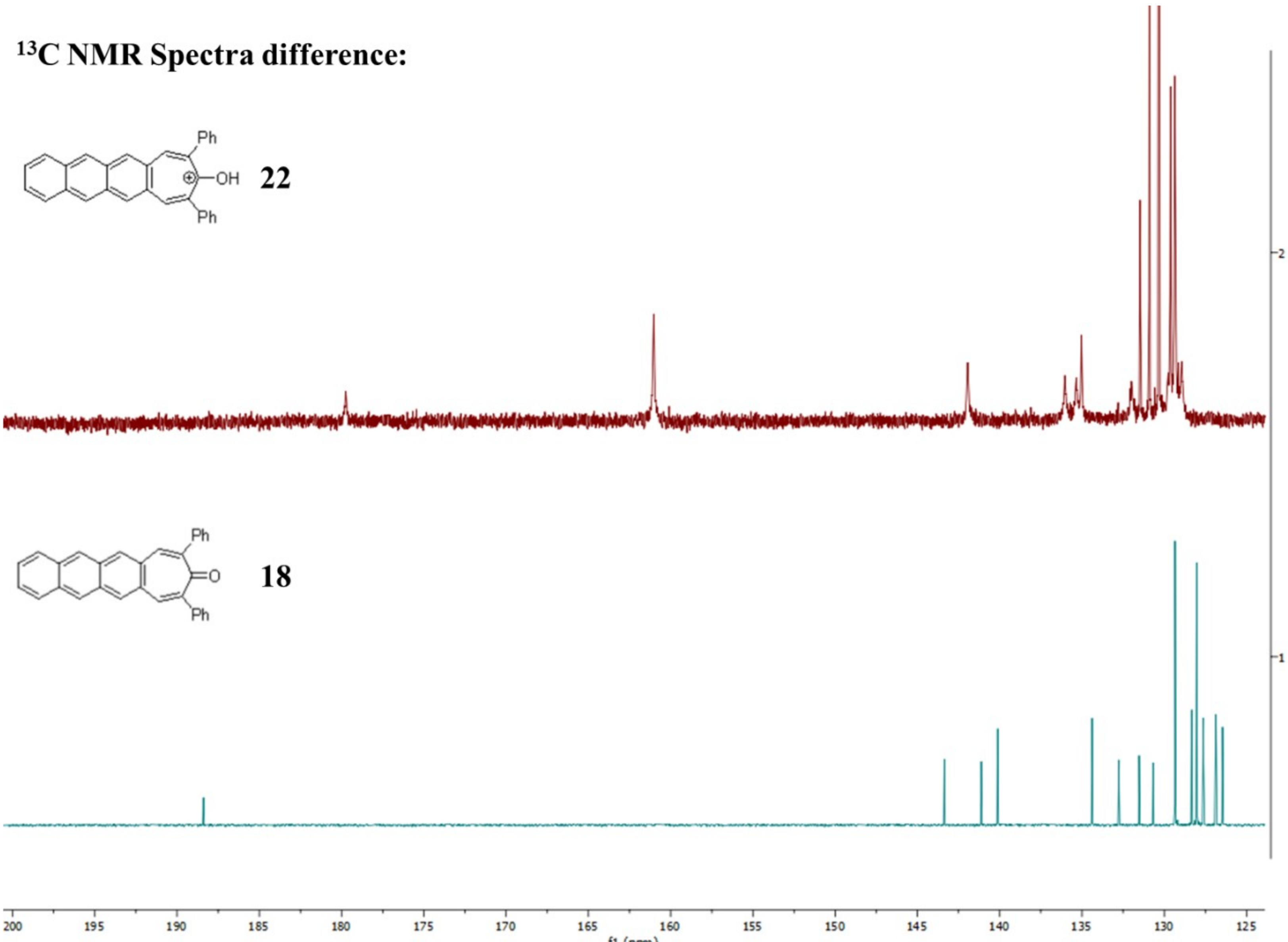

7. HRMS Spectra

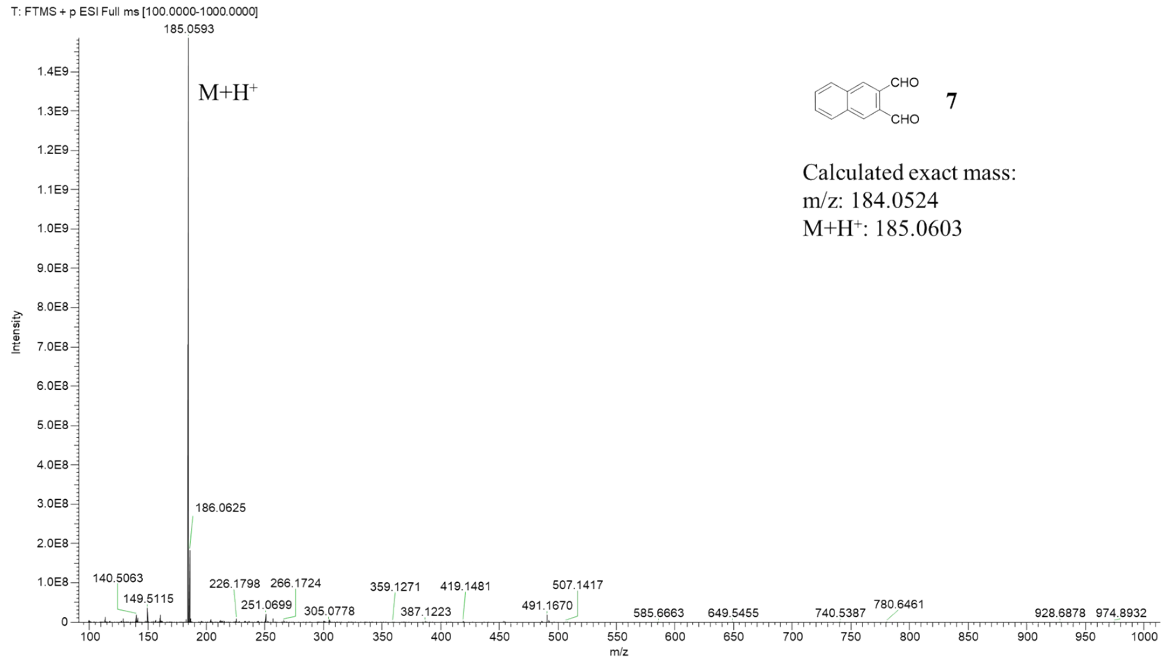

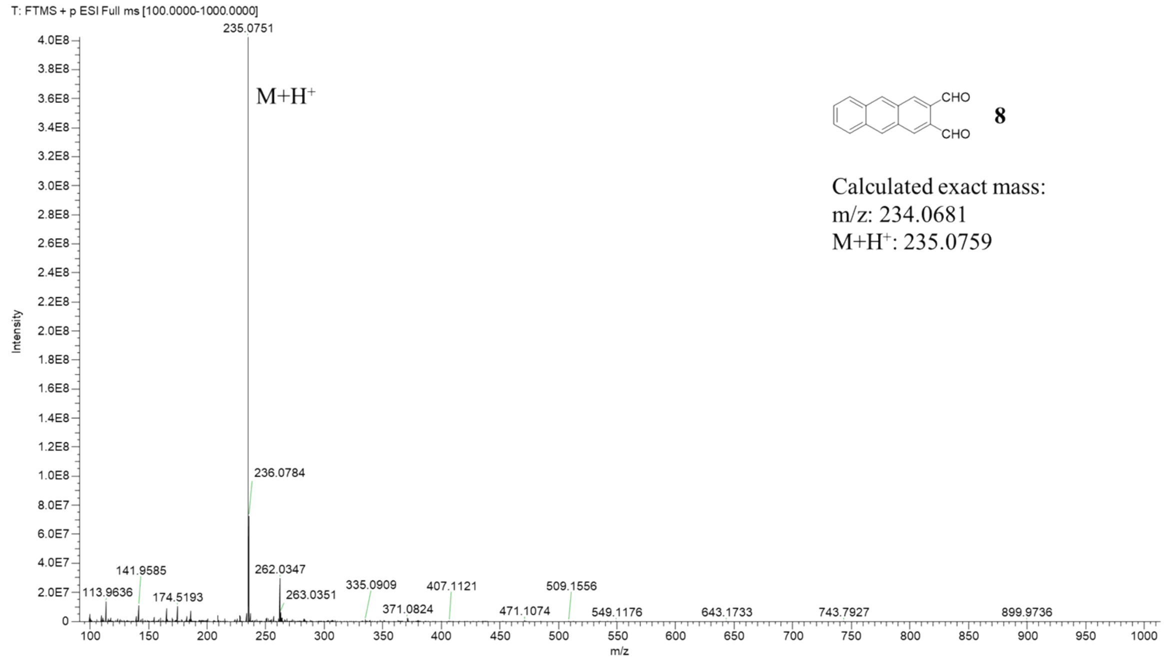

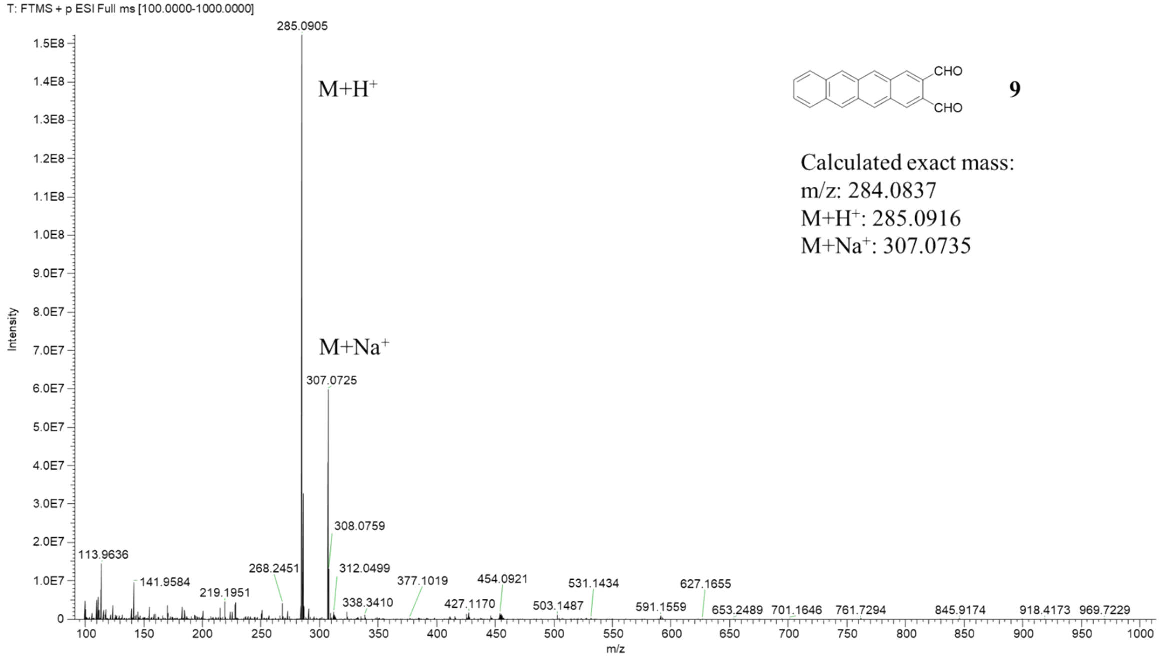
T: FTMS + p ESI Full ms [100.0000-1000.0000]
285.0905
M+H$^+$
M+Na$^+$
307.0725
308.0759
113.9636
141.9584
219.1951
268.2451
312.0499
338.3410
377.1019
427.1170
454.0921
503.1487
531.1434
591.1559
627.1655
653.2489
701.1646
761.7294
845.9174
918.4173
969.7229
Intensity
m/z
CHO
CHO
9
Calculated exact mass:
m/z: 284.0837
M+H$^+$: 285.0916
M+Na$^+$: 307.0735

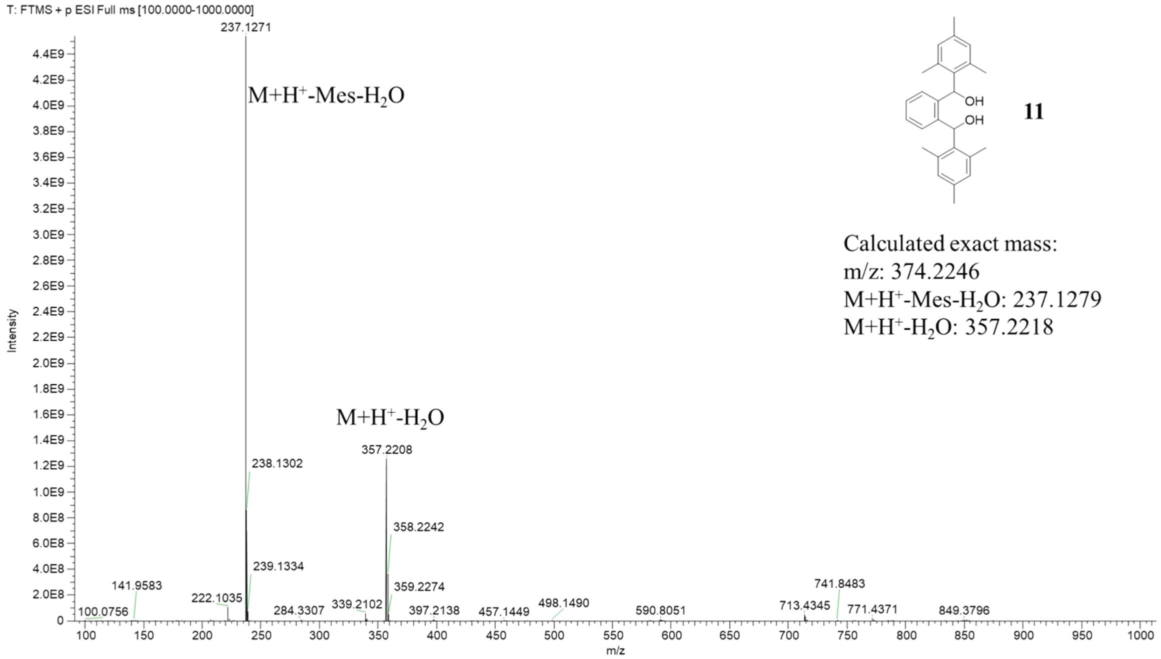
T: FTMS + p ESI Full ms [100.0000-1000.0000]
237.1271
M+H$^+$-Mes-H_2O
M+H$^+$-H_2O
357.2208
238.1302
358.2242
239.1334
359.2274
100.0756
141.9583
222.1035
284.3307
339.2102
397.2138
457.1449
498.1490
590.8051
713.4345
741.8483
771.4371
849.3796
Intensity
m/z
OH
OH
11
Calculated exact mass:
m/z: 374.2246
M+H$^+$-Mes-H_2O: 237.1279
M+H$^+$-H_2O: 357.2218

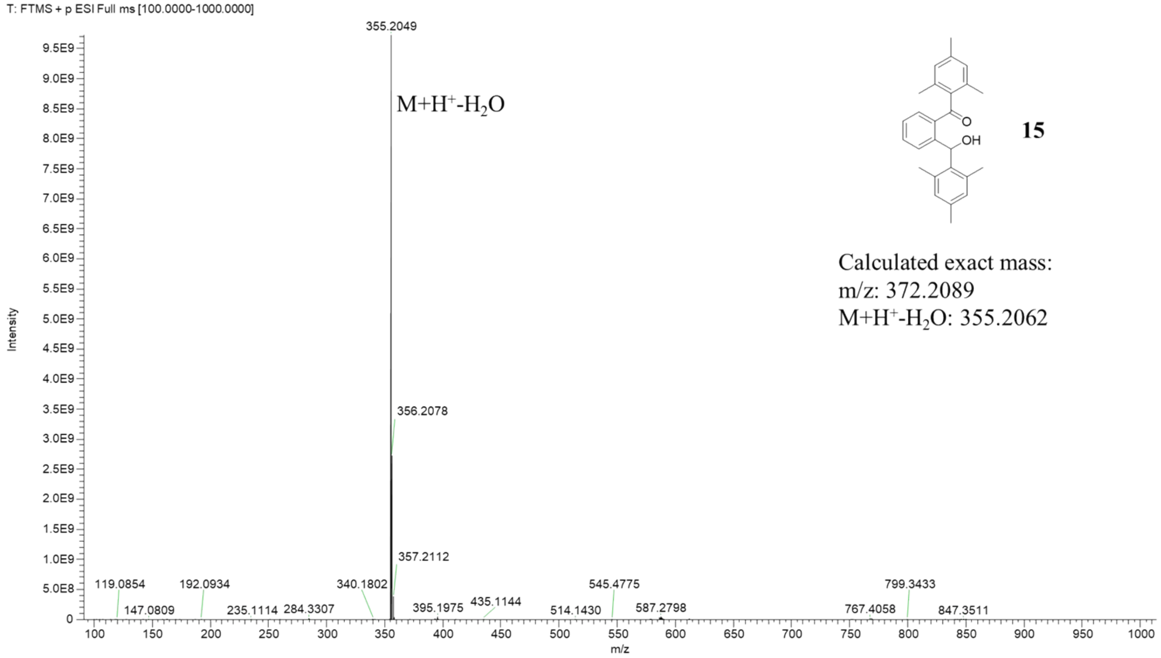
T: FTMS + p ESI Full ms [100.0000-1000.0000]
355.2049
M+H+-H2O
15
Calculated exact mass:
m/z: 372.2089
M+H+-H2O: 355.2062
356.2078
357.2112
119.0854
192.0934
340.1802
545.4775
799.3433
147.0809
235.1114
284.3307
395.1975
435.1144
514.1430
587.2798
767.4058
847.3511
Intensity
m/z

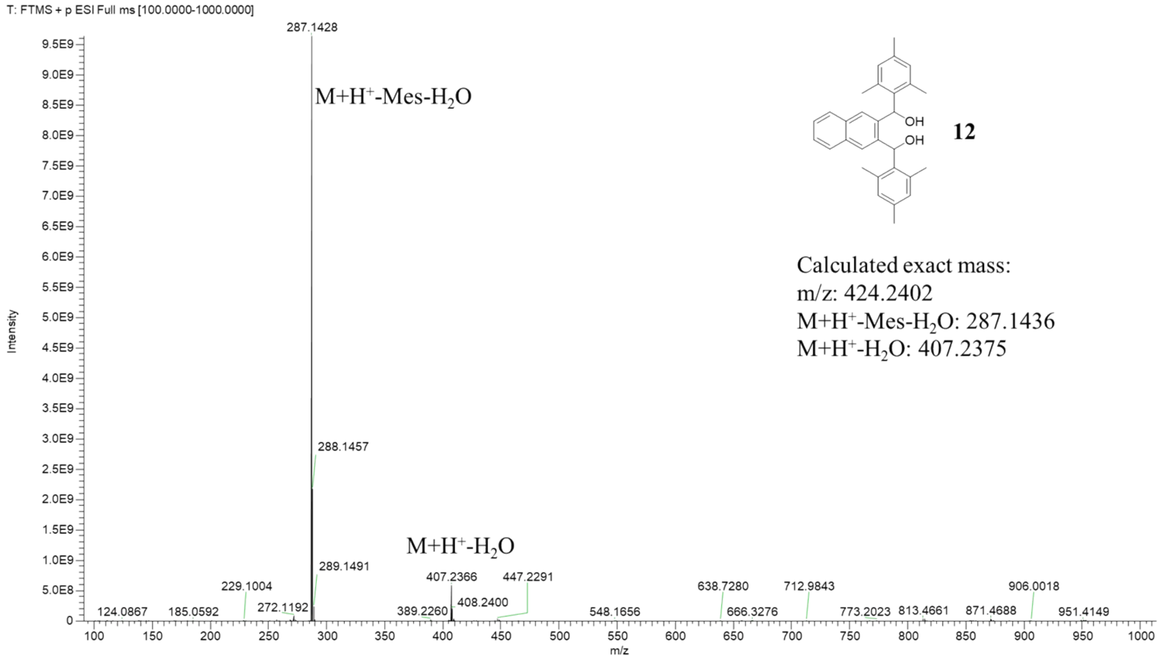
T: FTMS + p ESI Full ms [100.0000-1000.0000]
287.1428
M+H+-Mes-H2O
12
Calculated exact mass:
m/z: 424.2402
M+H+-Mes-H2O: 287.1436
M+H+-H2O: 407.2375
288.1457
M+H+-H2O
289.1491
407.2366
447.2291
229.1004
272.1192
124.0867
185.0592
389.2260
408.2400
548.1656
638.7280
666.3276
712.9843
773.2023
813.4661
871.4688
906.0018
951.4149
Intensity
m/z

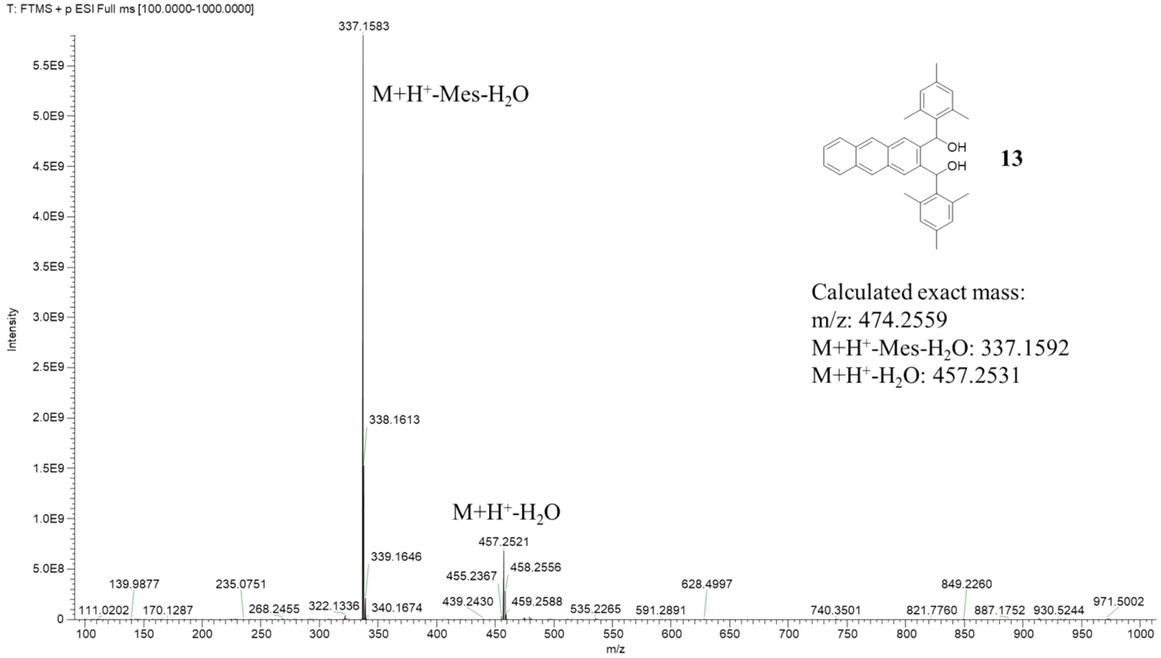
T: FTMS + p ESI Full ms [100.0000-1000.0000]
M+H+-Mes-H2O
M+H+-H2O
Intensity
m/z
13
Calculated exact mass:
m/z: 474.2559
M+H+-Mes-H2O: 337.1592
M+H+-H2O: 457.2531

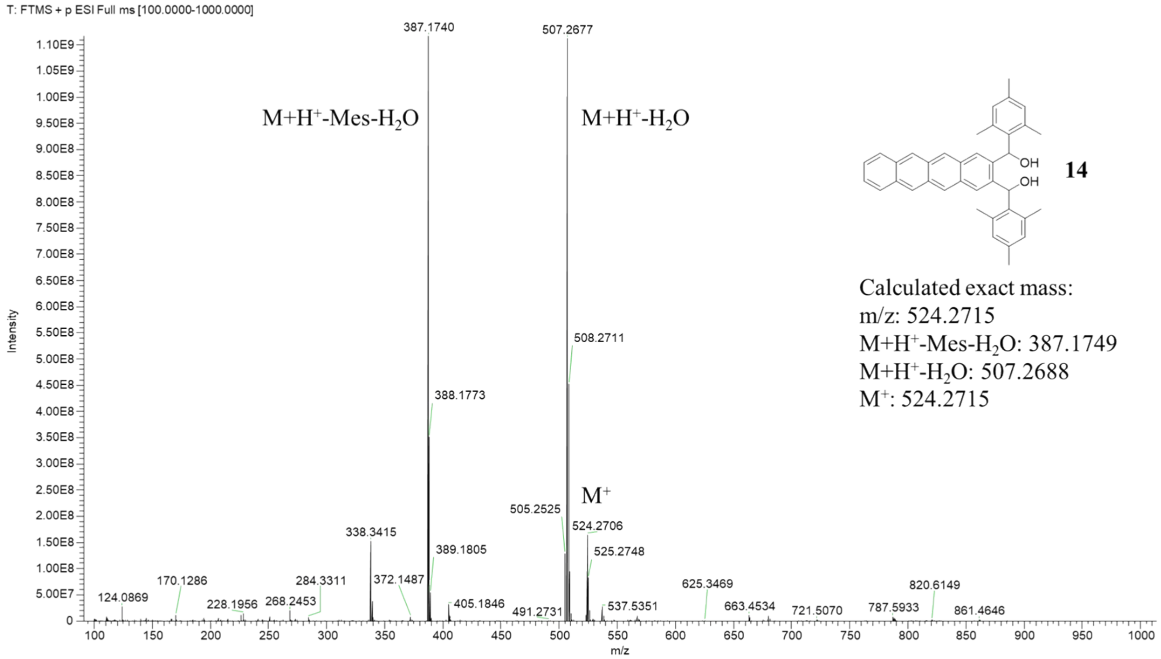
T: FTMS + p ESI Full ms [100.0000-1000.0000]
M+H+-Mes-H2O
M+H+-H2O
M+
Intensity
m/z
14
Calculated exact mass:
m/z: 524.2715
M+H+-Mes-H2O: 387.1749
M+H+-H2O: 507.2688
M+: 524.2715

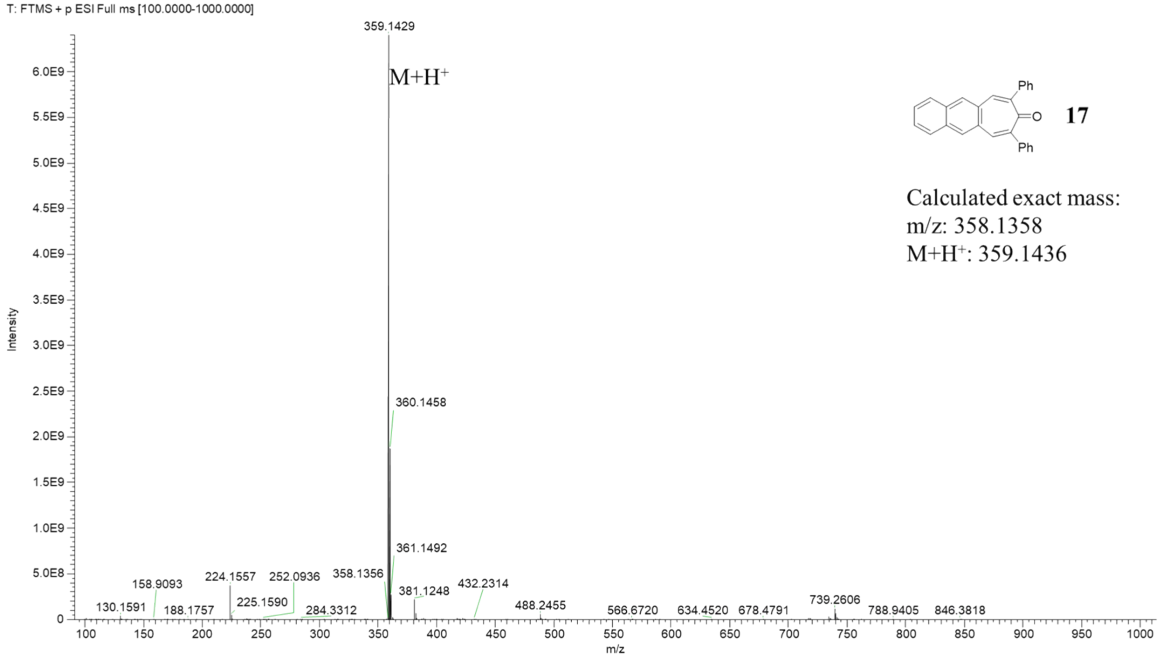
T: FTMS + p ESI Full ms [100.0000-1000.0000]
359.1429
M+H+
360.1458
361.1492
358.1356
Intensity
m/z
17
Ph
O
Ph
Calculated exact mass:
m/z: 358.1358
M+H+: 359.1436

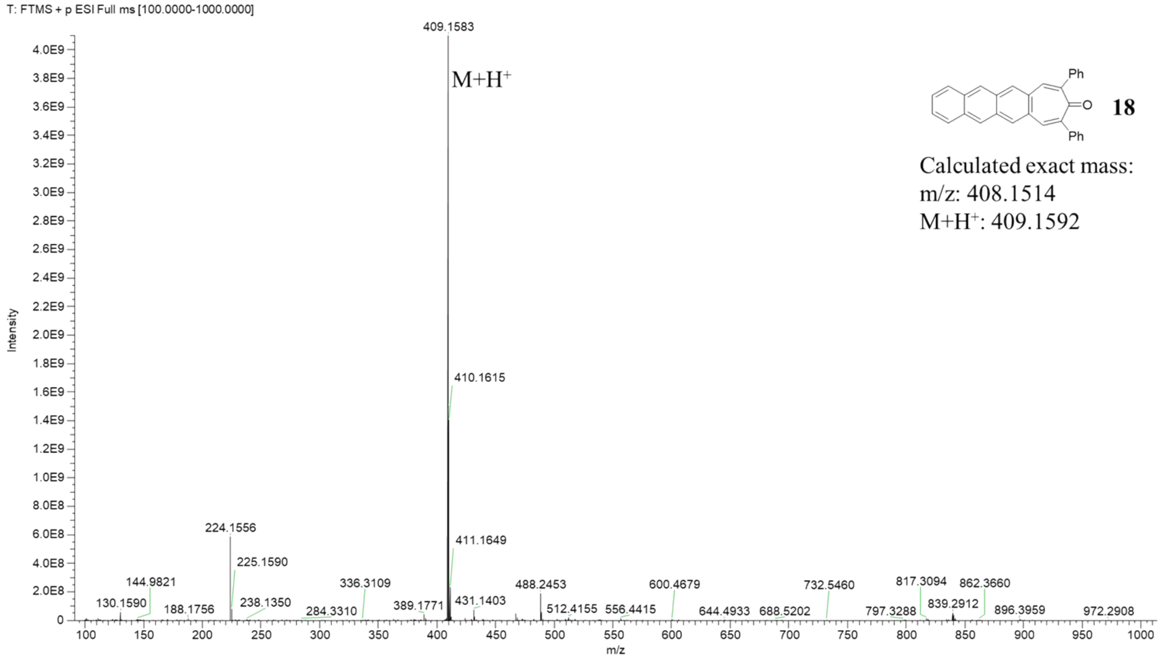
T: FTMS + p ESI Full ms [100.0000-1000.0000]
409.1583
M+H+
410.1615
411.1649
Intensity
m/z
18
Ph
O
Ph
Calculated exact mass:
m/z: 408.1514
M+H+: 409.1592

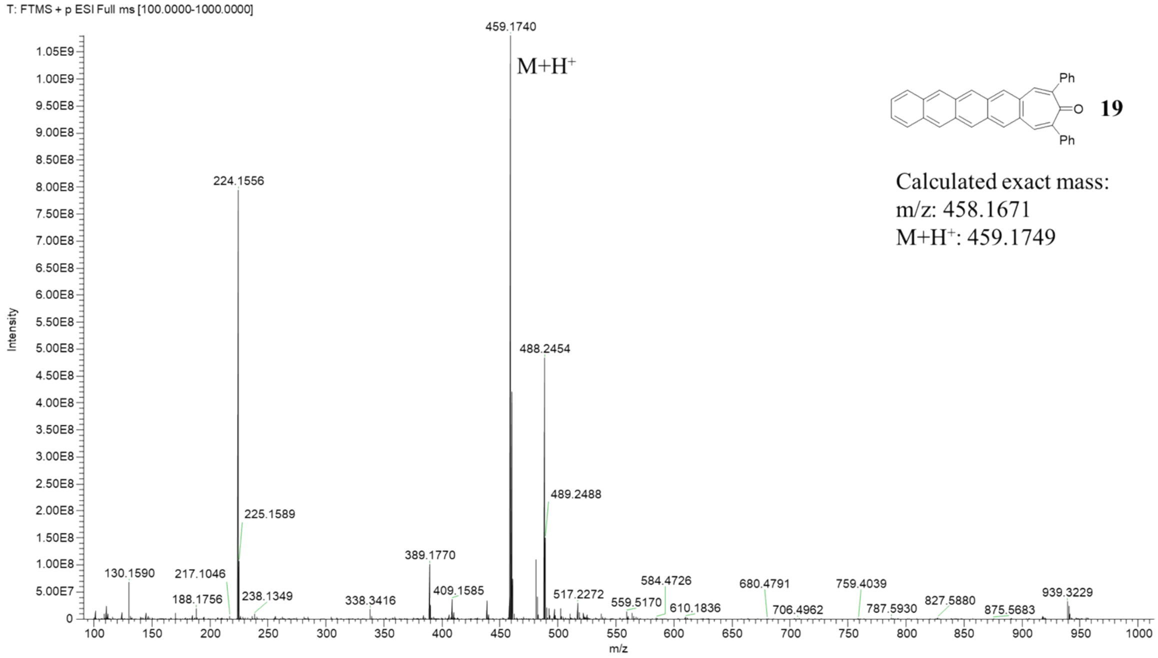

8. References

1 A. Mallouli and Y. Lepage, *Synthesis*, 1980, **1980**, 689–689.

2 R. C. Fuson, S. B. Speck and W. R. Hatchard, *J. Org. Chem.*, 1945, **10**, 55–61.

3 D. L. Crossley, C. D. Gabbutt, B. Mark Heron, P. Kay and M. Mogstad, *Dyes Pigments*, 2012, **95**, 62–68.

4 W. Ried and H. J. Schwenecke, *Chem. Ber.*, 1958, **91**, 566–572.
